# Bone Lesion‐Derived Extracellular Vesicles Fuel Prometastatic Cascades in Hepatocellular Carcinoma by Transferring ALKBH5‐Targeting miR‐3190‐5p

**DOI:** 10.1002/advs.202207080

**Published:** 2023-04-25

**Authors:** Shenqi Han, Lin Xue, Yi Wei, Tuying Yong, Wenlong Jia, Yongqiang Qi, Yiming Luo, Junnan Liang, Jingyuan Wen, Nana Bie, Huifang Liang, Qiumeng Liu, Zeyang Ding, Xiangliang Yang, Lu Gan, Zhao Huang, Xiaoping Chen, Bixiang Zhang

**Affiliations:** ^1^ Hepatic Surgery Center Tongji Hospital Tongji Medical College Huazhong University of Science and Technology Wuhan 430030 China; ^2^ Clinical Medical Research Center of Hepatic Surgery at Hubei Province Wuhan 430030 China; ^3^ Hubei Key Laboratory of Hepato‐Pancreatic‐Biliary Diseases Tongji Hospital Tongji Medical College Huazhong University of Science and Technology Wuhan 430030 China; ^4^ National Engineering Research Center for Nanomedicine College of Life Science and Technology Huazhong University of Science and Technology Wuhan 430074 China; ^5^ Key Laboratory of Organ Transplantation Ministry of Education Wuhan 430030 China; ^6^ Key Laboratory of Organ Transplantation National Health Commission Wuhan 430030 China; ^7^ Key Laboratory of Organ Transplantation Chinese Academy of Medical Sciences Wuhan 430030 China

**Keywords:** aptamer/liposome, bone metastasis, bone‐liver axis, exosome, liver cancer

## Abstract

Bone is the second leading metastatic site for hepatocellular carcinoma (HCC). Patients with HCC and bone metastasis suffer poor quality of life and reduced survival time. Extracellular vesicles (EVs) are widely involved in HCC formation and metastasis. However, the communication between primary HCC and bone lesions mediated by EVs remains unclear and the possible effect of bone metastasis on the progression of HCC remains largely unknown. Here, bone‐metastasized HCC‐derived EVs (BM‐EVs) are found to localize to orthotropic HCC cells and promote HCC progression. Mechanistically, miR‐3190‐5p (miR‐3190) is upregulated in intracellular HCC cells isolated from bone lesions as well as in their derived EVs. miR‐3190 in BM‐EVs is transferred into orthotopic tumor cells and enhances their metastatic capacity by downregulating AlkB homolog 5 (*ALKBH5*) expression. Decreased level of *ALKBH5* exacerbates the prometastatic characteristics of HCC by modulating gene expression in N6‐methyladenosine‐dependent and ‐independent ways. Finally, antagomir‐miR‐3190‐loaded liposomes with HCC affinity successfully suppress HCC progression in mice treated with BM‐EVs. These findings reveal that BM‐EVs initiate prometastatic cascades in orthotopic HCC by transferring ALKBH5‐targeting miR‐3190 and miR‐3190 is serving as a promising therapeutic target for inhibiting the progression of HCC in patients with bone metastasis.

## Graphical abstract

Bone‐metastasized hepatocellular carcinoma (HCC) derived extracellular vesicles localize to orthotopic HCC cells and target AlkB homolog 5 (ALKBH5) by transferring miR‐3190‐5p. Repressed *ALKBH5* regulates a cluster of metastasis‐related genes in N6‐methyladenosine‐dependent and ‐independent manners, which results in prometastatic cascades initiation. Furthermore, targeting miR‐3190‐5p by antagomir‐loaded liposomes with HCC affinity suppresses HCC progression.

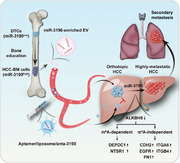

## 1 Introduction

Bones are one of the most common sites for cancer metastasis.^[^ ^1^ ^]^ Patients with cancer and bone lesions suffer from skeleton‐relevant events and dismal prognosis. In addition to the intrinsic characteristics of cancer cells, the interaction between the bone microenvironment and cancer cells contributes to the formation and progression of bone lesions.^[^ ^2^ ^]^ Cancer cells can disturb the skeletal remodeling process, initiating a vicious cycle that supports the development of disseminated tumor cells (DTCs) in bone.^[^ ^3^ ^]^ Notably, the bone microenvironment also reprograms tumor cells, conferring them with characteristics that are distinct from the primary cancer to facilitate secondary metastasis.^[^ ^4^ ^]^ These breakthroughs can help to elucidate cancer bone metastasis cascades and reform therapeutic approaches for patients.^[^ ^5^ ^]^

Despite its increasing incidence and poor prognosis, skeletal involvement in hepatocellular carcinoma (HCC) has not been given its due attention. The pathological and molecular mechanisms underlying the initiation and progression of bone metastasis in HCC are largely unknown. In our previous study, we established a reproducible animal model of HCC bone metastasis and isolated a series of HCC subpopulations with distinct bone metastatic abilities. We found that long noncoding RNA (lncRNA) H19 induced epithelial to mesenchymal transition (EMT) of HCC cells by sponging microRNA‐200b‐3p and aggravated osteolytic bone remodeling by reducing osteoprotegerin expression via the inactivation of p38 mitogen‐activated protein kinase (MAPK) signaling.^[^ ^6^ ^]^ In addition, the ring finger protein 219/*α*‐catenin/lectin galactoside‐binding soluble 3 axis was found to be a druggable target for inhibiting HCC bone metastasis progression and relieving skeleton‐related events.^[^ ^7^ ^]^ Despite these findings, therapeutic approaches for HCC bone metastasis are limited and are primarily palliative.^[^ ^8^ ^]^ In fact, patients with HCC and bone metastasis displayed shorter overall survival than those without bone metastasis.^[^ ^9^ ^]^ Additionally, even for patients who undergo radical surgery to remove primary tumors, bone lesions potentiate recurrence and compromise the survival of patients with HCC.^[^ ^10^ ^]^ These clinical facts indicate the impact of bone lesions on HCC progression. Increasing evidence implies that the bone‐liver axis contributes to liver disease progression.^[^ ^11^ ^]^ A recent study demonstrated that the elevated expression of hepatic serine/threonine protein phosphatase 2A catalytic subunit *α* during hepatic osteodystrophy inhibited the expression of lecithin‐cholesterol acyltransferase (*LCAT*) by dephosphorylating upstream transcription factor 1. The decreased level of *LCAT* led to the disruption of bone homeostasis and exacerbated liver fibrosis by restraining the transport of cholesterol from the bone to the liver.^[^ ^12^ ^]^ In HCC, low preoperative bone mineral density was found to be an independent risk factor for HCC mortality after surgery.^[^ ^13^ ^]^ However, the effect of the bone‐liver axis on the outcome and progression of patients with HCC and bone metastasis remains unclear.

Extracellular vesicles (EVs) are lipid bilayer membrane‐bound particles that include exosomes, microvesicles, and apoptotic bodies.^[^ ^14^ ^]^ During cancer progression, EVs modulate cell growth, mobility, angiogenesis, and formation of premetastatic niches (PMNs) by transferring bioactive cargoes.^[^ ^15^ ^]^ For HCC, EVs are a promising biomarker for the diagnosis of early HCC and a predictor for prognosis.^[^ ^16^ ^]^ HCC‐derived exosomal miR‐1247‐3p activated cancer‐associated fibroblasts (CAFs) and prepared PMNs in the lung, which promoted lung metastasis.^[^ ^17^ ^]^ In addition, CAFs can transfer miR‐320a to suppress HCC cell proliferation and metastasis.^[^ ^18^ ^]^ Nevertheless, the role of EVs in HCC metastasis, particularly the impact of EVs derived from metastatic sites on primary cancer progression is largely unknown.

AlkB homolog 5 (ALKBH5) is an RNA demethylase that modifies N6‐methyladenosine (m^6^A).^[^ ^19^ ^]^ In our recent studies, ALKBH5 downregulated lncRNA LINC02551 and progestin and adipoQ receptor 4 in an m^6^A‐dependent manner to inhibit HCC growth and metastasis.^[^ ^20^ ^]^ Chen et al. also found that ALKBH5 acted as a tumor suppressor in HCC formation and progression by facilitating the degradation of LYPD1, NCBI Gene ID: 116372, full name: LY6/PLAUR domain containing 1 via m^6^A modification.^[^ ^21^ ^]^ However, ALKBH5 was also reported to promote HCC progression by forming a positive‐feedback loop with Hepatitis B Virus X protein, and recruiting programmed cell death ligand 1^+^ macrophage infiltration.^[^ ^22^ ^]^ Interestingly, besides its function as an m^6^A eraser, Zhu et al. demonstrated that ALKHB5 regulated the epidermal growth factor receptor (EGFR) expression by interacting with human antigen R (HuR) protein, which was unrelated to the m^6^A function of ALKBH5.^[^ ^23^ ^]^

In this study, we found that bone‐metastasized HCC‐derived EVs (BM‐EVs) could localize to orthotopic HCC sites and promote HCC progression by transferring ALKBH5‐targeting miR‐3190‐5p (miR‐3190). Moreover, the targeted delivery of miR‐3190 antagomir (anta‐3190) with HCC affinity inhibited BM‐EV‐induced cancer progression.

## 2 Results

### 2.1 EVs Secreted by Bone‐Metastasized HCC Localize to Orthotopic Liver Tumor

**Figure 1:**
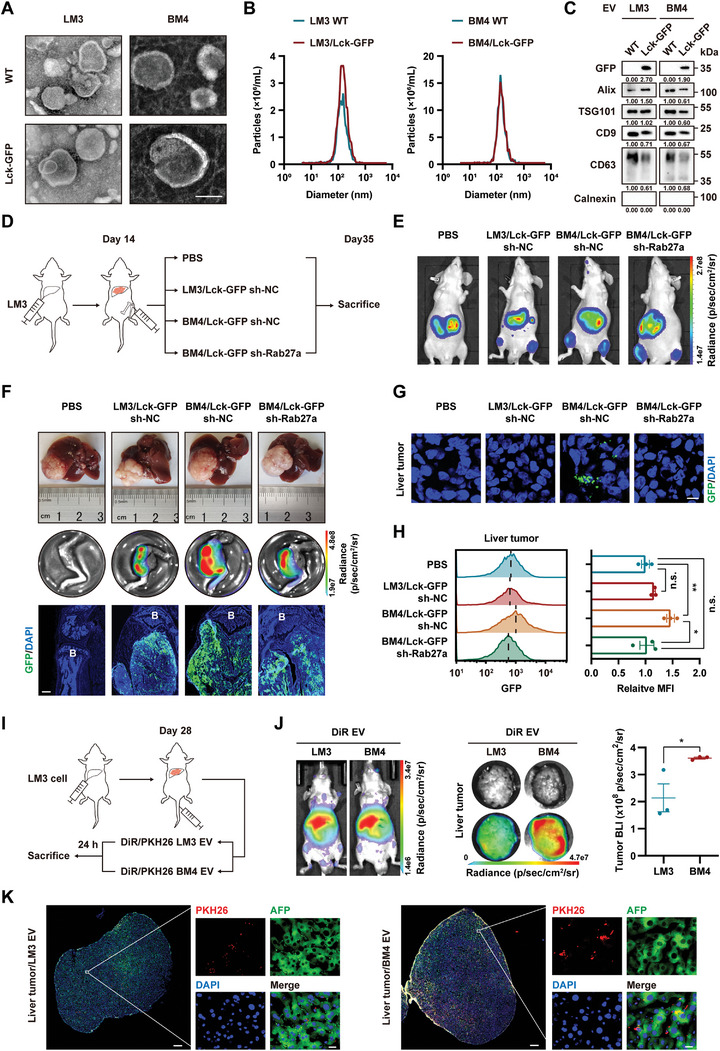
EVs secreted by bone‐metastasized HCC localize to orthotopic liver tumor. A,B) LM3 and BM4 cells were transfected with Lck‐GFP lentivirus. Characterization of EVs collected from the indicated HCC cells by A) TEM and B) NTA. Scale bar, 100 nm in (A). C) Western blot analysis for the expression of GFP, the indicated EV markers and the exclusion marker (Calnexin) in EVs. D–H) Mice were inoculated with LM3 cells in their livers. Fourteen days later, bone lesions were established by intratibial injection of the indicated cells (PBS as blank control) (*n* = 6). D) Schematic diagram of HCC animal model with bone lesion. E) Representative images of BLI intensity in mice bearing liver and bone tumor burden. F) Representative gross appearance of LM3 liver tumors, BLI examination, and GFP signaling of bone lesions in the indicated groups. Blue, 4′,6‐diamidino‐2‐phenylindole (DAPI); green, GFP. Scale bar, 500 µm. G) Representative confocal images of GFP signaling in orthotopic liver tumors from mice bearing Lck‐GFP cells. Blue, DAPI; green, GFP. Scale bar, 10 µm. H) Flow cytometry analysis of GFP signaling in xenograft tumor tissues in liver (*n* = 3). The fluorescence intensities were normalized to the PBS group. I–K) Mice bearing orthotopic LM3 tumors were injected with DiR‐ or PKH26‐stained EVs (*n* = 3). I) Schematic diagram of HCC animal model with tail vein injection of EVs. J) Representative images and quantification of BLI signaling in mice and excised liver tumor. K) Representative confocal images of liver tumor sections. Blue, DAPI; green, alpha fetoprotein (AFP); red, PKH26. Scale bar, 500 µm (low magnification), 20 µm (high magnification). Data are shown as mean ± standard error of mean (SEM). *P < 0.05, **P < 0.01, Student's *t*‐test. WT: wild‐type; EV, extracellular vesicle; BM4, LM3‐BM4; NC, negative control; sh, small hairpin RNA; B, bone; n.s., no significance; MFI, mean fluorescence intensity.

Considering the critical role of EVs in the communication between distant organs, we wondered whether BM‐EVs could localize to orthotopic HCC and participate in HCC progression. HCC‐LM3 (LM3) and its bone‐metastasized progeny LM3‐BM4 (BM4) cells were transduced with lentivirus carrying a lymphocyte protein tyrosine kinase‐green fluorescent protein(Lck‐GFP) transgene to generate GFP^+^ EVs.^[^ ^24^ ^]^ Transmission electron microscopy (TEM), nanoparticle tracking analysis (NTA), and western blot analysis confirmed the typical structure, size, and biomarkers of the secreted EVs both in wild‐type (WT) and in Lck‐GFP transduced HCC cells (LM3/Lck‐GFP and BM4/Lck‐GFP) (**Figure** **1**A–C). Confocal images and flow cytometry analysis revealed that GFP^+^ EVs could be taken up by LM3 cells (Figure S1A,B, Supporting Information). HCC animal model with bone lesions was established by inoculating LM3 cells into the liver, followed by intratibial injection of phosphate buffered saline (PBS), negative control cells (LM3/Lck‐GFP sh‐NC or BM4/Lck‐GFP sh‐NC), or BM4/Lck‐GFP cells with *RAB27A* knockdown (sh‐Rab27a), which suppressed EV secretion (Figure 1D and Figure S1C,D, Supporting Information).^[^ ^25^ ^]^ Gross appearance and H&E staining of liver and bone, as well as bioluminescence imaging (BLI) and GFP signals in bone, confirmed the success of the animal model (Figure 1E,F and Figure S1E–I, Supporting Information). GFP signals were detected in orthotopic HCC cells in mice with BM4/Lck‐GFP sh‐NC cells, but not in the other groups, indicating Lck‐GFP‐EVs secreted by bone‐metastasized HCC (HCC‐BM) cells were taken up by orthotopic HCC cells (Figure 1G,H).

To avoid the confounding GFP signaling aroused from self‐seeding tumor cells,^[^ ^26^ ^]^ DiR‐ or PKH26‐labeled BM4 EVs were injected into mice bearing orthotopic tumors via the tail vein (Figure 1I). Enriched BLI signaling and fluorescent spots in liver tumors demonstrated that EVs could enter the liver via the circulation and be taken up by orthotopic HCC cells (Figure 1J,K).

### 2.2 EVs Derived from Bone‐Metastasized HCC Cells Promote HCC Progression

**Figure 2:**
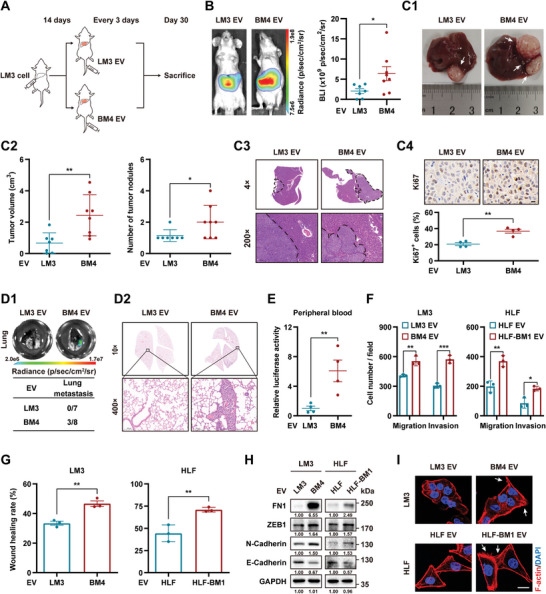
EVs derived from bone‐metastasized HCC cells promote HCC progression. A–E) LM3 cells were inoculated in liver of BALB/c nude mice. Fourteen days later, mice were injected with LM3 EVs (*n* = 7) or BM4 (*n* = 8) every 3 d. A) Diagram of orthotopic HCC animal model treated with EVs. B) Representative images and quantification of BLI intensity in mice. C) Postmortem examination of orthotopic liver tumor. C1) Representative macroscopic images of liver tumor. White arrows, orthotopic tumors. C2) Quantification of tumor volume and nodules. C3) Representative H&E staining of liver tumor edges. C4) Representative IHC staining of Ki67 and quantification of the percentages of tumor cells with Ki67^+^ (*n* = 4). Scale bar, 10 µm. D) Lung metastases evaluation. D1) Representative BLI (top) and quantification (bottom) of lung metastatic incidence. D2) Representative H&E staining of lungs. E) Relative luciferase activities in peripheral blood are shown as fold change relative to the LM3 EV‐treated group (*n* = 4). F–I) LM3 and HLF cells were treated with the indicated EVs for 48 h. F) Quantification of migrating and invading cells. G) Quantification of wound‐healing rate. H) Western blot analysis of the indicated EMT marker proteins. Glyceraldehyde 3‐phosphate dehydrogenase (GAPDH) as loading control. I) Representative confocal images of F‐actin staining. Red, F‐actin; blue, DAPI. White arrows, promotion of F‐actin formation. Scale bar, 20 µm. Data are shown as mean ± SEM. *P < 0.05, **P < 0.01, ***P < 0.001, Student's *t*‐test.

To explore the effect of BM‐EVs on HCC progression, we injected the same amount of LM3 EVs or BM4 EVs into mice with orthotopic LM3‐tumor via the tail vein every 3 d (**Figure** **2**A). Based on the BLI intensity, the tumor burden in the BM4 EV‐treated group was relatively larger (Figure 2B). Postmortem examination revealed increased liver tumor volume and nodules in mice treated with BM4 EVs compared to those in the control group (Figure 2C1,C2). More invasive tumor growth fronts and elevated proliferative ability were found in orthotopic liver tumors in the BM4 EV‐treated group by H&E and Ki67 staining compared with those treated with control EVs (Figure 2C3,C4). Ex vivo BLI examination and H&E staining showed that the incidence of lung metastasis was significantly increased by BM4 EVs stimulation (Figure 2D); however, no bone metastasis was observed in either group (Figure S2A, Supporting Information). In addition, we measured the luciferase intensity in peripheral blood to evaluate the number of circulating tumor cells (CTCs), which was remarkably elevated in mice injected with BM4 EVs (Figure 2E).

At the same time, LM3 and HLF is a human hepatocellular carcinoma cell line (JCRB0405) cells treated with their respective BM‐EVs (Figure S2B–F, Supporting Information) exhibited stronger migratory and invasive capacities, as evidenced by transwell and wound‐healing assays (Figure 2F,G and Figure S2G,H, Supporting Information). Western blot analysis showed that BM‐EVs treatment increased the expression of mesenchymal markers, N‐cadherin, fibronectin 1 (FN1), and zinc finger E‐box binding homeobox 1 (ZEB1), and downregulated the epithelial marker E‐cadherin in HCC cells compared to their respective control cells (Figure 2H). Additionally, HCC cells stimulated with BM‐EVs exhibited more mesenchymal morphology, characterized by F‐actin formation and cytoskeletal reorganization (Figure 2I). Notably, no significant difference was observed among the proliferation of the HCC cells treated with HCC‐EVs or BM‐EVs in the in vitro CCK8 assays (Figure S2I, Supporting Information), which may imply that the liver tumor microenvironment is involved in the effect of BM‐EVs on HCC proliferation. Therefore, the subsequent sections focus on the prometastatic functions of HCC‐BM‐EVs.

### 2.3 Prometastatic miR‐3190 Is Specifically Upregulated in HCC‐BM Cells and BM‐EVs

**Figure 3:**
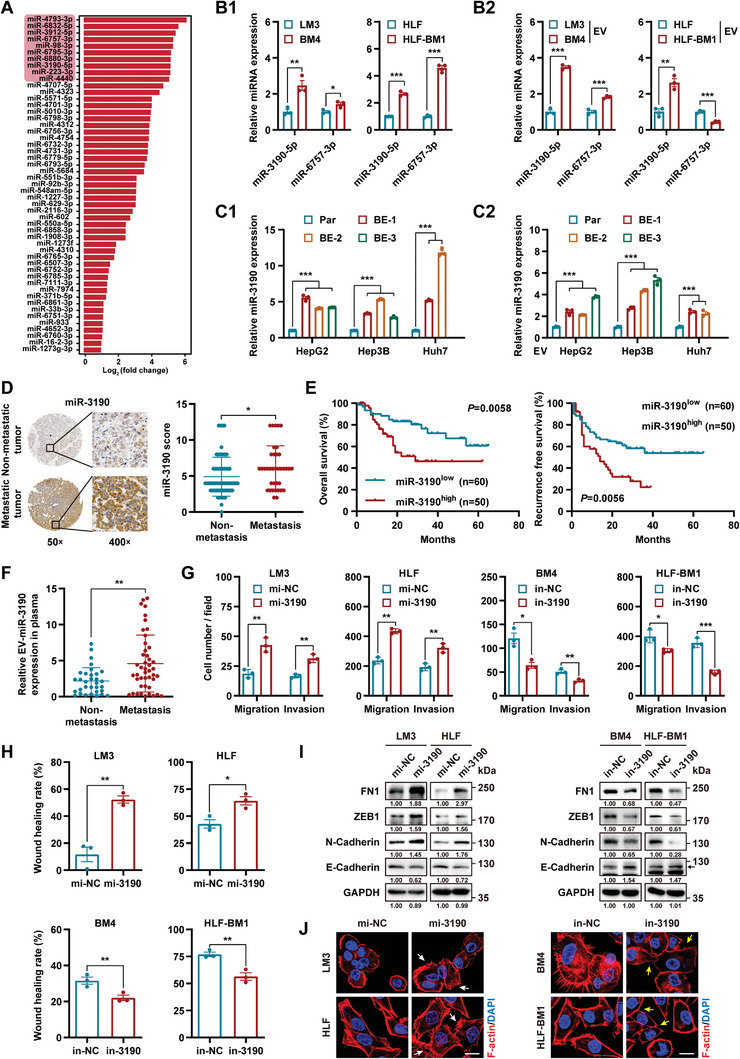
Prometastatic miR‐3190 is specifically upregulated in HCC‐BM cells and BM‐EVs. A) Upregulated miRNAs in BM4 cells compared to LM3 cells (Log_2_ fold change >1) in miRNA microarray. Top ten increased miRNAs are highlighted in red. B) qRT‐PCR analysis of miRNAs in B1) HCC cells and B2) EVs. Data are shown as fold change relative to control cells (LM3 and HLF) or their respective EVs. C) The expression of miR‐3190 was examined in cells and EVs of parent HCC (par) cell lines (HepG2, Hep3B, and Huh7) and their respective bone‐educated (BE) progenies. Data are shown as fold change relative to the parent cells or EVs. D) Representative images and quantification of miR‐3190 ISH staining in HCC specimens with (*n* = 37) or without (*n* = 73) metastases. E) Kaplan–Meier's analysis of overall survival and recurrence‐free survival in patients with HCC stratified by miR‐3190 expression. F) Quantification of miR‐3190 level in plasma EVs of patients with metastasized HCC (*n* = 45) or not (*n* = 32). G–J) HCC cells were transfected with miR‐3190 mimic or inhibitor for 48 h. G) Quantification of migrating and invading cells. H) Quantification of wound‐healing rate. I) Western blot analysis of the indicated EMT markers. GAPDH as loading control. J) Representative confocal images of F‐actin staining. Red, F‐actin; blue, DAPI. White arrows, promotion of F‐actin formation; yellow arrows, suppression of F‐actin formation. Scale bar, 20 µm. Data are shown as mean ± SEM. *P < 0.05, **P < 0.01, Student's *t*‐test in (B–D, and F–H), log‐rank test in (E). mi, mimic; in, inhibitor.

By interacting with the local microenvironment, DTCs can display characteristics that are distinct from those of primary cancer cells.^[^ ^4^, ^27^ ^]^ BM4 and HLF‐BM1 cells displayed short bone metastasis‐free survival time, increased skeletal tumor burden, aggravated osteolytic lesions, and enhanced maturation of preosteoclasts compared with their respective ancestor cells (LM3 and HLF) (Figure S3A–F, Supporting Information). In cancers, miRNAs and proteins within circulating EVs are highly enriched cargoes and well‐recognized players for cancer progression.^[^ ^28^ ^]^ We cultured LM3 cells by RNase A‐ or proteinase K‐treated EVs. Transwell assays demonstrated that the enhanced mobility induced by BM4 EVs was abrogated after RNase A treatment, which highlighted the crucial role of EV‐loaded RNAs (Figure S4A, Supporting Information). Therefore, we conducted a microRNA (miRNA) microarray to identify differentially expressed miRNAs between BM4 and LM3 cells, which may lead to distinct functions of the EVs secreted by these cells. The results showed that 51 miRNAs were significantly upregulated (Log_2_ fold change >1) (**Figure** **3**A and Table S2, Supporting Information). Among the 10 miRNAs with the largest fold changes, two miRNAs were confirmed to be upregulated in both HCC‐BM cell lines and only miR‐3190 was found to be highly expressed in BM‐EVs compared to the EVs of control cells (Figure 3B and Figure S4B, Supporting Information). Moreover, in another three HCC cells, miR‐3190 was consistently and obviously elevated in HCC bone‐educated cells and their corresponding EVs compared with parental HCC cells and their EVs, but not in lung‐educated cells and EVs (Figure 3C and Figure S4C, Supporting Information).

We then explored the clinical relevance and biological function of miR‐3190 in HCC. In situ hybridization (ISH) staining showed that miR‐3190 was remarkably higher in patients of HCC with metastases than in those without metastases (Figure 3D and Figure S4D, Supporting Information). Chi‐square analysis indicated that the expression of miR‐3190 positively correlated with microvascular invasion and satellite nodules (Table S3, Supporting Information). Kaplan–Meier analysis showed that patients of HCC with higher miR‐3190 levels had shorter survival and recurrence‐free survival times (Figure 3E). Moreover, the expression level of miR‐3190 was remarkably higher in circulating EVs from patients of HCC with metastasis than in those without metastasis (Figure 3F and Figure S4E–G, Supporting Information).

We then overexpressed miR‐3190 using a mimic (mi‐3190) and knocked it down using an inhibitor (in‐3190) (Figure S4H, Supporting Information). As demonstrated by transwell and wound‐healing assays, mobility was higher in HCC cells with relatively high miR‐3190 levels (Figure 3G,H and Figure S4I,J, Supporting Information). Western blot analysis revealed that the overexpression of miR‐3190 induced EMT, manifested through elevated levels of N‐cadherin, *FN1*, and *ZEB1* and impaired E‐cadherin expression (Figure 3I). Fluorescent staining of F‐actin revealed that the overexpression of miR‐3190 promoted actin cytoskeletal rearrangement in LM3 and HLF cells. However, the depletion of miR‐3190 in BM4 and HLF‐BM1 cells yielded the opposite results (Figure 3J).

### 2.4 Prometastatic Role of BM‐EVs Is Mediated by miR‐3190

**Figure 4:**
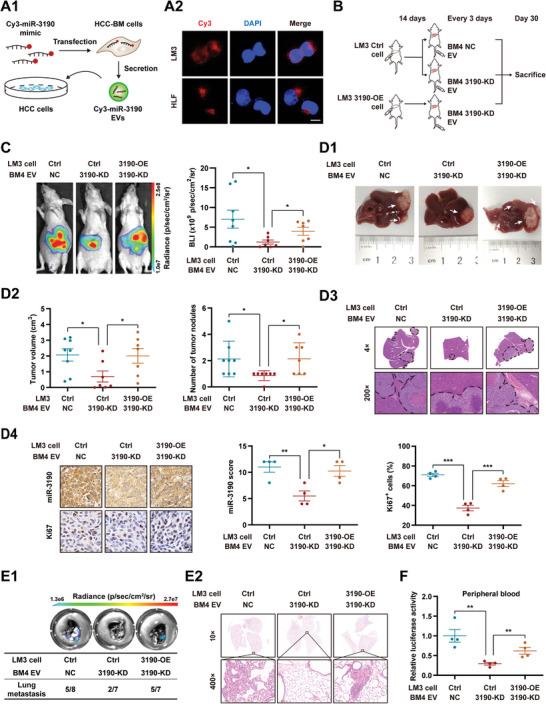
Prometastatic role of BM‐EVs is mediated by miR‐3190. A) HCC‐BM cells were transfected with Cy3‐labeled miR‐3190 mimic. Forty‐eight hours later, EVs were isolated from the supernatant and added to medium to culture HCC cells. A1) Diagram of experimental procedure for miR‐3190 transferring by EVs. A2) Representative fluorescence images of LM3 and HLF cells treated as in (A1). Red, Cy3; blue, DAPI. Scale bar, 10 µm. B–F) Mice were inoculated with LM3 cells with or without miR‐3190 overexpression (3190‐OE or Ctrl). Fourteen days later, EVs isolated from BM4 cells with or without miR‐3190 knocked down (3190‐KD or NC) were injected via tail vein every three days. *n* = 8 in control group, *n* = 7 in the other two groups. B) Schematic of orthotopic HCC xenograft model treated with different EVs in BALB/c nude mice. C) Representative images and quantification of BLI intensity in mice at the end point. D) Postmortem examination of orthotopic liver tumor. D1) Representative macroscopic images of liver tumor. White arrows, orthotopic tumors. D2) Quantification of tumor volume and nodules. D3) Representative H&E staining of liver tumor. D4) Representative images and quantification of ISH and IHC staining of liver tumor slides (*n* = 4). Scale bar, 10 µm. E) Lung metastases evaluation. E1) Representative ex vivo BLI (top) and quantification (bottom) of lung metastatic incidence. E2) Representative images of H&E staining of lungs. F) Relative luciferase activities in peripheral blood are shown as fold change relative to the control group (*n* = 4). Data are shown as mean ± SEM. *P < 0.05, **P < 0.01, ***P < 0.001, Student's *t*‐test. Ctrl, control; OE, overexpression; NC, negative control; KD, knockdown.

To explore whether BM‐EVs carrying miR‐3190 can be delivered into HCC cells, we transfected BM4 and HLF‐BM1 cells with Cy3‐tagged mi‐3190 and then cultured LM3 and HLF cells with the BM‐EVs secreted by these cells (**Figure** **4**A1). Fluorescence images showed significant enrichment of Cy3 signaling in the cytoplasm of the recipient HCC cells (Figure 4A2). Moreover, quantitative real‐time polymerase chain reaction (qRT‐PCR) analysis showed that the expression of miR‐3190 was upregulated in HCC cells treated with BM‐EVs compared to HCC‐EVs, which was dependent on EV uptake rather than endogenous miRNA transcription (Figure S5A,B, Supporting Information). These results suggest that BM‐EVs can deliver miR‐3190 to HCC cells.

Lentivirus was used to stably overexpress miR‐3190 (3190‐OE) in LM3 and HLF cells, or knockdown miR‐3190 (3190‐KD) in BM4 and HLF‐BM1 cells (Figure S5C, Supporting Information). The abundance of miR‐3190 in EVs was found to be consistent with that of their host cells (Figure S5D, Supporting Information). The orthotopic HCC model was established by inoculating LM3/control (LM3/Ctrl) or LM3/3190‐OE cells into BALB/c nude mice. Fourteen days later, the mice were intravenously injected with EVs isolated from BM4/negative control (BM4/NC) or BM4/3190‐KD cells every 3 d (Figure 4B). BLI examination, postmortem H&E and ISH/ immunohistochemistry (IHC) staining of the liver and lung, and the quantification of CTCs showed that the tumor‐promoting effects of BM4 EVs were impaired by ablating miR‐3190, and these effects were rescued by enhancing the expression of miR‐3190 in orthotopic HCC cells (Figure 4C–F). In addition, transwell and wound‐healing assays showed that HCC mobility was impaired after ablating miR‐3190 in BM4 EVs; however, enhancing cellular expression of miR‐3190 blunted these effects. Similar results were observed in the HLF cells (Figure S5E,F, Supporting Information).

### 2.5 ALKBH5 Is the Downstream Effector of BM‐EV‐Loaded miR‐3190

**Figure 5:**
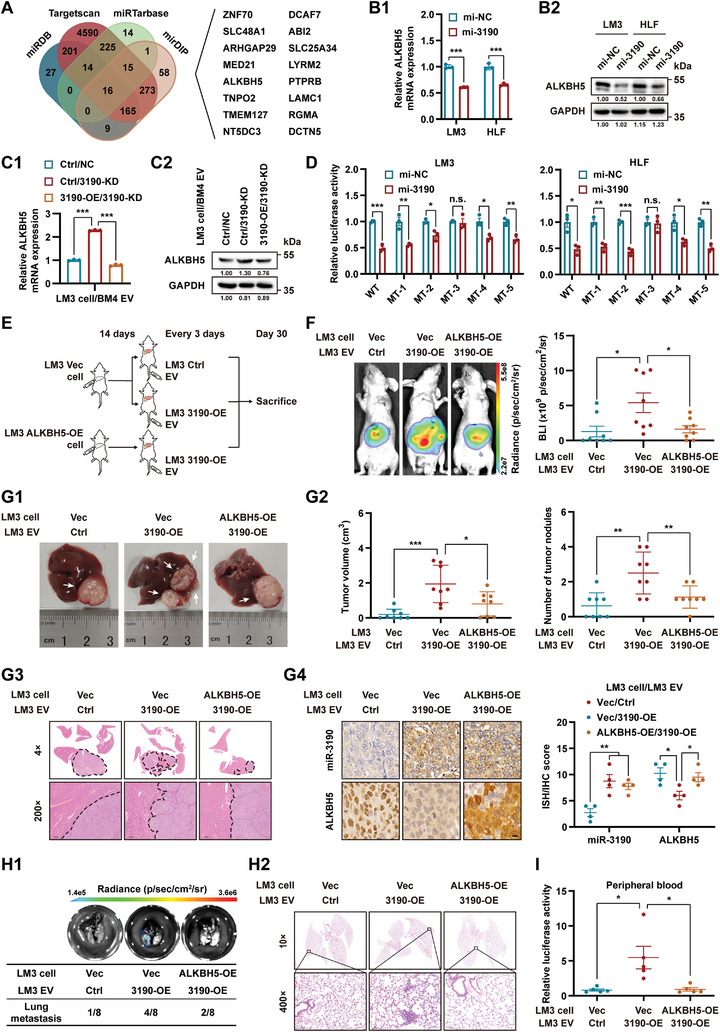
ALKBH5 is the downstream effector of BM‐EV‐loaded miR‐3190. A) Venn diagram for overlapping candidate target genes of miR‐3190 from four bioinformatics websites. B) HCC cells were transfected with miR‐3190 mimic for 48 h. B1) qRT‐PCR and B2) western blot analyses of *ALKBH5* in the indicated cells. Data are shown as fold change relative to HCC cells transfected with negative control mimic (mi‐NC) in (B1). C) EVs isolated from BM4 cells with or without miR‐3190 knockdown (3190‐KD or NC) were used to treat LM3 cells with or without miR‐3190 overexpression (3190‐OE or Ctrl). C1) qRT‐PCR and C2) western blot analyses of *ALKBH5* expression in the indicated cells. Data are shown as fold change relative to the control group in (C1). D) Dual luciferase reporter assay in HCC cells transfected with miR‐3190 mimic together with plasmids expressing the wild‐ or mutant‐3’UTR of *ALKBH5*. Data were normalized to the cells transfected with negative control mimic. E–I) Mice were inoculated with LM3 cells with or without *ALKBH5* overexpression (ALKBH5‐OE or Vec). Fourteen days later, EVs isolated from LM3 cells with or without miR‐3190 overexpression (3190‐OE or Ctrl) were injected via tail vein every 3 d (*n* = 8). E) Schematic of orthotopic HCC xenograft model treated with different EVs in BALB/c nude mice. F) Representative images and quantification of BLI intensity in mice at end point. G) Postmortem examination of orthotopic liver tumor. G1) Representative macroscopic images of liver tumor. White arrows, orthotopic tumors. G2) Quantification of tumor volume and nodules. G3) Representative H&E staining of liver tumor edges. G4) Representative images and quantification of ISH and IHC staining of liver tumor sections in indicated groups (*n* = 4). Scale bar, 10 µm. H) Lung metastases evaluation. H1) Representative ex vivo BLI (top) and quantification (bottom) of lung metastatic incidence. H2) Representative H&E staining of lungs. I) Relative luciferase activities in peripheral blood are shown as fold change relative to the control group (*n* = 5). GAPDH as loading control in (B2) and (C2). Data are shown as mean ± SEM. *P < 0.05, **P < 0.01, ***P < 0.001, Student's *t*‐test. WT, wild‐type; MT, mutant‐type.

Four bioinformatic tools were used to predict the downstream effectors of miR‐3190 (**Figure** **5**A). After detection at both the mRNA and protein levels, only *ALKBH5* was found to be negatively regulated by miR‐3190 in HCC cells (Figure 5B and Figure S6A,B, Supporting Information). *ALKBH5* mRNA and miR‐3190 in clinical HCC samples were observed to be significantly negatively correlated (Figure S6C, Supporting Information). Moreover, the expression of *ALKBH5* in LM3 cells treated with BM4/3190‐KD EVs exceeded that in the cells treated with control EVs and this regulation was abolished by upregulating miR‐3190 in LM3 cells (Figure 5C). IHC staining showed that the expression of ALKBH5 in orthotopic liver tumors treated with distinct EVs was negatively correlated with miR‐3190 levels (Figure S6D, Supporting Information). Five potential binding sites for miR‐3190 within the 3’ untranslated region (3’UTR) of *ALKBH5* were predicted (Table S4, Supporting Information). The dual luciferase reporter assay showed that miR‐3190 attenuated the relative luciferase activity of *ALKBH5* 3’UTR, while mutating binding site 3 abolished this regulation (Figure 5D), indicating that binding site 3 was responsible for the interaction between miR‐3190 and *ALKBH5* mRNA.

To explore the relevance of ALKBH5 in the prometastatic role of miR‐3190‐enriched EVs, we first examined the influence of ALKBH5 on HCC mobility. Gain‐ and loss‐of‐function assays demonstrated that ALKBH5 inhibited the mobility of HCC cells in vitro (Figure S7A–D, Supporting Information). We then established orthotopic mouse models by implanting LM3 cells with or without *ALKBH5* overexpression (ALKBH5‐OE or Vec) in the liver and subsequently injected EVs isolated from LM3 cells with or without miR‐3190 overexpression (3190‐OE or Ctrl) (Figure 5E). At the end point, BLI examination, postmortem H&E, and ISH/IHC staining of the liver or lung, along with CTCs quantification, showed that the enhanced proliferation and metastasis of orthotopic HCC induced by miR‐3190‐enriched EVs were restored by ectopic expression of *ALKBH5* in orthotopic HCC cells (Figure 5F–I and Figure S8A, Supporting Information). In addition, in vitro experiments revealed that downregulated *ALKBH5* and elevated cell mobility induced by miR‐3190‐enriched EVs was ablated in *ALKBH5*‐overexpressing HCC cells (Figure S8B–D, Supporting Information).

### 2.6 EV‐miR‐3190/ALKBH5 Axis Initiates Metastatic Cascades in m6A‐Dependent and ‐Independent Manners

**Figure 6:**
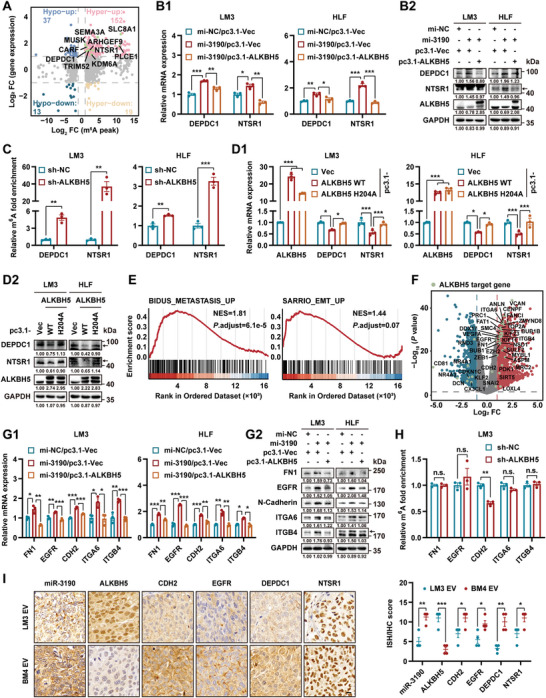
EV‐miR‐3190/ALKBH5 axis initiates metastatic cascades in m^6^A‐dependent and ‐independent manners. A) Four‐quadrant diagram for genes with altered expression (up or down) and m^6^A modification (hyper or hypo) between LM3/sh‐NC and LM3/sh‐ALKBH5 cells. Elevated metastatic‐related transcripts with increased m^6^A modification after knocking down *ALKBH5* were annotated. B) HCC cells with or without miR‐3190 transiently overexpression (mi‐3190 or mi‐NC) were cotransfected with pcDNA3.1‐ALKBH5 (pc3.1‐ALKBH5) or pcDNA3.1‐vector plasmid (pc3.1‐Vec) for 48 h. B1) qRT‐PCR and B2) western blot analyses of the indicated genes in HCC cells. Data are shown as fold change relative to HCC cells cotransfected with negative control mimic and pcDNA3.1‐vector plasmid in (B1). C) MeRIP assays after knocking down *ALKBH5* in HCC cells. Data are shown as fold change relative to negative control cells. D) HCC cells were transfected with pcDNA3.1‐ALKBH5 (pc3.1‐ALKBH5 WT) or pcDNA3.1‐ALKBH5(H204A) (pc3.1‐ALKBH5 H204) plasmid for 48 h. D1) qRT‐PCR and D2) western blot analyses for the expression of the indicated genes in HCC cells. Data are shown as fold change relative to HCC/pc3.1‐Vec cells in (D1). E) GSEA of metastatic gene clusters in differentially expressed genes (DEGs) from RNA‐seq data. F) The enriched metastasis‐related genes were spotted in the volcano plot of DEGs. G) HCC cells were treated as in (B). G1) qRT‐PCR and G2) western blot analyses of the indicated genes in HCC cells. Data are shown as in (B). H) MeRIP assays of the indicated transcripts with m^6^A modification in LM3/sh‐NC and LM3/sh‐ALKBH5 cells. Data are shown as fold change relative to negative control cells. I) Representative images and quantification of ISH and IHC staining in mice tumor tissues treated with different EVs in Figure 2 (*n* = 4). Scale bar, 10 µm. GAPDH as loading control in (B2, D2, and G2). Data are shown as mean ± SEM. *P < 0.05, **P < 0.01, ***P < 0.001, Student's *t*‐test. FC, fold change.

ALKBH5 is one of the m^6^A eraser that affects mRNA export and metabolism.^[^ ^19^ ^]^ To elucidate the molecular mechanism underlying the suppressive role of ALKBH5 in HCC metastasis, we performed methylated RNA immunoprecipitation sequencing (MeRIP‐seq) and RNA‐sequencing (RNA‐seq) of LM3 cells with *ALKBH5* stably knocked down (sh‐ALKBH5) (Figure S9A, Supporting Information). Consistent with previous studies,^[^ ^29^ ^]^ the common m^6^A motif, GGAC, was highly enriched with m^6^A peaks in both ALKBH5‐deficient and control LM3 cells (Figure S9B, Supporting Information). A similar distribution of m^6^A peaks was observed in the two groups, primarily located in the coding sequence and 3’UTR, and these m^6^A modifications were predominately located near the stop codons (Figure S9C, Supporting Information). *ALKBH5* depletion resulted in 1457 upregulated and 717 downregulated genes (Log_2_ fold change >1, *P* < 0.05) (Figure S9D and Table S5, Supporting Information). To determine the m^6^A‐modified targets of ALKBH5, we mapped a quadrant chart by combining MeRIP‐seq and RNA‐seq data (**Figure** **6**A and Table S6, Supporting Information). Given the demethylation role of ALKBH5, we focused on transcripts with increased m^6^A modification after *ALKBH5* knockdown in LM3 cells. Ten well‐recognized prometastatic genes were selected for qRT‐PCR validation and the mRNA levels of domain containing 1 DEP domain containing 1 (*DEPDC1*) and neurotensin receptor 1 (*NTSR1*) were upregulated upon *ALKBH5* silencing and miR‐3190 overexpression in LM3 cells (Figure S9E, Supporting Information). Rescue assays further revealed that the regulation of miR‐3190 on *DEPDC1* and *NTSR1* were mediated by ALKBH5 (Figure 6B). Furthermore, m^6^A peaks were found to be more enriched in the 3’UTR of *DEPDC1* and *NTSR1* mRNA in LM3/sh‐ALKBH5 cells than in LM3/sh‐NC cells according to MeRIP‐seq (Figure S9F, Supporting Information). Anti‐ALKBH5 and anti‐m^6^A RNA immunoprecipitation (RIP) assays demonstrated that *DEPDC1* and *NTSR1* transcripts were bound by ALKBH5 and were m^6^A‐modified (Figure S9G,H, Supporting Information). Moreover, the abundance of m^6^A‐modified transcripts was increased after *ALKBH5* knockdown (Figure 6C). Overexpression of the wild type ALKBH5 instead of the reported catalytically inactive mutant ALKBH5 H204A^[^ ^30^ ^]^ reduced the expression of *DEPDC1* and *NTSR1* (Figure 6D). These data suggest that the expression of *DEPDC1* and *NTSR1* are modulated by ALKBH5 in an m^6^A‐dependent manner.

Recent studies have reported that some m^6^A regulators could affect tumor cellular process in m^6^A‐independent mechanisms.^[^ ^23^, ^31^ ^]^ Thus, we investigated the potential downstream targets regulated by ALKBH5 in RNA‐seq data. Gene Set Enrichment Analysis (GSEA) revealed that the differentially expressed genes (DEGs) were correlated with prometastatic gene signatures (Figure 6E). We then focused on the metastasis‐related genes enriched in GSEA and differentially expressed EMT genes (Figure 6F and Table S5, Supporting Information)^[^ ^32^ ^]^ and examined their regulation by ALKBH5 via qRT‐PCR (Figure S10A, Supporting Information). The verified downstream targets of ALKBH5 were evaluated in LM3 cells overexpressing miR‐3190 (Figure S10B, Supporting Information). A considerable number of candidate genes were regulated by both ALKBH5 and miR‐3190 in LM3 cells. Among the downstream genes, FN1, cadherin 2 (CDH2), EGFR, integrin subunit alpha 6 (ITGA6), and integrin subunit beta 4 (ITGB4), which are widely studied and are associated with cancer metastasis, were selected for further investigation. qRT‐PCR and western blot analyses demonstrated that the overexpression of *ALKBH5* reversed the increased expression of these genes induced by miR‐3190 mimic in LM3 and HLF cells (Figure 6G). To confirm whether ALKBH5 regulated the m^6^A modification of *FN1*, *EGFR*, *CDH2*, *ITGA6* or *ITGB4*, we performed MeRIP and RIP assays. MeRIP assays showed that m^6^A was enriched in these target transcripts compared to IgG group (Figure S10C, Supporting Information). Moreover, RIP assays showed ALKBH5 could bind these RNAs in LM3 cells (Figure S10D, Supporting Information). However, knocking down of *ALKBH5* could not regulate m^6^A modification in the transcripts of *FN1*, *EGFR*, *ITGA6* or *ITGB4*, and even downregulated *CDH2* m^6^A level (Figure 6H). These results suggested that ALKBH5 may regulate these genes expression in an m^6^A‐independent manner.

Furthermore, m^6^A levels were higher in HCC cells treated with BM‐EVs than in those treated with HCC‐EVs (Figure S10E, Supporting Information). Treatment with BM‐EVs significantly increased the expression of ALKBH5 target genes (Figure S10F, Supporting Information). In addition, the expression relevance of miR‐3190 and ALKBH5, as well as its downstream effectors, were verified by ISH/IHC staining for orthotopic HCC samples treated with distinct EVs (Figure 6I).

### 2.7 Therapeutic Effects of Targeting miR‐3190 by Aptamer/Liposome Delivery System

**Figure 7:**
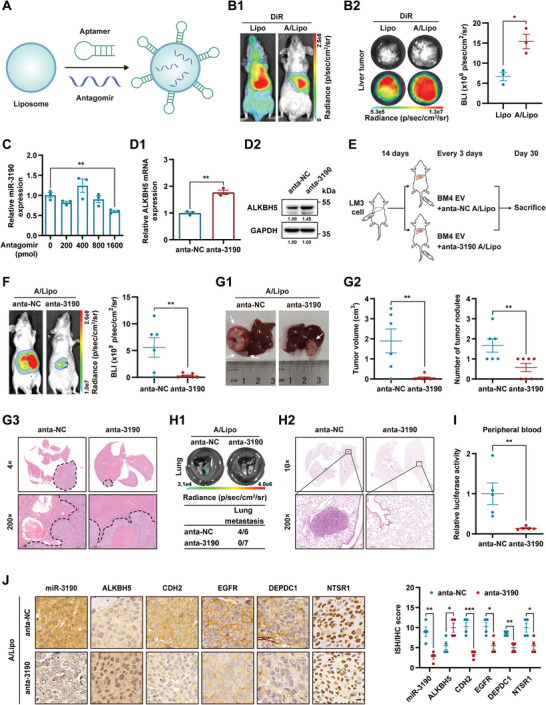
Therapeutic effects of targeting miR‐3190 by aptamer/liposome delivery system. A) Schematic of aptamer/liposome/antagomir delivery system. B) Mice bearing orthotopic LM3 liver tumors were injected with DiR‐stained liposome or aptamer/liposome (A/Lipo) via tail vein (*n* = 3). B1) Representative images of in vivo BLI examination. B2) Representative images of excised liver tumor and quantification of corresponding ex vivo BLI intensity. C) qRT‐PCR analysis of miR‐3190 levels in HCC cells transfected with A/Lipo (2 pmol: 1 µg) loaded with the indicated amount of antagomir‐miR‐3190 (anta‐3190) for 24 h. D,D1) qRT‐PCR and D2) western blot analyses of *ALKBH5* expression in HCC cells after treated with 1600 pmol anta‐3190 or negative control (anta‐NC) encapsulated in A/Lipo for 48 h. GAPDH as loading control in (D2). E–J) Mice were inoculated with LM3 cells for 14 d, then A/Lipo loaded with anta‐3190 (*n* = 7) or anta‐NC (*n* = 6) were injected with BM4 EVs via tail vein every three days. E) Schematic of orthotopic HCC xenograft model treated with A/Lipo/antagomir complex. F) Representative images and quantification of BLI intensity in mice at end point. G) Postmortem examination of orthotopic liver tumor. G1) Representative macroscopic images of liver tumor. White arrows, orthotopic tumors. G2) Quantification of tumor volume and nodules. G3) Representative H&E staining of liver tumor. H) Lung metastases evaluation. H1) Representative ex vivo BLI (top) and quantification (bottom) of lung metastatic incidence. H2) Representative images of H&E staining of lungs. I) Relative luciferase activities in peripheral blood are shown as fold change relative to the control group (*n* = 5). J) Representative images and quantification of ISH and IHC staining of HCC tumor slides (*n* = 4). Scale bar, 10 µm. Data are shown as fold change relative to HCC cells transfected with negative control antagomir in (C) and (D1). Data are shown as mean ± SEM. *P < 0.05, **P < 0.01, ***P < 0.001, Student's *t*‐test.

Given the prometastatic role of the miR‐3190/ALKBH5 axis driven by BM‐EVs, we aimed to deliver anta‐3190 into HCC cells using liposomes. To enhance the specificity for HCC cells, the liposomes were modified with the liver cancer tropic aptamer TLS11a^[^ ^33^ ^]^ (**Figure** **7**A). The aptamer‐modified liposome particles were characterized as spherical in shape and ≈100 nm in diameter by TEM and NTA analyses (Figure S11A,B, Supporting Information). The fluorescence spectrum displayed the maximal emission peak of A/Lipo at 522 nm, indicating that carboxyfluorescein (FAM)‐labeled aptamers (FAM‐A) were successfully attached to the liposomes (Figure S11C, Supporting Information). In mice with orthotopic xenografts of LM3 cells, BLI showed that DiR‐stained A/Lipo specifically aggregated in the liver area, while liposomes without aptamers were distributed throughout the body (Figure 7B1). The postmortem tumor sample exhibited more robust fluorescence intensity than that of the control group (Figure 7B2).

To examine the delivery capacity of the A/Lipo system, Cy5‐antagomir was loaded into the FAM‐A/Lipo. The intracellularly localized fluorescent spots and enhanced signaling intensities of FAM and Cy5 in LM3 cells treated with FAM‐A/Lipo/Cy5‐antagomir verified the antagomir delivery capacity of the A/Lipo system (Figure S11D,E, Supporting Information). To optimize the delivery efficiency of this system, HCC cells were treated with different ratios of A/Lipo. Flow cytometry assays showed that the 2:1 (pmol/µg) A/Lipo displayed the highest delivery efficiency in this system, despite an increase in the resorption of the aptamer (Figure S11F,G, Supporting Information). Furthermore, qRT‐PCR and western blot analyses showed that loading 1600 pmol antagomir in A/Lipo (125 pmol:62.5 µg) efficiently knocked down miR‐3190 expression and upregulated *ALKBH5* expression in LM3 cells (Figure 7C,D).

Orthotopic LM3 bearing mice were injected with EVs from BM4 cells via the tail vein every 3 d, accompanied by antagomiR‐NC (anta‐NC) or anta‐3190 loaded A/Lipo (Figure 7E). At the endpoint, mice treated with anta‐3190 exhibited less liver tumor burden, milder metastatic features, and lower lung metastatic incidence, as evidenced by BLI intensity, intrahepatic tumor size and numbers, H&E staining, micro‐metastases in lungs, and relative CTCs abundance (Figure 7F–I and Figure S11H, Supporting Information). In addition, continuous treatment with A/Lipo at the same frequency in healthy mice showed no statistical differences in weight, alanine aminotransferase and aspartate aminotransferase expression compared with those treated with PBS, indicating the minor liver and general toxicity of the A/Lipo complex (Figure S12A–F, Supporting Information). Moreover, the aforementioned regulation of targeted genes by EV‐encapsulated miR‐3190 was also verified in orthotropic liver tumors treated with A/Lipo/anta‐3190 (Figure 7J).

In addition, to confirm the prometastatic effects of miR‐3190 as a cargo of EVs, we used agomiR of miR3190 (ago‐3190) loaded with A/Lipo to mimic miR‐3190‐enriched EVs in circulation. The delivery capacity, optimized A/Lipo/agomiR ratio, and biological effects were explored using the approaches described above (Figure S13A–D, Supporting Information). We then established an orthotopic HCC mouse model using LM3 cells and injected A/Lipo containing agomir‐NC (ago‐NC) or ago‐3190 into mice via the tail vein (Figure S13E, Supporting Information). Mice treated with A/Lipo/ago‐3190 displayed a heavier tumor burden, more aggressive fronts, higher lung metastatic incidences, and increased CTC numbers (Figure S13F–I, Supporting Information).

## 3 Discussion

**Figure 8:**
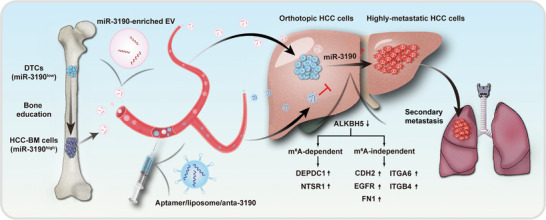
Schematic of bone‐liver axis mediated by miR‐3190‐enriched EVs. miR‐3190 is upregulated in HCC‐BM cells, leading to the enrichment of miR‐3190 in BM‐EVs. miR‐3190‐enriched BM‐EVs are taken up by orthotopic HCC cells and initiate expression of prometastatic gene cluster by inhibiting *ALKBH5*, which result in metastasis cascades. Furthermore, targeted delivery of anta‐3190 by A/Lipo suppresses BM‐EVs induced HCC progression. DTCs, disseminated tumor cells; anta, antagomir.

We demonstrated the tumor‐promoting effects of the bone‐liver axis mediated by BM‐EVs. Mechanistically, BM‐EVs promote orthotopic cancer progression by exacerbating the prometastatic capacity of HCC cells through transferring miR‐3190 (**Figure** **8**). Previous studies have revealed that cancer cells in circulation and in metastasized sites (“seeds”) could easily settle in their primary site, called “self‐seeding.”^[^ ^4^, ^26^ ^]^ Additionally, the metastatic derivatives exhibited higher seeding abilities than their parental counterparts. In this study, we demonstrated that the BM‐EVs readily settle in the liver and are taken up by orthotopic HCC cells, which may be inferred as “self‐seeding of EVs.” Moreover, EVs derived from bone‐metastasized cells exhibited higher enrichment in orthotopic tumors compared with EVs from their respective ancestor cells. The enhanced self‐seeding effects of BM‐EVs were similar with tumor cells.^[^ ^26^ ^]^ This makes sense given that the composition of EVs is similar to that of the cells that produced them.^[^ ^34^ ^]^ Seed‐derived factors promoted primary cancer progression by accelerating tumor growth, angiogenesis, and recruiting stromal cells. Thus, to eliminate the possibility of tumor‐promoting effects induced by “self‐seeded” tumor cells, we established an HCC‐EVs treated animal model rather than a bone lesion bearing animal model to explore the potential effects of BM‐EVs on orthotopic HCC progress. BM4 cells grew faster in bone (data not shown) and secreted more EVs compared to LM3 cells (Figure 1B). However, it is impossible to dynamically quantify the amount of LM3 EVs and BM4 EVs in the different stages of bone lesion‐bearing HCC mice. Thus, we chose to inject the same amount of LM3 EVs or BM4 EVs throughout the study. In fact, BM4 EVs may exert a greater prometastatic function in realistic pathophysiologic conditions. We demonstrated that BM‐EVs could promote lung metastasis in HCC. Meanwhile, it is arbitrary to assert that BM‐EVs fulfill this effect only by fueling the prometastatic phenotypes of orthotopic HCC cells. One notable possibility is that BM‐EVs settle into the lungs to prime metastatic niche formation.^[^ ^15^ ^]^ In fact, DiR‐ and PKH26‐stained BM‐EVs injected into mice were found in lung tissues (data not shown). Interestingly, we found that treatment with LM3 EVs and BM4 EVs did not give rise to the proliferation difference of HCC cells in vitro, while the orthotopic HCC cells in BM4 EVs‐injected mice displayer stronger proliferation ability that those treated with LM3 EVs. This discrepancy implies the local microenvironment may play a crucial role in the growth‐promoting effects of BM‐EVs, which deserves further investigation.

The role of EVs in organotropism has been identified and investigated.^[^ ^35^ ^]^ Exosomal integrins α6β4 and α6β1 predispose for lung metastasis, whereas exosomal integrin αvβ5 is linked to liver metastasis.^[^ ^36^ ^]^ In osteotropism, the enrichment of miRNA‐92a‐1‐5p in EVs can promote skeletal metastasis by disturbing bone homeostasis.^[^ ^37^ ^]^ Therefore, we speculated that the prometastatic phenotypes of HCC cells induced by BM‐EVs might exhibit a bone preference. However, in our study, only lung metastases were observed in HCC mice treated with BM‐EVs. We speculated that the short time interval, relatively low incidence of spontaneous bone metastasis of orthotopic HCC and limited sensitivity of BLI for bone metastatic foci may be blamed for the failure of bone metastasis detection. At the same time, although miR‐3190 was found to be upregulated in HCC‐BM cells and BM‐EVs, it promoted the EMT process and enhanced cell mobility, which is a nonspecific prometastatic transition. In addition, ISH and qRT‐PCR analyses showed that hepatic miR‐3190 or EV‐miR‐3190 in patients with HCC were upregulated in those with extrahepatic metastasis. These results further imply that the prometastatic effects of BM‐EVs are not organ‐specific. Similarly, Zhang et al. demonstrated that the enhanced metastatic abilities of bone‐entrained breast cancer cells are multiorgan rather than organotropism.^[^ ^4^ ^]^

ALKBH5 regulates cell cycle, autophagy, DNA repair, metabolism, immune response, and other cellular processes in cancer. The canonical effect of ALKBH5 is dependent on cooperating with m^6^A reader proteins and auxiliary ncRNAs.^[^ ^38^ ^]^ It was also reported that ALKBH5 interacted with HuR protein to increase its level. HuR promoted *EGFR* expression by regulating miR‐7, which activated prometastatic and antiautophagic signaling pathways. Through this m^6^A‐independent function cooperated with the m^6^A modification of *BCL2* mRNA, ALKBH5 promoted ovarian cancer progression.^[^ ^23^ ^]^ In this study, we found that ALKBH5 initiated a cluster of prometastatic genes in m^6^A‐dependent (DEPDC1 and NTSR1) and m^6^A‐independent (FN1, EGFR, CDH2, ITGA6 and ITGB4) manners. Similarly, Su et al. demonstrated that the “reader” of m^6^A, methyltransferase‐like protein 16 (METTL16) could deposit m^6^A into its target mRNA transcripts in the nucleus. Besides, METTL16 could also directly interact with the eukaryotic initiation factors 3a and ‐b as well as ribosomal RNA in the cytosol to exert functions independent of its m^6^A activity.^[^ ^31b^ ^]^ Therefore, together with previous studies, our study provided a new sight on the function models of m^6^A regulators.

Patients with HCC and bone metastasis exhibit worse outcomes than those without metastases.^[^ ^39^ ^]^ In our study, EVs secreted from bone lesions localized to orthotopic HCC and promoted new metastases from orthotopic HCC. We thus concluded that the existence of bone metastasis was a risk factor for secondary metastasis of HCC. Of note, bone metastasis of HCC is often imperceptible and is diagnosed only at postmortem examination.^[^ ^8^ ^]^ The prometastatic role of BM‐EVs revealed by this study emphasized the importance of monitoring and preventing the bone lesions formation for HCC patients. One interesting fact is that 71.6% of HCC bone‐metastasized patients had visceral metastasis.^[^ ^40^ ^]^ The metachronous or synchronous emergence of these lesions is debatable.^[^ ^41^ ^]^ The findings in our study support the hypothesis of metachronous metastasis.

There are still some limitations on this study. We did not provide direct clinical evidence that EVs of bone lesions can lead to further tumor metastasis and poorer prognosis in patients with HCC and bone metastasis. Clinical samples and preclinical animal models with temporal course, accompanied with high‐resolution techniques, are needed to unravel the mystery and landscape of multiorgan metastasis. Besides, we cannot rule out the influence of systemic changes induced by EVs treatment. Also, as mentioned above, the investigation of the involvement of local tumor microenvironment in BM‐EVs‐fueled HCC progression is absent. Moreover, liver has an immunosuppressive orientation, which impairs immune infiltration and immunotherapy efficacy in HCC.^[^ ^42^ ^]^ Ye et al. demonstrated that HCC cells secreted high mobility group box 1‐enriched exosomes to activate TIM‐1^+^ regulatory B cells expansion, which exhausted CD8^+^ T cells and induced an immunosuppressive microenvironment for HCC progression.^[^ ^43^ ^]^ However, under the existing conditions, we cannot explore whether immune factors are involved in BM‐EV‐absorbed tumor microenvironment. Therefore, it is necessary to establish immune totipotent bone‐ metastasized animal models to unveil the relevance between the immune microenvironment and BM‐EVs.

## 4 Experimental Section

### Patient Samples

Formalin‐fixed and paraffin‐embedded human HCC specimens (*n* = 110) were used for the in situ hybridization analysis of miR‐3190 expression (Table S1, Supporting Information). Snap‐frozen HCC samples (*n* = 67) were used to evaluate *ALKBH5* and miR‐3190 levels using qRT‐PCR. Plasma samples from 77 patients with HCC were collected to isolate EVs. All samples were collected from the Hepatic Surgery Center, Tongji Hospital, Huazhong University of Science and Technology (HUST, Wuhan, China). Approval was obtained from the Ethics Committee of Tongji Hospital of HUST, approval number: TJ‐IRB20210935. The study was conducted in accordance with the principles of the Declaration of Helsinki and Istanbul. Written informed consent was obtained from all the patients.

### Cell Lines and Cultures

The human HCC cell line HLF and human embryonic kidney cell line HEK‐293T were purchased from the China Center for Type Culture Collection (Wuhan, China). The HCC cell line, LM3, was obtained from the Liver Cancer Institute, Zhongshan Hospital, Fudan University (Shanghai, China). TheHCC‐BM cell lines, BM4 and HLF‐BM1 were isolated from skeletal metastatic lesions in mice in a previous study.^[^ ^6^ ^]^ All cell lines were cultured in Dulbecco's modified Eagle's medium (DMEM) (Hyclone, UT, USA) supplemented with 10% fetal bovine serum (Gibco, NY, USA) at 37 °C in a 5% CO_2_ incubator.

### Animal Experiments

Male BALB/c nude mice (4–5 weeks old) were purchased from HFK Bioscience Co. Ltd. (Beijing, China) and maintained under specific pathogen‐free conditions. All animal experiments were performed in accordance with the “The Animal Research: Reporting of In Vivo Experiments Guidelines 2.0” and were approved by the Committee on the Ethics of Animal Experiments of Tongji Hospital, approval number: S‐106‐20‐10‐0P.

For the liver orthotopic xenograft tumor model, 1 × 10^6^ luciferase‐bearing LM3 cells in 15 µL DMEM were mixed with 15 µL Matrigel (BD Biosciences, NJ, USA). Cell suspensions were inoculated into the left lobe of the livers of nude mice. After 14 d, EVs (50 µg in 100 µL) or antagomir/agomir‐loaded aptamer/liposome (A/Lipo) (5 nmol:390 pmol:195 µg in 100 µL) were injected via the tail vein every 3 d. After another 16 d, mouse blood was collected from the orbital vein before sacrifice. Tumor burdens in the liver and lung were detected by BLI using the SPECTRAL Lago X Imaging System (Spectral Instruments Imaging, Tucson, AZ, USA), and orthotopic tumor volume was calculated using the following formula: (length × width^2^)/2.

For the bone orthotopic xenograft tumor model, 1 × 10^6^ luciferase‐bearing Lck‐GFP cells were injected into the tibia through the middle of patellar ligament as previously described.^[^ ^44^ ^]^ PBS was injected as a blank control. For the HCC bone metastasis animal model, 1 × 10^6^ luciferase‐bearing HCC cells (LM3 and HLF) or bone‐metastasized cells (BM4 and HLF‐BM1) were injected into the left ventricle, as previously described.^[^ ^6^ ^]^ The bone tumor burden was periodically determined by BLI. At the end point, the excised hind legs were subjected to BLI examination and X‐ray analysis using the instrument mentioned above.

For the A/Lipo toxicity experiment, healthy nude mice were injected with A/Lipo every three days for five times and serum alanine aminotransferase and aspartate aminotransferase levels were evaluated by ServiceBio (Wuhan, China).

A part of excised mouse tissue was frozen by liquid nitrogen, and embedded by optimal cutting temperature compound (Sakura Finetek, Torrance, CA, USA). The remaining tissues were fixed in 4% paraformaldehyde for 48 h and embedded in paraffin. The bones were decalcified in 10% ethylenediaminetetraacetic acid for 2 weeks before being embedded in paraffin.

### Statistical Analyses

Statistical analyses were performed using SPSS 13.0 (SPSS, Armonk, NY, USA) and GraphPad Prism 9.0 (GraphPad, San Diego, CA, USA) software. Survival curves were constructed using the Kaplan–Meier method and compared between subgroups using the log‐rank test. Pearson's correlation analysis was used for measure strength of the association between two variables. Chi‐square test and Fisher's exact test were used to compare categorical variables. All other comparisons were analyzed using two‐tailed Student's *t*‐test. Results are reported as the mean ± standard error of mean. *P* < 0.05 was considered statistically significant, with * *P* < 0.05, ** *P* < 0.01, and *** *P* < 0.001. Details on Experimental Section are provided in the Supporting Information.

## Conflict of Interest

The authors declare no conflict of interest.

## Author Contributions

S.H., L.X., Y.W., and T.Y. contributed equally to this work. The author contribution is as follows: conception and design (S.H., L.G., Z.H., X.C., and B.Z.); acquisition of data (S.H., L.X., Y.W., T.Y., W.J., Y.Q., and Y.L.); analysis and interpretation of data (S.H. and Z.H.); drafting of the article (S.H. and Z.H.); administrative, technical, or material support (J.L., J.W., N.B., H.L., Q.L., Z.D., and X.Y.); and study supervision and funding acquisition (Z.H., X.C., and B.Z.).

## Supporting information

Supporting Information

## Data Availability

The data that support the findings of this study are available from the corresponding author upon reasonable request.
