# Supplementary material for: Bone Lesion‐Derived Extracellular Vesicles Fuel Prometastatic Cascades in Hepatocellular Carcinoma by Transferring ALKBH5‐Targeting miR‐3190‐5p
Source: Adv Sci (Weinh). 2023 Apr 25;10(17):2207080. doi: 10.1002/advs.202207080 (PMC10265039; doi:10.1002/advs.202207080)
Supplement: Supplementary file 1 — Supporting Information [file ADVS-10-2207080-s001.pdf]

ADVANCED SCIENCE
Open Access

Supporting Information

for *Adv. Sci.*, DOI 10.1002/advs.202207080

Bone Lesion-Derived Extracellular Vesicles Fuel Prometastatic Cascades in Hepatocellular Carcinoma by Transferring ALKBH5-Targeting miR-3190-5p

Shenqi Han, Lin Xue, Yi Wei, Tuying Yong, Wenlong Jia, Yongqiang Qi, Yiming Luo, Junnan Liang, Jingyuan Wen, Nana Bie, Huifang Liang, Qiumeng Liu, Zeyang Ding, Xiangliang Yang, Lu Gan, Zhao Huang*, Xiaoping Chen* and Bixiang Zhang**

Bone Lesion-Derived Extracellular Vesicles Fuel Pro-Metastatic Cascades in Hepatocellular Carcinoma by Transferring ALKBH5-Targeting miR-3190-5p

Shenqi Han, Lin Xue, Yi Wei, Tuying Yong, Wenlong Jia, Yongqiang Qi, Yiming Luo, Junnan Liang, Jingyuan Wen, Nana Bie, Huifang Liang, Qiumeng Liu, Zeyang Ding, Xiangliang Yang, Lu Gan, Zhao Huang*, Xiaoping Chen*, Bixiang Zhang**

Supplementary Materials and Methods

Extracellular vesicle (EV) isolation and characterization

To isolate EVs from culture medium, FBS was ultracentrifuged at 180,000×g for 6 h at 4 °C to remove EVs. The cells were cultured in DMEM supplemented with 10% EV-free FBS for 48 h. Supernatants were collected and centrifuged at 500×g for 10 min and at 12,000×g for 20 min at 4 °C to remove cell debris, followed by filtration through a 0.22 μm filter (Millipore, Tullagreen, Ireland) directly into a small ultracentrifuge tube. The EVs were pelleted by ultracentrifugation at 120,000×g for 70 min at 4 °C. Then the sediments were resuspended in 20 mL PBS (HyClone, Logan, UT, USA) and purified by ultracentrifugation at 120,000×g for 70 min at 4 °C. EVs were resuspended in PBS, and the total protein concentration was measured using a BCA protein assay kit (Thermo Fisher Scientific, Rockford, IL, USA).

To isolate EVs from plasma samples of patients, the samples were sequentially centrifuged at 500×g for 5 min and 2,000×g for 10 min and then filtered through a 0.22

μm filter to remove large particles. Ice-cold PBS was added to the ultracentrifuge tube to a total volume of 15 mL. The samples were ultracentrifuged at 100,000×g for 3 h at 4 °C. Next, the pellet was resuspended, washed in PBS, and ultracentrifuged again at 100,000×g for 3 h at 4 °C. Finally, the EV pellets were resuspended in PBS. All centrifugation was performed using an Optima XE-90 ultracentrifuge (Beckman Coulter, Brea, CA, USA) and Avanti JXN-26 centrifuge (Beckman Coulter).

The morphology of EV was detected and captured by transmission electron microscopy (TEM) (HITACHI, Tokyo, Japan), as described previously.[1] The size distribution of EV was measured by nanoparticle tracking analysis (NTA) using ZetaView (Particle Metrix, Meerbusch, Germany). EV biomarkers (Alix, TSG101, CD9, and CD63) and the exclusion marker (Calnexin) were detected in lysed EV proteins by western blotting, using the antibodies listed in Supplementary Table 7.

EV uptake assay

For the in vitro EVs uptake assay, the same amount of EVs (40 μg per cell sample) were stained with PKH26 (Sigma-Aldrich, Saint Louis, MO, USA) according to the manufacturer's protocol. Specifically, 250 μL Diluent C-loaded EVs were mixed with 250 μL Diluent C-loaded PKH26 Dye for 5 min at 20 °C. 500 μL 1% BSA (GENVIEW, FL, USA) was added to stop the staining. The labeled EVs were washed with 10 mL PBS, and collected by ultracentrifugation (120,000×g for 70 min). PKH26-labeled EVs were resuspended in DMEM supplemented with 10% EV-free FBS and cultured with cells for 24 h. Fluorescence in cells was observed using a ZEISS NOL-LSM 710 confocal laser scanning microscope (ZEISS, Oberkochen, Germany) and detected by

flow cytometry as described below.

For in vivo EV uptake assay, 1 mM DiR (KeyGEN, Nanjing, China) was used to stain equal amount of EVs (100 μg per mouse) with a final concentration of 5 μM at 37 °C for 30 min, according to the manufacturer's protocol. The EVs were washed in 10 mL PBS and ultracentrifuged to remove free DiR. Next, the DiR-labeled EVs were resuspended in PBS and injected into orthotopic HCC-bearing mice via the tail vein. After 24 h, the distribution of DiR-EVs in mice and liver tumors was analyzed using the SPECTRAL Lago X imaging system. In addition, PKH26-labeled EVs were also used for in vivo EV uptake. The tissues were frozen at -80 °C overnight and then embedded in optimal cutting temperature.

Flow cytometry analysis

Fresh mice tumor samples were cut into small pieces, single cell suspensions were prepared by digestion with DNase I and collagenase IV (Biosharp, Hefei, China), and filtration through a 70-μm cell strainers (Biosharp). PBS was used to remove cell debris and cellular aggregates. Red blood cells were lysed using ACK Lysis Buffer (Thermo Fisher). For cultured cells, equal amount of cells were collected and washed twice in PBS. Both mice tumor cells and cultured cell lines were resuspended in 200 μL of 1:1 prepared PBS and 4% paraformaldehyde (PFA). The cells were then subjected to a CytoFLEX Flow Cytometer (Beckman Coulter). The results were quantified using CytExpert software (version 2.4.0.28, Beckman Coulter) and the histograms were made by FlowJo X (version 10.0.7, BD Biosciences).

Immunofluorescent staining

Frozen slides were warmed at 20 °C for 10 min and rinsed twice with PBS. Subsequently, 50 mM ammonium chloride in PBS was applied to the slides to reduce the autofluorescence. The slides were then permeabilized with 0.5% Triton X-100 (Solarbio, Beijing, China) in PBS for 20 min and blocked with 5% BSA in PBS for 30 min. Primary antibody against AFP was used to incubate sections overnight at 4 °C. The next day, the sections were washed three times with PBS and then incubated with Alexa Fluor 488-conjugated anti-rabbit IgG for 1 h at 20 °C. After washing with PBS, the slides were stained with DAPI (Servicebio, Wuhan, China) for 5 min at 20 °C. Images were acquired using a ZEISS NOL-LSM 710 confocal laser scanning microscope or KEYENCE BZ-X810 fluorescence microscope (Osaka, Japan). Antibody information is presented in Supplementary Table 7.

Transwell assays

Cells (2×10^4 cells for migration assays or 4×10^4 cells for invasion assays) were incubated with EVs (40 μg/mL) or transfected with RNAs or plasmids for 48 h. Cell migration and invasion assays were conducted using 8 μm pore size 24-well Transwell plates (Corning, New York, USA). For cell invasion assays, the upper chamber was pre-coated with 50 μL 20% Matrigel (BD Biosciences) for 2 h at 37 °C. Cells were seeded into the upper chamber in 200 μL of FBS-free DMEM, and 600 μL of DMEM containing 10% FBS was added to the lower chambers. After 24 h, cells on the upper side were scraped off, and the plates were fixed in 4% PFA, followed by crystal violet staining. Three random fields in each chamber were captured with a liquid crystal digital microscope (IDM2300, Jiangnan, Nanjing, China), and the cells were counted

using ImageJ software (version 1.52a, National Institute of Health, USA).

For EV treatment, 40 μg EVs were added with proteinase K (Vazyme Biotech, Nanjing, China) at a final concentration of 10 μg/mL for every well, and incubated on ice for 30 min. RNase A (Solarbio) was added to a final concentration of 50 U/mL, and catalyzed for 20 min at 30 °C. Additionally, these treatments were performed in the presence or absence of 0.01% Triton X-100. Cells were co-cultured with these EVs for 48 h at 37°C prior to transwell assays. The data are shown as mean ± SEM of three independent experiments.

Wound healing assay

Cells were incubated with EVs (40 μg/mL) or transfected with RNAs or plasmids for 48 h. The same number of cells in FBS-free DMEM for each group was seeded into plates. The confluent monolayer cells were scratched using pipette tips. Three random fields in each wound area were imaged by phase-contrast microscopy (Life Technologies, Carlsbad, CA, USA) at 0 h and 48 h after the scratch. The wound healing rate (%) was calculated as the migrated cell wound area/total wound area × 100. The data are shown as mean ± SEM of three independent experiments.

Cell Counting Kit 8 (CCK8) assay

Cells (2×10^3/well) were treated with HCC-EVs or BM-EVs at a concentration of 40 μg/mL for 48 h, and seeded into 96-well plates with 5 replicates for each group. PBS treatment was set as blank control. At the indicated time points, cells were incubated using CCK8 (Beyotime Institute of Biotechnology, Shanghai, China) for 1 h at 37 °C according to the manufacturer's instructions. Optical density (OD) was measured at 450

nm using an enzyme-linked immunosorbent assay plate reader (Bio-Tek, Winooski, VT, USA). The average gross OD value was calculated after subtracting the OD value of the blank control. The data are shown as mean ± SEM of three independent experiments.

Western blotting

Western blotting was performed as described previously.[2] Antibody information is presented in Supplementary Table 7. GAPDH was used as a loading control. Peroxidase AffiniPure goat anti-rabbit or mouse IgG (111-035-003/115-035-003; Jackson ImmunoResearch, West Grove, PA, USA) was purchased from Promoter (Wuhan, China). The membrane was exposed to the ChemiDoc MP Imaging System (Bio-Rad, Hercules, CA, USA), the image density of the immunoblotting was determined using the Image Lab software (version 5.2.1, Bio-Rad), and the western blotting bands were quantified using ImageJ software.

F-actin staining

Cells in 20 mm glass bottom cell culture dish (NEST, Wuxi, China) were fixed with 4% PFA for 20 min, followed by permeabilization with 0.1% Triton X-100 for 5 min. Subsequently, 100 μL Phalloidin-iFluor™ 594 conjugate solution (1:1000 in PBS containing 1% BSA; AAT Bioquest, Pleasanton, CA, USA) was added to each well and incubated for 90 min at 20 °C. After washing with PBS for 3 times, the nuclei were stained with DAPI for 5 min. Images were captured using a ZEISS NOL-LSM 710 confocal laser scanning microscope.

RNA isolation and quantitative real-time PCR (qRT-PCR)

Total RNA was extracted from cells using RNAiso Plus (Takara, Kusatsu, Shiga,

Japan), and miRNAs in EVs were isolated using the MiPure Cell/Tissue miRNA Kit (Vazyme).

cDNAs were reverse-transcribed from mRNAs using the HiScript II 1st Strand cDNA Synthesis Kit (Vazyme). For miRNA reverse transcription, we used the Mir-X miRNA First-Strand Synthesis Kit (Takara). ChamQ Universal SYBR qPCR Master Mix (Vazyme Biotech) was used for qRT-PCR. The indicated samples were treated with EVs in the presence of 10 μM 5,6-dichlorobenzimidazole riboside (MCE, Shanghai, China) or 10 μM dynasore (MCE) before RNA extraction and qRT-PCR. Relative RNA expression was calculated using the $2^{-\Delta\Delta Ct}$ method, with normalization to *GAPDH* or *U6*. The primers used are listed in Supplementary Table 8. The data are shown as mean ± SEM of three independent experiments.

Plasmid, lentivirus construction and cell transfection

The coding sequence (CDS) and 3' untranslated region (3'UTR) of *ALKBH5* were cloned into the pcDNA3.1-3×Flag (#182494, Addgene, Cambridge, MA, USA) plasmid for transient overexpression. The ALKBH5 (H204A) plasmid was constructed by site-specific mutagenesis. pcDNA3.1-ALKBH5/-ALKBH5 (H204A) plasmids, miR-3190 mimic, inhibitor, and ALKBH5 siRNA (RiboBio, Guangzhou, China) were individually mixed with Lipofectamine 3000 transfection reagent (Invitrogen, Waltham, MA, USA) in Opti-MEM (Gibco, Grand Island, NY, USA) according to the manufacturer's instructions. The transfection reaction was then added to the cell culture medium.

The CDS and 3'UTR of *ALKBH5* was cloned into the pLenti-CMV-GFP-Puro

plasmid (#17448, Addgene), and the shRNA sequence of *ALKBH5* or *RAB27A* was respectively cloned into the pLKO.1-puro (#8453, Addgene) or pLKO.1-blast (#26655, Addgene) vector. The constructed plasmids and Lck-GFP expression plasmid (#61099, Addgene) were co-transfected with pMD2.G (#12259, Addgene) and psPAX2 (#12260, Addgene) to generate lentiviruses in HEK-293T cells. The viral supernatant was collected 48 h after transfection and filtered through a 0.45 μm filter (Millipore). To establish stable cell lines, lentivirus was added to HCC cells with Polybrene (10 μg/mL) (Sigma-Aldrich) and screened using 10 μg/mL puromycin (Solarbio) or 50 μg/mL blasticidin (InvivoGen, Hong Kong, China). miR-3190-overexpression and miR-3190-knockdown lentivirus were purchased from GENECHEM (Shanghai, China). The miRNA mimic, inhibitor, siRNAs, and shRNA sequences are listed in Supplementary Table 8.

Isolation of bone- or lung-educated cells from mice

Bone- or lung-educated HCC cells were isolated from mice tibia with bone lesions or mice lung with tumor burdens. In brief, mice were sacrificed to extract bone or lung tumor tissues. The tumor tissues were cut into small pieces and digested with collagenase IV at 37 °C for 20 min. Single cell suspensions were collected through cell strainers, and centrifuged at 300×g for 5 min. Pellets were resuspended in fresh DMEM supplemented with 10% FBS and 1× Penicillin/Streptomycin (meilunbio, Dalian, China). Eventually, the cells were purified with 400 μg/mL geneticin (Solarbio).

EV-miR-3190 transfer detection

BM4 and HLF-BM1 cells were transfected with Cy3-miR-3190 mimic (RiboBio)

for 48 h. EVs were isolated and added to the culture medium (40 μg/mL) of LM3 or HLF cells for 24 h. The cells were then washed with PBS and fixed with 4% PFA. The nuclei were stained with DAPI for 5 min. The fluorescent signals in the HCC cells were detected using a KEYENCE BZ-X810 fluorescence microscope.

Luciferase reporter assay

The wild-type (WT) and corresponding mutant (MT) miR-3190 binding sites within the 3'UTR sequence of *ALKBH5* were cloned into the psiCHECK^{TM} -2 vector (C8021, Promega, Madison, WI, USA). Cells were seeded at a density of 6×10^4 cells/well in 24-well plates, followed by co-transfection with 100 ng of WT or MT ALKBH5 reporter plasmids and 50 nM miR-3190 mimic (mi-3190) using Lipofectamine 3000 transfection reagent (Invitrogen). Then, 48 h later, cells were collected for luciferase assay using the Dual-Luciferase® Reporter 1000 Assay System (Promega) with a GloMax 20/20 Luminometer (Promega). Relative luciferase activity was calculated by normalizing Renilla luciferase intensity to that of Firefly. The sequences of the 5 WT sites and their corresponding mutations are shown in Supplementary Table 4. The data are shown as mean ± SEM of three independent experiments.

In situ hybridization and immunohistochemistry analyses

miR-3190 expression in human and mouse HCC tissues was assessed by ISH. The specific oligo probe (5'Digoxin-TGGGGACGTAGCTGGCCAGA-3') for miR-3190 and miRNA ISH kit were produced by Boster Biological Technology (Pleasanton, CA, USA); the ISH assay was performed according to the manufacturer's instructions.

Sections of Formalin-fixed and paraffin-embedded animal samples were subjected to IHC staining as described previously.[3] The antibodies used for IHC are listed in Supplementary Table 7. The ISH and IHC staining were scored according to the staining intensity and percentage of positively stained tumor cells as described previously.[3] The scoring rules for the staining intensity were as follows: 0 point (negative), 1 point (light brown), 2 points (brown), 3 points (dark brown). The scoring rules for the percentage of positively stained tumor cells were as follows: 0 point (less than 10%), 1 point (10-25%), 2 points (26-50%), 3 points (51-75%) and 4 points (more than 75%). ISH and IHC staining scores were evaluated by three independent pathologists. ISH and IHC sections were scanned using Pannoramic MIDI (3DHISTECH, Budapest, Hungary).

miRNA microarray analysis

This experiment was performed by the OE Biotech Corporation (Shanghai, China). Briefly, total RNA was quantified using a NanoDrop ND-2000 (Thermo Fisher) and RNA integrity was assessed using an Agilent Bioanalyzer 2100 (Agilent Technologies, Santa Clara, CA, USA). After dephosphorylation of the samples, they were labeled with Cy3-pCp and hybridized onto the microarray using the miRNA Complete Labeling and Hyb Kit (Agilent Technologies). Finally, the microarray was washed using the Gene Expression Wash Pack (Agilent Technologies) and scanned using an Agilent Scanner G2505C (Agilent Technologies). Feature Extraction software (version10.7.1.1, Agilent Technologies) was used to extract raw data from the microarray, which were normalized for further analyses.

Methylated RNA immunoprecipitation sequencing and RNA sequencing

Total RNAs was extracted using the TRIzol reagent (Invitrogen). RNA quality and quantity were determined by examining A260/A280 with a Nanodrop™ OneCspectrophotometer (Thermo Fisher) and Qubit3.0, with the Qubit™ RNA Broad Range Assay kit (Life Technologies). Fifty micrograms of total RNAs were used for polyadenylated RNA enrichment using VAHTS mRNA capture beads (Vazyme). RNA fragments of 100–200 nt were incubated with an m^6A-specific antibody (202203, Synaptic Systems, Gottingen, Germany) for m^6A immunoprecipitation. The stranded RNA sequencing library was constructed using the KC-Digital™ Stranded mRNA Library Prep Kit for Illumina® (Seqhealth, Wuhan, China) following the manufacturer's instructions and sequenced on a Novaseq 6000 sequencer (Illumina, San Diego, CA, USA) with the PE150 model. For RNA-seq, 150–200 ng of input mRNA from each sample was used to generate the library using the Illumina kit.

Filtered MeRIP-seq data were used for m^6A site analysis. ExomePeak (version 3.8) software was used for peak calling, deepTools (version 2.4.1) was used for peak distribution analysis, and differentiated m^6A peaks were identified using a Python script. Sequence motifs enriched in m^6A peak regions were verified using Homer (version 4.10). For RNA-seq analysis, reads mapped to the exon regions of each gene were counted using featureCounts (Subread-1.5.1, Bioconductor) following RPKM value calculation. Analysis of differentially expressed genes and other analyses were performed using R 4.0.5 (R Foundation for Statistical Computing, Vienna, Austria). KEGG enrichment analysis was performed using KOBAS software (version 2.1.1).

RNA immunoprecipitation (RIP) and methylated RNA immunoprecipitation (MeRIP)

RIP and MeRIP were performed using the Megna RIP RNA-binding Protein Immunoprecipitation Kit (Millipore) and the Magna MeRIP m^6A Assay (Millipore), respectively following the manufacturer's instructions. The relative co-precipitated RNA levels were calculated as the Ct values of the IP portion divided by the Ct values of the input portion (IP/input), and finally normalized to the IgG group. The antibodies and primers used for RIP and MeRIP are listed in Supplementary Tables 7 and 8.

In silico prediction

The target genes of miR-3190 were predicted using four bioinformatics tools: TargetScan,[4] miRTarbase,[5] miRDB,[6] and mirDIP.[7] Binding sites between miR-3190 and *ALKBH5* 3'UTR were predicted using TargetScan.

Gene set enrichment analysis (GSEA)

For gene set enrichment analysis (GSEA), genes were ranked in descending order based on their log-fold change in expression between LM3/sh-NC cells and LM3/sh-ALKBH5 cells. The pre-ranked gene list was then run against the selected gene set collections obtained from the GSEA MSigDB database[8] using the clusterProfiler R package.[9] 10,000 permutations were used to compute enrichment *P*-values for each pathway, and pathways with *P*-value < 0.1 were considered to be significantly enriched.

m^6A dot blot assay

m^6A dot blot assay was performed as previously described.[10] Total RNA was isolated using RNAiso Plus (Takara) and diluted to 500 ng/μL using a NanoDrop ND-

2000 (Thermo Fisher). RNA was added onto a Hybond-N+ membrane (GE Healthcare, Chicago, IL, USA) and crosslinked twice in a CL-1000 UV Crosslinker (UVP, Upland, CA, USA). The PBST wash buffer was prepared with 1×PBS and 0.02% Tween-20 (Dingguo, Beijing, China). Blocking buffer and antibody dilution buffer were prepared with 5% BSA in PBST. Information on the anti-m^{6}A antibodies is listed in Supplementary Table 7. The membrane was exposed to the ChemiDoc MP Imaging System and analyzed using the Image Lab software.

A/Lipo preparation and characterization

For antagomir/agomir-loaded aptamer/liposome system preparation, the HCC-specific aptamer TLS11a was mixed with lipid in absolute ethanol (SCR, Co., Ltd, Shanghai, China) and then added to 250 μL antagomir/agomir-citrate buffer accompanied by vortex oscillation for 10 s. The construction of A/Lipo was verified using TEM and NTA, as described above. A fluorescence spectrophotometer (PTI, Santa Clara, CA, USA) was used to measure the emission wavelength of the FAM-aptamer. The uptake of A/Lipo was assessed using flow cytometry, as described above. Representative images of A/Lipo uptake were photographed using a ZEISS NOL-LSM 710 confocal laser scanning microscope. The antagomir/agomir-3190 and the aptamer were synthesized by Qijing Biological Technology (Wuhan, China). The miRNA antagomir and agomir sequences are listed in Supplementary Table 8.

References

[1] S. García-Silva, A. Benito-Martín, L. Nogués, A. Hernández-Barranco, M. S.

Mazariegos, V. Santos, M. Hergueta-Redondo, P. Ximénez-Embún, R. P. Kataru, A. A. Lopez, C. Merino, S. Sánchez-Redondo, O. Graña-Castro, I. Matei, J. Á. Nicolás-Avila, R. Torres-Ruiz, S. Rodríguez-Perales, L. Martínez, M. Pérez-Martínez, G. Mata, A. Szumera-Ciećkiewicz, I. Kalinowska, A. Saltari, J. M. Martínez-Gómez, S. A. Hogan, H. U. Saragovi, S. Ortega, C. Garcia-Martin, J. Boskovic, M. P. Levesque, P. Rutkowski, A. Hidalgo, J. Muñoz, D. Megías, B. J. Mehrara, D. Lyden, H. Peinado, *Nature cancer* **2021**, *2* (12), 1387, https://doi.org/10.1038/s43018-021-00272-y.

[2] Z.-y. Ding, G.-n. Jin, W. Wang, W.-x. Chen, Y.-h. Wu, X. Ai, L. Chen, W.-g. Zhang, H.-f. Liang, A. Laurence, M.-z. Zhang, P. K. Datta, B. Zhang, X.-p. Chen, *Hepatology (Baltimore, Md.)* **2014**, *60* (5), 1620, https://doi.org/10.1002/hep.27273.

[3] Z. Liao, L. Chen, X. Zhang, H. Zhang, X. Tan, K. Dong, X. Lu, H. Zhu, Q. Liu, Z. Zhang, Z. Ding, W. Dong, P. Zhu, L. Chu, H. Liang, P. K. Datta, B. Zhang, X. Chen, *Hepatology (Baltimore, Md.)* **2020**, *72* (3), https://doi.org/10.1002/hep.31104.

[4] B. P. Lewis, C. B. Burge, D. P. Bartel, *Cell* **2005**, *120* (1), 15.

[5] S.-D. Hsu, F.-M. Lin, W.-Y. Wu, C. Liang, W.-C. Huang, W.-L. Chan, W.-T. Tsai, G.-Z. Chen, C.-J. Lee, C.-M. Chiu, C.-H. Chien, M.-C. Wu, C.-Y. Huang, A.-P. Tsou, H.-D. Huang, *Nucleic Acids Res.* **2010**, *39* (suppl_1), D163, https://doi.org/10.1093/nar/gkq1107 %J Nucleic Acids Research.

[6] Y. Chen, X. Wang, *Nucleic Acids Res.* **2019**, *48* (D1), D127, https://doi.org/10.1093/nar/gkz757 %J Nucleic Acids Research.

[7] T. Tokar, C. Pastrello, A. E. M. Rossos, M. Abovsky, A.-C. Hauschild, M. Tsay, R. Lu, I. Jurisica, *Nucleic Acids Res.* **2017**, *46* (D1), D360,

https://doi.org/10.1093/nar/gkx1144 %J Nucleic Acids Research.

[8] A. Liberzon, C. Birger, H. Thorvaldsdóttir, M. Ghandi, J. P. Mesirov, P. Tamayo, *#N/A* **2015**, *1* (6), 417.

[9] T. Wu, E. Hu, S. Xu, M. Chen, P. Guo, Z. Dai, T. Feng, L. Zhou, W. Tang, L. Zhan, X. Fu, S. Liu, X. Bo, G. Yu, *Innovation (Cambridge (Mass.))* **2021**, *2* (3), 100141, https://doi.org/10.1016/j.xinn.2021.100141.

[10] L. Shen, Z. Liang, H. Yu, *Bio-protocol* **2017**, *7* (1), e2095, https://doi.org/10.21769/BioProtoc.2095.

Supplementary Figures and Figure Legends

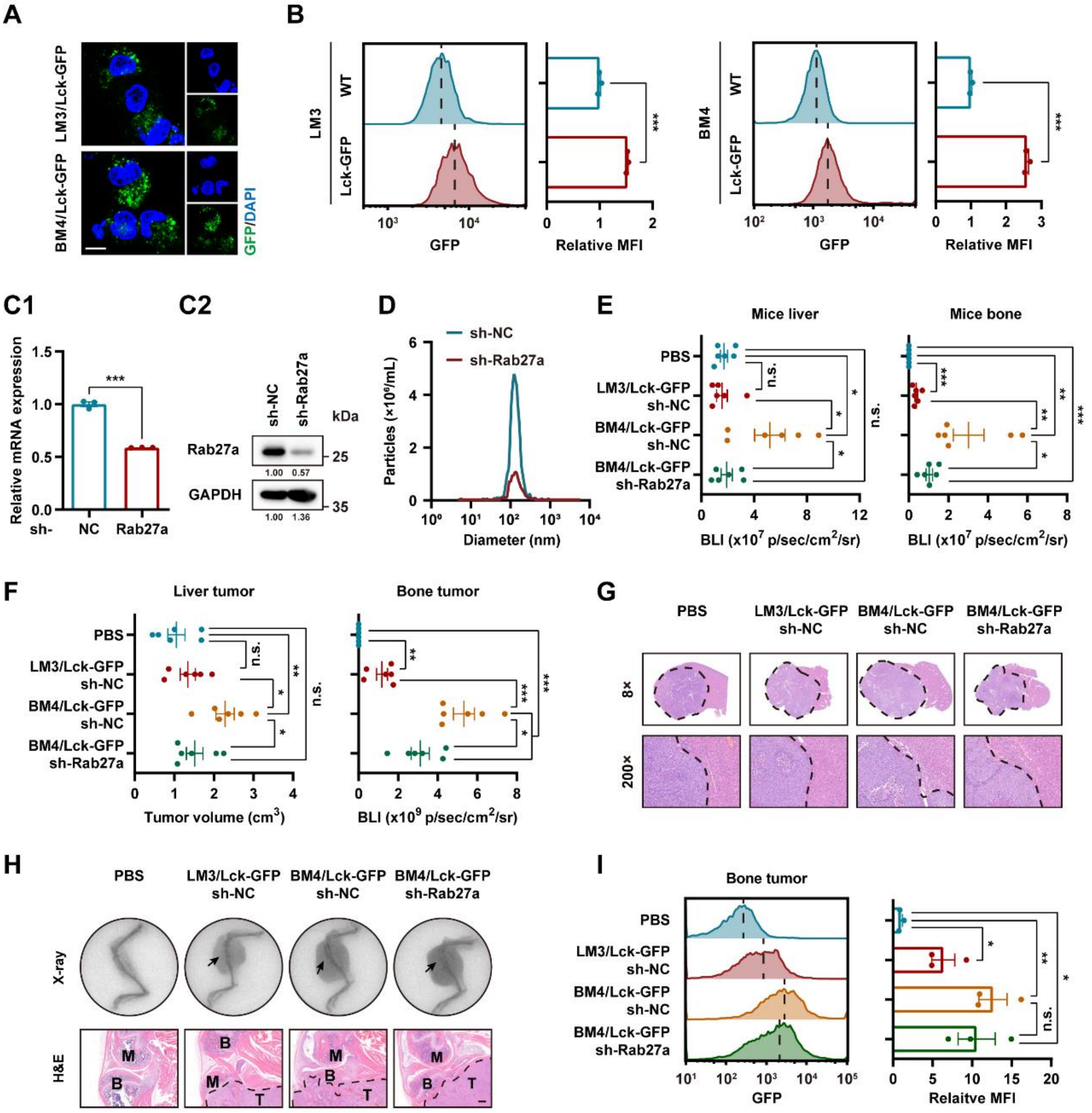

Figure S1. Characterization of GFP-labeled EVs and HCC animal model bearing bone lesions. (A and B) LM3 cells were treated with EVs isolated from the indicated cells for 24 h. Representative confocal images (A) and flow cytometry analysis (n=3) (B) of GFP signaling in LM3 cells. Blue, DAPI; green, GFP. Scale bar, 10 μm in (A). Data were normalized to their representative WT-EV-treated group in (B). (C and D) BM4/Lck-GFP cells were transduced with lentivirus carrying the *RAB27A* shRNA sequence (sh-Rab27a). qRT-PCR (C1) and western blot analyses (C2) of *RAB27A*

knockdown efficacy in BM4/Lck-GFP cells. Data are shown as fold change relative to negative control cells in (C1). GAPDH as loading control in (C2). (D) NTA of EVs derived from BM4/Lck-GFP cells with *RAB27A* knockdown or not. (E–I) Mice liver and bone samples in Figure 1D were subjected to quantification, H&E staining, X-ray and flow cytometry analyses. (E) BLI quantification of mice tumor burden in Figure 1E. (F) Tumor volume and BLI quantification of mice liver tumor and bone tumor in Figure 1F. (G) Representative H&E staining of the LM3 liver tumor in the indicated groups. (H) Representative X-ray images and H&E staining of bone lesions in the indicated groups. Scale bar, 500 μm. (I) Flow cytometry analysis of GFP signaling in the indicated bone tumor lesions (n=3). Data were normalized to the PBS group. Data are shown as mean ± SEM. *$P < 0.05$, **$P < 0.01$, ***$P < 0.001$, Student's *t* test. WT: wild-type; BM4, LM3-BM4; NC, negative control; sh, small hairpin RNA; B, bone; M, bone marrow; T, tumor. MFI, mean fluorescence intensity; n.s., no significance.

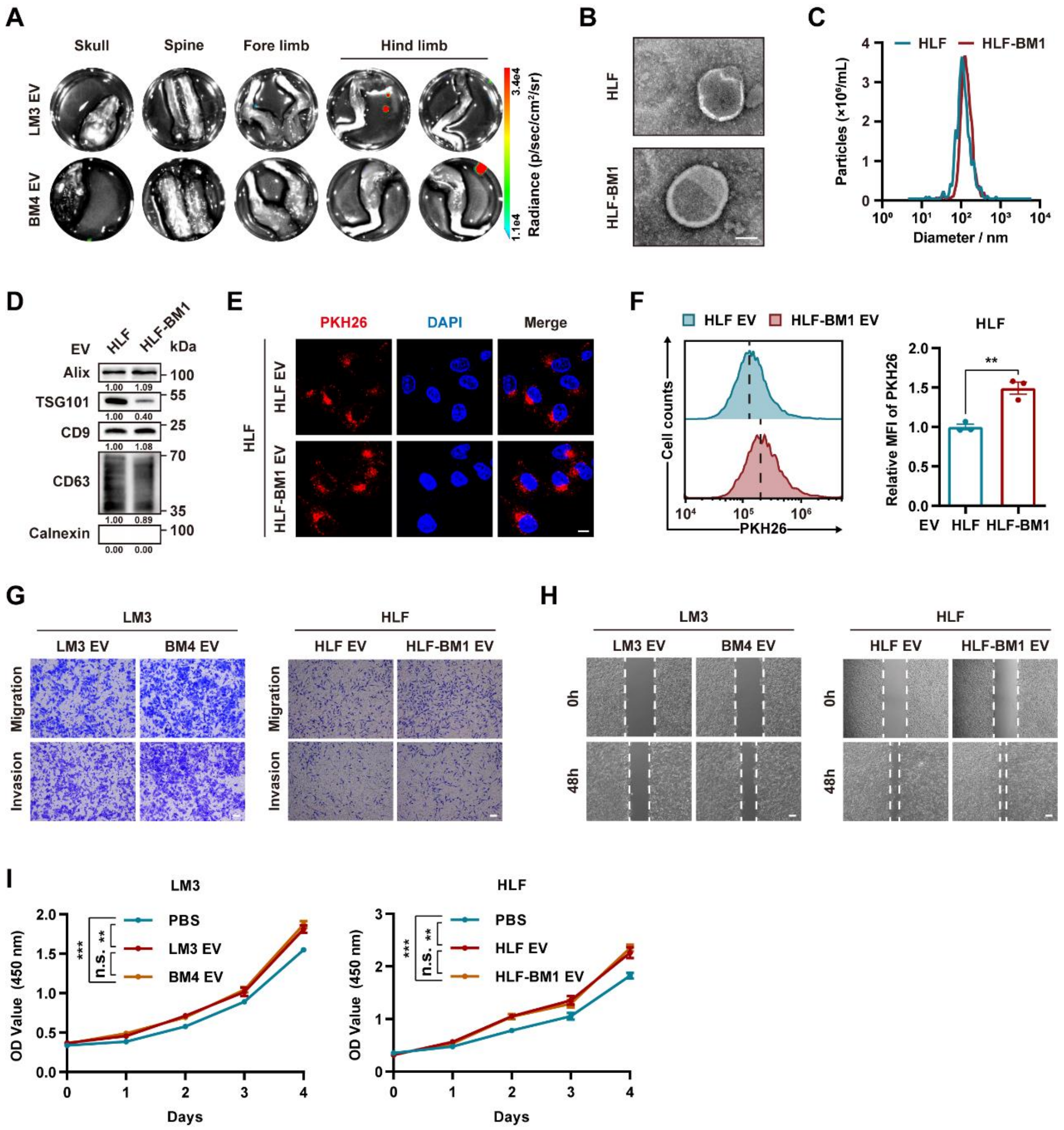

Figure S2. EVs derived from bone-metastasized HCC cells promote HCC cells mobility. (A) Representative BLI images of excised bones in mice treated with the indicated EVs. (B–D) Characterization of EVs isolated from the HLF and its bone-metastasized progeny HLF-BM1 cells by TEM, NTA, and western blot analyses. Scale bar, 100 μm in (B). (E and F) HLF cells were treated with the indicated EVs stained with PKH26 for 24 h. Confocal images (E) and flow cytometry analysis (n=3) (F) of fluorescent signaling intensity in HLF cells. Blue, DAPI; red, PKH26. Scale bar, 10 μm in (E). Data were normalized to the HLF EV-treated group in (F). (G–I) LM3 and HLF

cells were treated with the indicated EVs for 48 h. Representative images of cell migration and invasion assays (G), and wound healing assay (H). Scale bar, 25 μm. (I) CCK8 assays for the indicated cells treated with EVs, PBS was treated as blank control (n=5). Data are shown as mean ± SEM. $^{**}P < 0.01$, $^{***}P < 0.001$, Student's t test. OD, optical density.

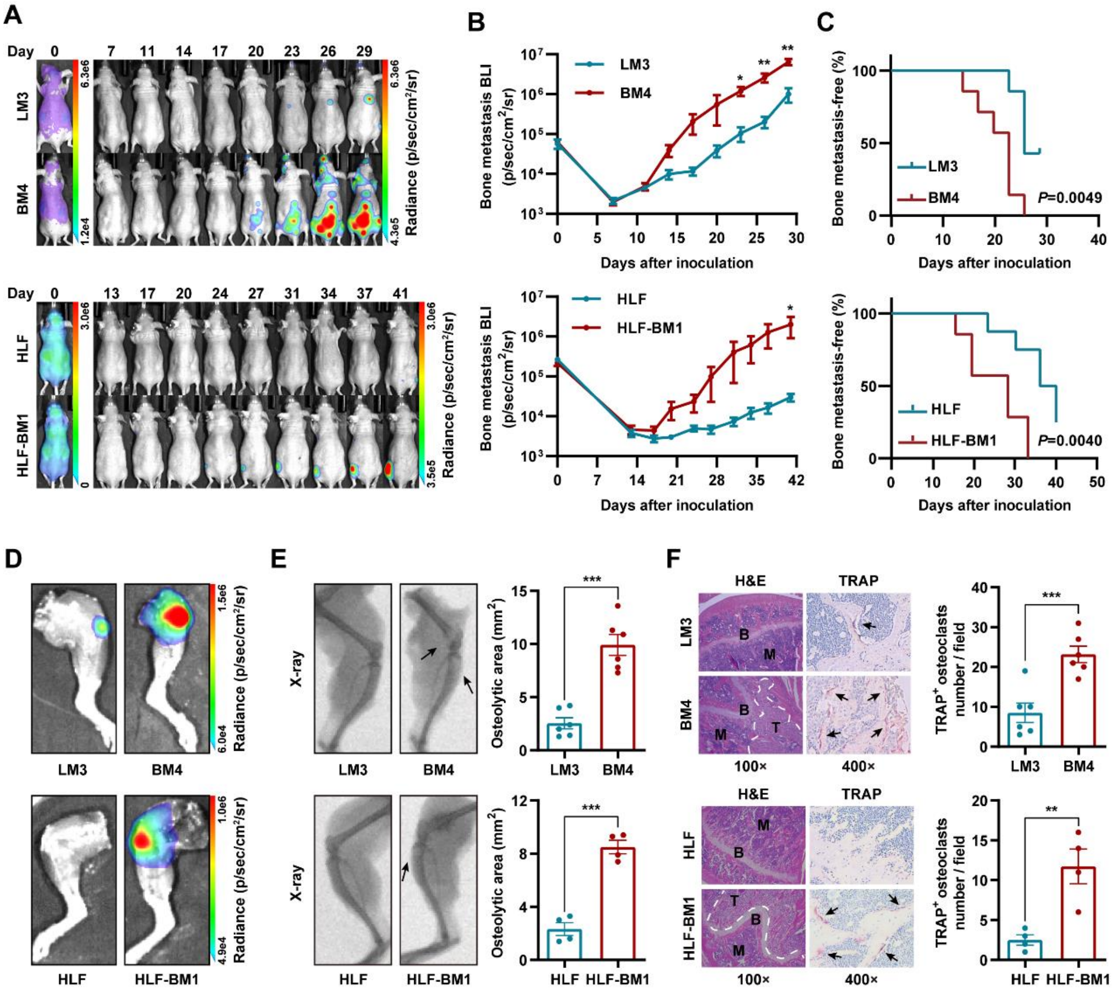

Figure S3. Bone metastatic capacities of HCC-BM cell lines. Mice were intracardially injected with BM4, HLF-BM1, and their respective ancestor HCC cells, LM3 and HLF. (A) Representative BLI intensities of bone metastatic lesions in mice. n=7 in LM3, BM4, and HLF-BM1 groups, n=8 in HLF group. (B) Quantification of

BLI signals in the indicated bone lesions of mice. (C) Kaplan–Meier analysis of bone metastasis-free survival in the indicated groups. (D) Representative images of bone metastasis by postmortem BLI examination. (E) Representative images and quantification of bone destruction indicated by X-ray assays. (F) H&E and TRAP staining of bone lesions and quantification of $TRAP^+$ osteoclasts. Black arrows, osteoclasts. n=6 in LM3 and BM4 groups, n=4 in HLF and HLF-BM1groups in (E and F). Data are shown as mean ± SEM. *$P < 0.05$, **$P < 0.01$, ***$P < 0.001$, Student's *t* test in (B, E and F), log-rank test in (C). B, bone; M, bone marrow; T, tumor.

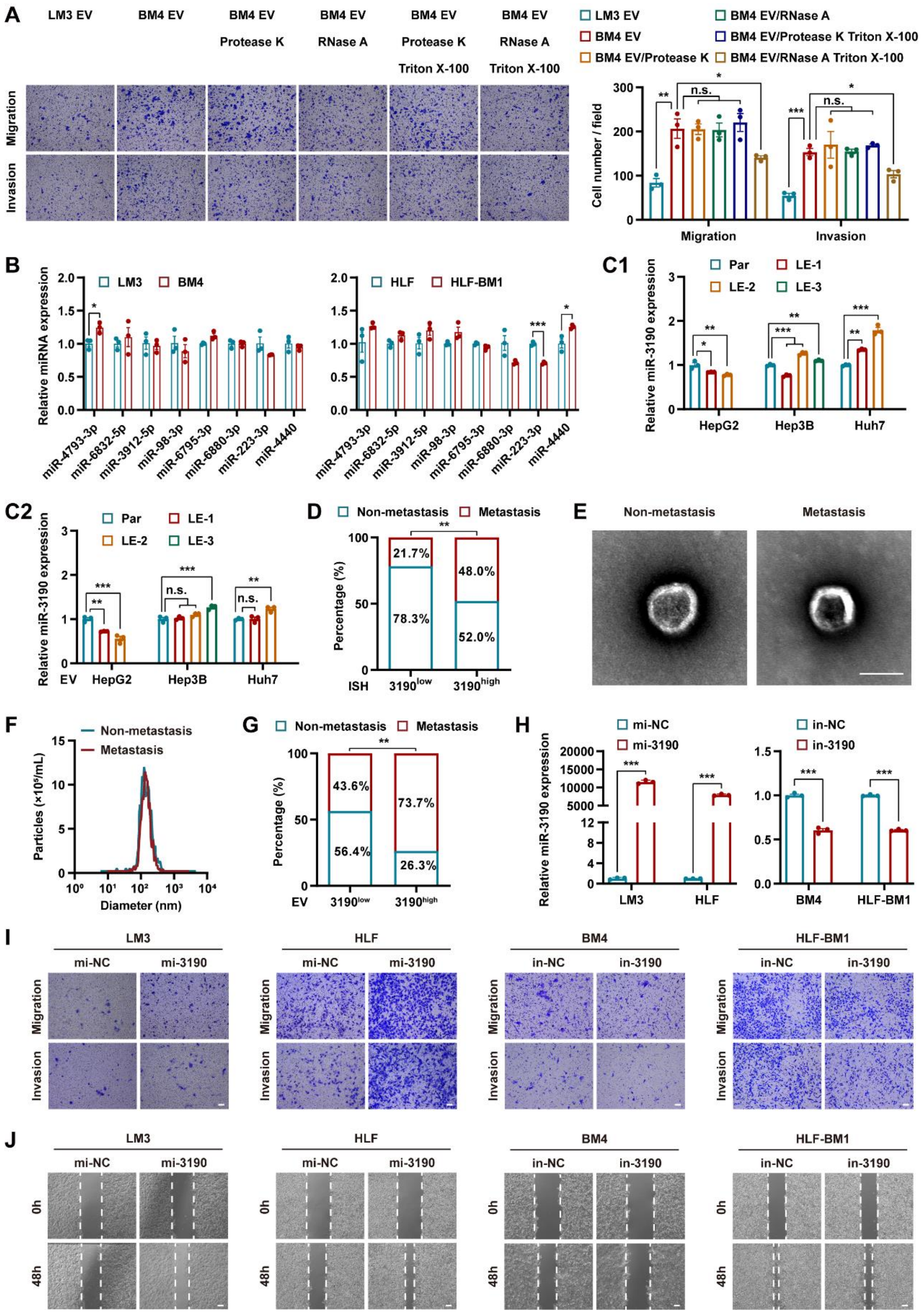

Figure S4. miR-3190 plays a pro-metastatic role in HCC. (A) LM3 cells were co-cultured with RNase A- or proteinase K-treated EVs with or without Triton X-100. Representative images and quantification analysis of migrated and invaded LM3 cells

in the indicated groups. (B) qRT-PCR analysis of the indicated miRNAs in BM4, HLF-BM1, and their respective parental HCC cells, LM3 and HLF. Data are shown as fold change relative to their respective parental cells. (C) The expression of miR-3190 was examined in cells and EVs of parent HCC (Par) cell lines (HepG2, Hep3B, and Huh7) and their respective lung-educated (LE) progenies. Data are shown as fold change relative to the parent cells or EVs. (D) Chi-square analysis of the correlation between metastasis with miR-3190 level of in situ HCC specimens from patients. n=60 in miR-3190^{low} group, n=50 in miR-3190^{high} group. (E and F) Representative images of TEM and NTA of plasma EVs in patients with metastasized HCC (n=45) or not (n=32). Scale bar, 100 nm in (E). (G) Chi-square analysis of the correlation between metastasis with miR-3190 level of plasma EVs in patients with HCC. (H–J) HCC cells were transfected with miR-3190 mimic (mi-3190) or inhibitor (in-3190) for 48 h. (H) qRT-PCR analysis of miR-3190 overexpression and knockdown efficacy in HCC cells. Data are shown as fold change relative to their respective negative control cells (mi-NC or in-NC). (I) Representative images of migrating and invading cells. Scale bar, 25 μm. (J) Representative images of wound healing assay. Scale bar, 25 μm. Data are shown as mean ± SEM. **P* < 0.05, ***P* < 0.01, ****P* < 0.001, Student's *t* test in (A, B, C and H), chi-square test in (D and G). mi, mimic; in, inhibitor.

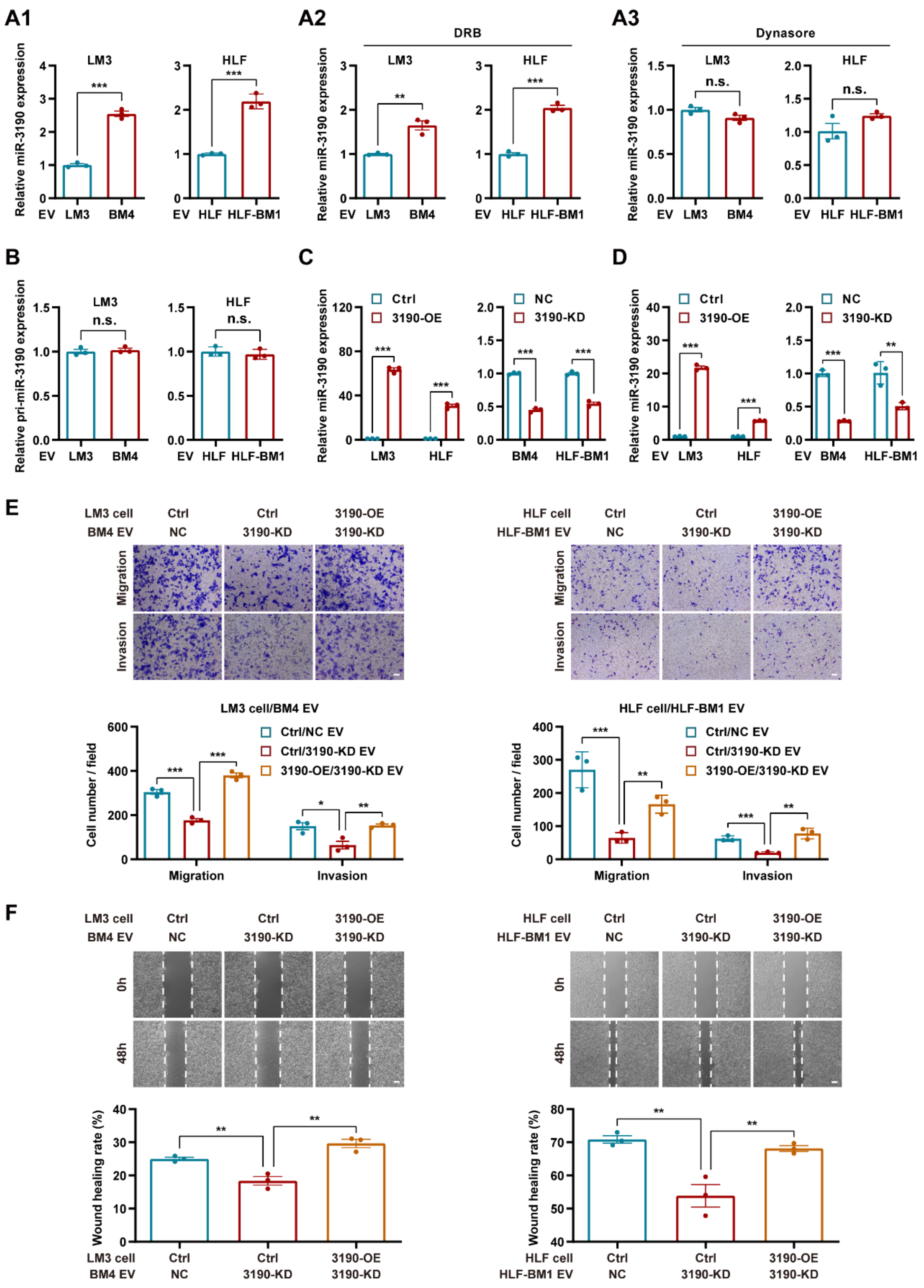

Figure S5. miR-3190 mediated the pro-metastatic role of BM-EVs. (A) qRT-PCR analysis of miR-3190 in LM3 and HLF cells treated with the indicated EVs for 24 h, accompanied with a RNA transcription inhibitor, DRB, or an inhibitor of endocytic

pathways, Dynasore. (B) qRT-PCR analysis of primary-miR-3190 in LM3 and HLF cells treated with the indicated EVs for 24 h. Data are shown as fold change relative to HCC cells treated with LM3 or HLF EVs in (A and B). (C and D) BM4, HLF-BM1, and their respective ancestor HCC cells, LM3 and HLF cells were transduced with lentivirus to stably overexpressing (3190-OE) or knocking down miR-3190 (3190-KD). qRT-PCR analysis of miR-3190 in cells (C) and their respective EVs (D). Data are shown as fold change relative to their respective negative control cells or EVs (Ctrl or NC). (E and F) EVs isolated from BM4 and HLF-BM1 cells with or without miR-3190 knocked down (3190-KD or NC) were used to treat LM3 and HLF cells with or without miR-3190 overexpression (3190-OE or Ctrl) for 48 h. (E) Representative images and quantification of migrating and invading cells. Scale bar, 25 μm. (F) Representative images and quantification of wound healing assay. Scale bar, 25 μm. Data are shown as mean ± SEM. *P < 0.05, **P < 0.01, ***P < 0.001, Student's t test. DRB, 5,6-dichlorobenzimidazole riboside; Ctrl, control; OE, overexpression; NC, negative control; KD, knockdown.

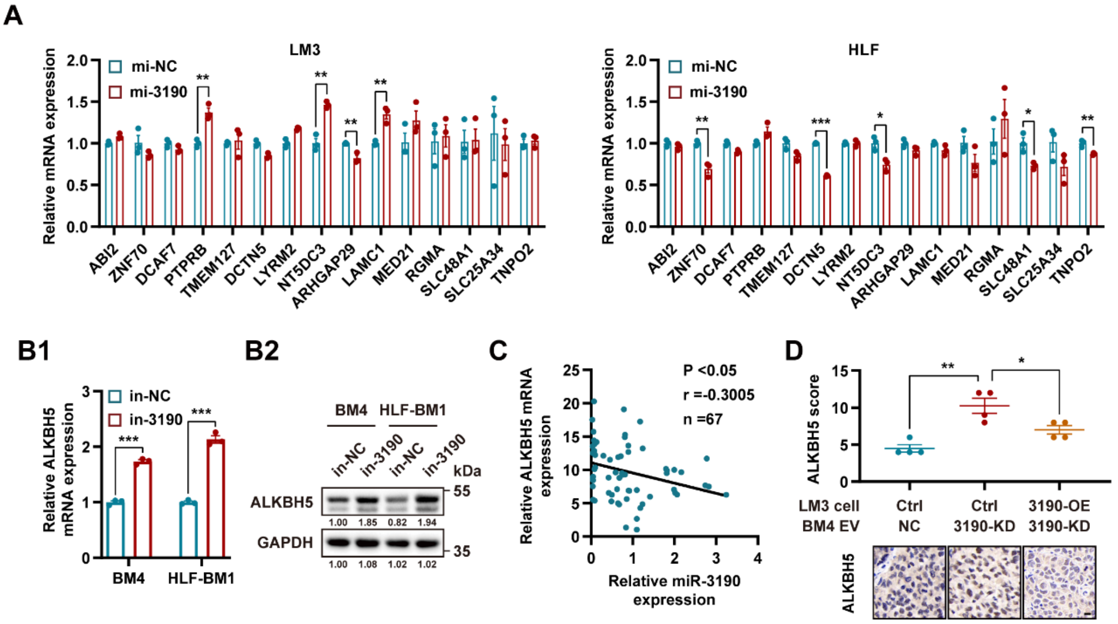

Figure S6. ALKBH5 is the downstream target of miR-3190. (A) qRT-PCR analysis of the indicated genes in LM3 and HLF cells transfected with miR-3190 mimic (mi-3190) for 48 h. Data are shown as fold change relative to HCC cells transfected with negative control mimic (mi-NC). (B) HCC cells were transfected with miR-3190 inhibitor (in-3190) for 48 h. qRT-PCR (B1) and western blot (B2) analyses of *ALKBH5* in the indicated cells. Data are shown as fold change relative to HCC cells transfected with negative control inhibitor (in-NC) in (B1). GAPDH as loading control in (B2). (C) qRT-PCR analysis of correlation between relative *ALKBH5* mRNA level with miR-3190 in HCC specimens. (D) Representative images and quantification of ALKBH5 IHC staining in indicated liver tumor slides in Figure 4B (n=4). Scale bar, 10 μm. Data are shown as mean ± SEM. **P* < 0.05, ***P* < 0.01, ****P* < 0.001, Student's *t* test in (A, B1 and D), Pearson's correlation analysis in (C).

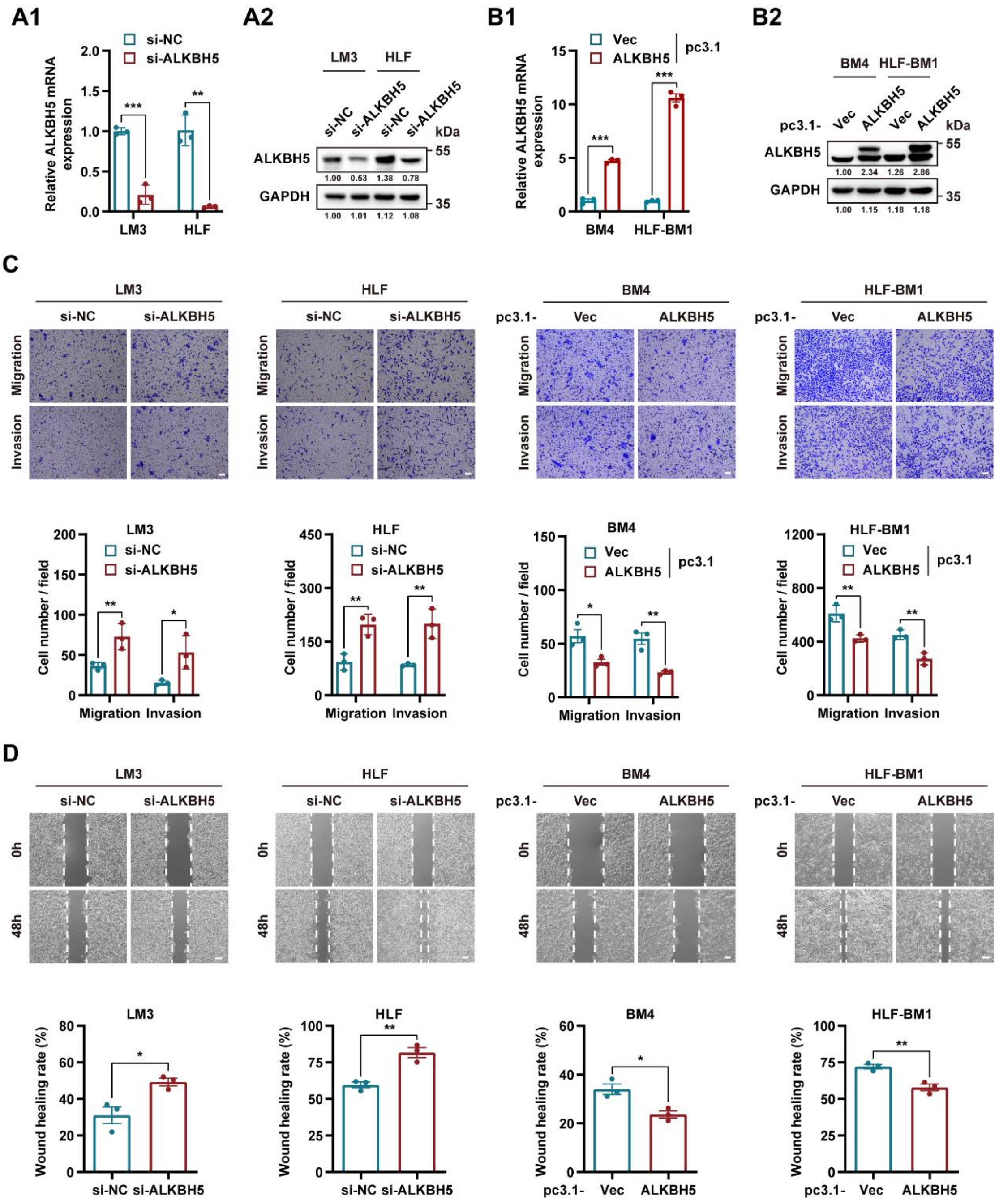

Figure S7. ALKBH5 suppresses mobility of HCC cells. (A) HCC cells were transfected with siRNA targeting *ALKBH5* (si-ALKBH5) for 48 h. qRT-PCR (A1) and western blot analyses (A2) of *ALKBH5* in the indicated cells. Data are shown as fold change relative to HCC cells transfected with negative control siRNA (si-NC) in (A1). (B) HCC cells were transfected with pcDNA3.1-ALKBH5 plasmid (pc3.1-ALKBH5) for 48 h. qRT-PCR (B1) and western blot (B2) analyses of *ALKBH5* in the indicated

cells. Data are shown as fold change relative to HCC cells transfected with vector plasmids (pc3.1-Vec) in (B1). (C) Representative images and quantification of migrating and invading cells in HCC cells treated as in (A) and in (B). Scale bar, 25 μm. (D) Representative images and quantification of wound healing rates in HCC cells treated as in (A) and in (B). Scale bar, 25 μm. GAPDH as loading control in (A2 and B2). Data are shown as mean ± SEM. *$P < 0.05$, **$P < 0.01$, ***$P < 0.001$, Student's *t* test. si, small interference RNA; pc3.1, pcDNA3.1; Vec, vector.

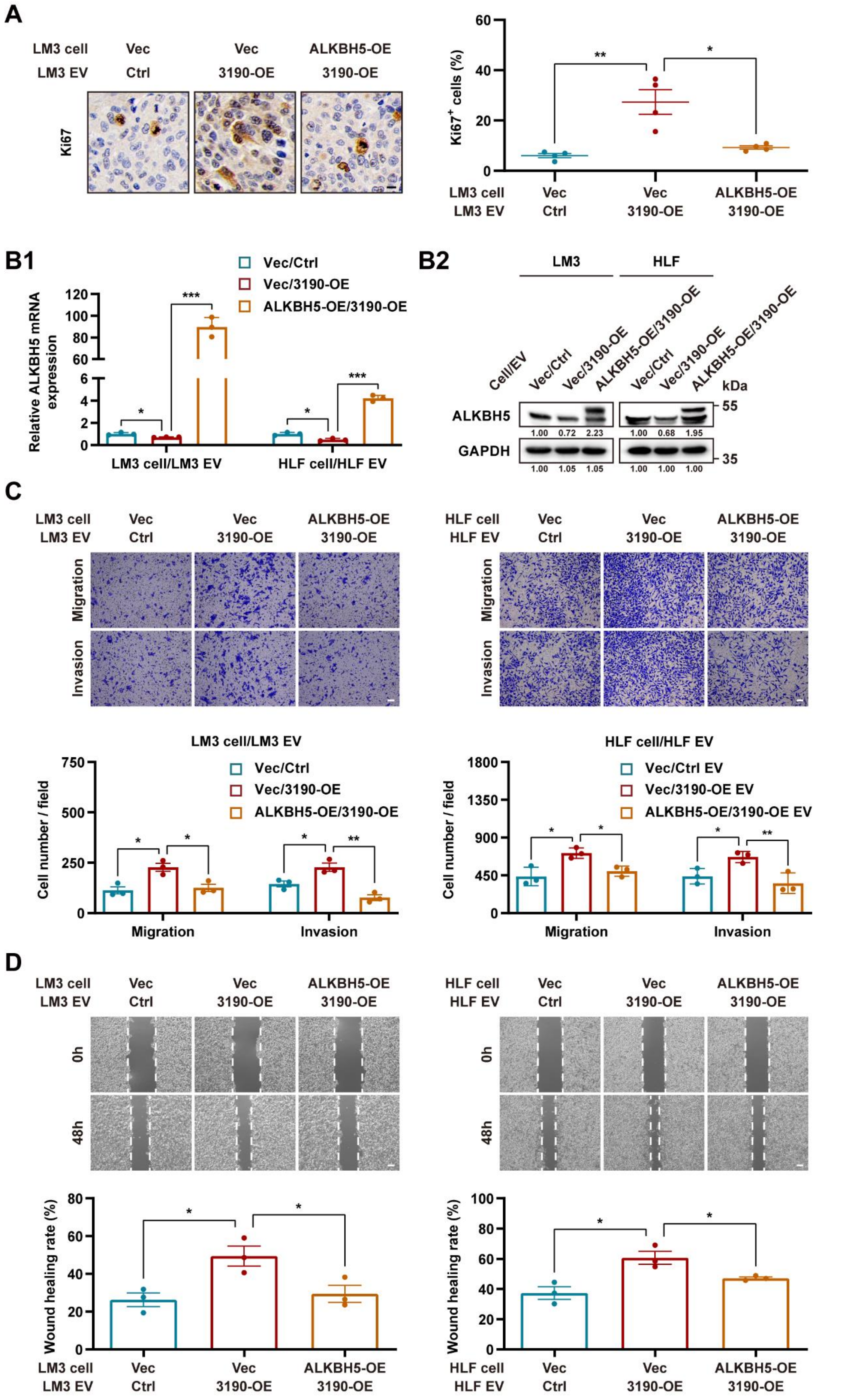
A
LM3 cell Vec Vec ALKBH5-OE
LM3 EV Ctrl 3190-OE 3190-OE
Ki67
Ki67+ cells (%)
B1
Vec/Ctrl
Vec/3190-OE
ALKBH5-OE/3190-OE
Relative ALKBH5 mRNA expression
LM3 cell/LM3 EV
HLF cell/HLF EV
B2
LM3 HLF
Cell/EV Vec/Ctrl Vec/3190-OE ALKBH5-OE/3190-OE Vec/Ctrl Vec/3190-OE ALKBH5-OE/3190-OE
kDa
ALKBH5
1.00 0.72 2.23 1.00 0.68 1.95
GAPDH
1.00 1.05 1.05 1.00 1.00 1.00
55
35
C
Migration
Invasion
LM3 cell/LM3 EV
HLF cell/HLF EV
Cell number / field
Vec/Ctrl EV
Vec/3190-OE EV
ALKBH5-OE/3190-OE EV
D
0h
48h
Wound healing rate (%)
HLF cell Vec Vec ALKBH5-OE
HLF EV Ctrl 3190-OE 3190-OE

Figure S8. ALKBH5 mediates the pro-metastatic role of miR-3190-enriched EVs. (A) Representative images and quantification of Ki67 IHC staining in HCC tumor sections in Figure 5E (n=4). Scale bar, 10 μm. (B–D) EVs isolated from HCC cells with or without miR-3190 overexpression (3190-OE or Ctrl) were used to treat LM3 and HLF cells with or without *ALKBH5* overexpression (ALKBH5-OE or Vec) for 48 h. qRT-PCR (B1) and western blot (B2) analyses of *ALKBH5* expression in the indicated cells. Data are shown as fold change relative to the negative control group in (B1). GAPDH as loading control in (B2). (C) Representative images and quantification of migrating and invading cells. Scale bar, 25 μm. (D) Representative images and quantification of wound healing rates. Scale bar, 25 μm. Data are shown as mean ± SEM. *$P < 0.05$, **$P < 0.01$, ***$P < 0.001$, Student's *t* test.

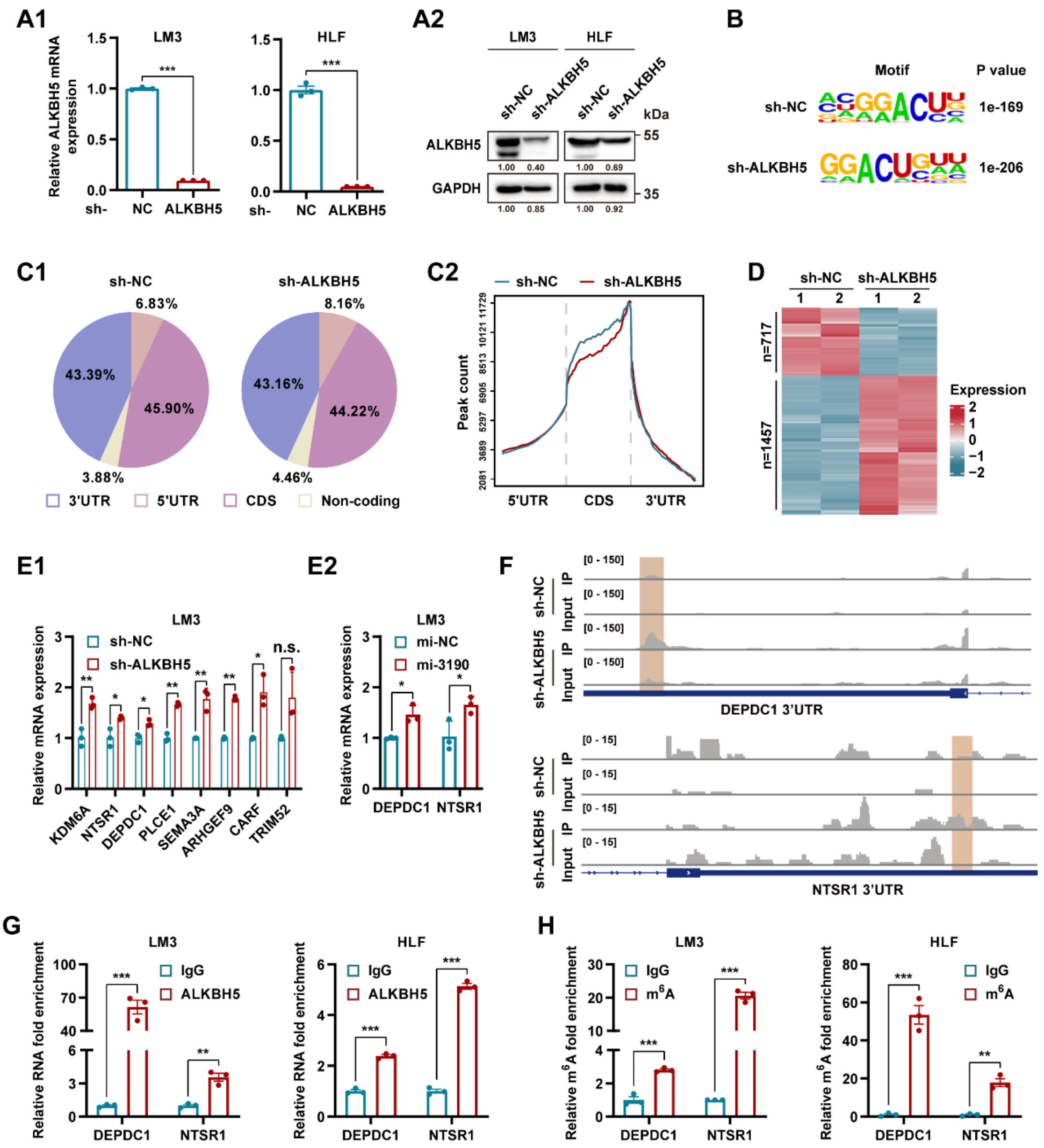

Figure S9. Validation of m^{6}A-dependent downstream effectors of ALKBH5. (A) LM3 and HLF cells were transduced with lentivirus carrying the *ALKBH5* shRNA sequence (sh-ALKBH5). qRT-PCR (A1) and western blot (A2) analyses of *ALKBH5* knockdown efficacy. Data are shown as fold change relative to the negative control group in (A1). GAPDH as loading control in (A2). (B) Representative m^{6}A motifs in the indicated groups. (C) Characterizations of m^{6}A modification sites in RNA. (C1) Percentages of m^{6}A modification in different regions of RNA. (C2) Distribution of m^{6}A

peaks in transcripts. (D) Heatmap of differentially expressed genes in LM3/sh-NC and LM3/sh-ALKBH5 cells. (E) qRT-PCR analysis of the indicated genes in HCC cells with *ALKBH5* knockdown (sh-ALKBH5) (E1) or miR-3190 overexpression (mi-3190) (E2). Data are shown as fold change relative to negative control cells (sh-NC or mi-NC). (F) Abundance of MeRIP IP and Input m^{6}A peaks along *DEPDC1* and *NTSR1* mRNA in LM3 sh-NC and sh-ALKBH5 cells. The m^{6}A sites modified by ALKBH5 are highlighted in 3'UTR. (G and H) qRT-PCR analysis of the indicated mRNA enrichment in anti-ALKBH5 (G) or anti-m^{6}A (H) immunoprecipitants. Data are shown as fold change relative to anti-IgG group. Data are shown as mean ± SEM. *$P < 0.05$, **$P < 0.01$, ***$P < 0.001$, Student's t test.

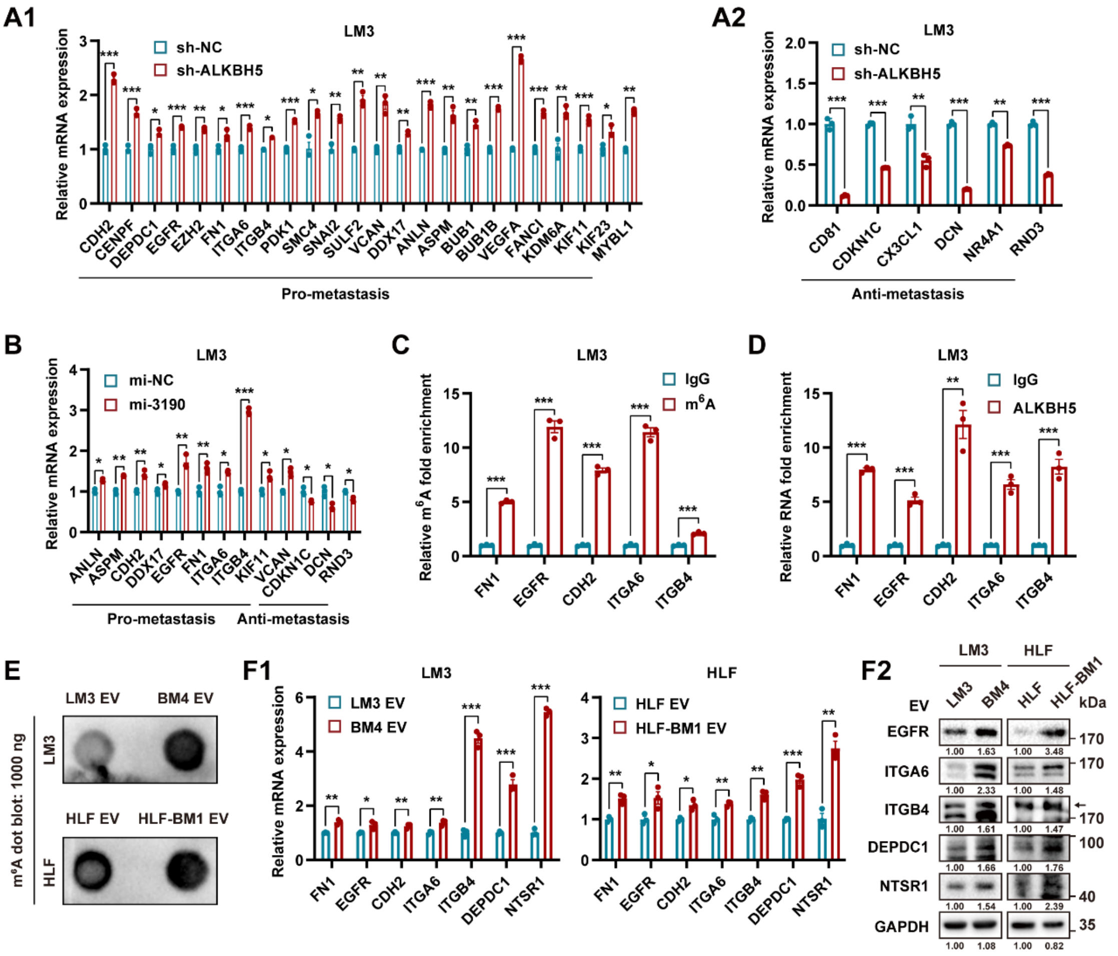

Figure S10. Validation of m^6A-independent downstream effectors of ALKBH5. (A and B) qRT-PCR analysis of the indicated genes with significant differences in HCC cells with *ALKBH5* knockdown (sh-ALKBH5) (A) or miR-3190 overexpression (mi-3190) (B). Data are shown as fold change relative to negative control cells (sh-NC or mi-NC). (C and D) qRT-PCR analysis of the indicated mRNA enrichment in anti-m^6A (C) or anti-ALKBH5 (D) immunoprecipitants. Data are shown as fold change relative to anti-IgG group. (E and F) HCC cells were subjected to the indicated EVs treatment for 48 h. (E) Global m^6A level of RNA extracted from the indicated HCC cells was measured via m^6A dot blot assay. (F) qRT-PCR (F1) and western blot (F2) analyses of the indicated genes. Data are presented as fold change relative to cells treated with LM3 or HLF EVs in (F1). GAPDH as loading control in (F2). Data are shown as mean ± SEM. *$P < 0.05$, **$P < 0.01$, ***$P < 0.001$, Student's *t* test.

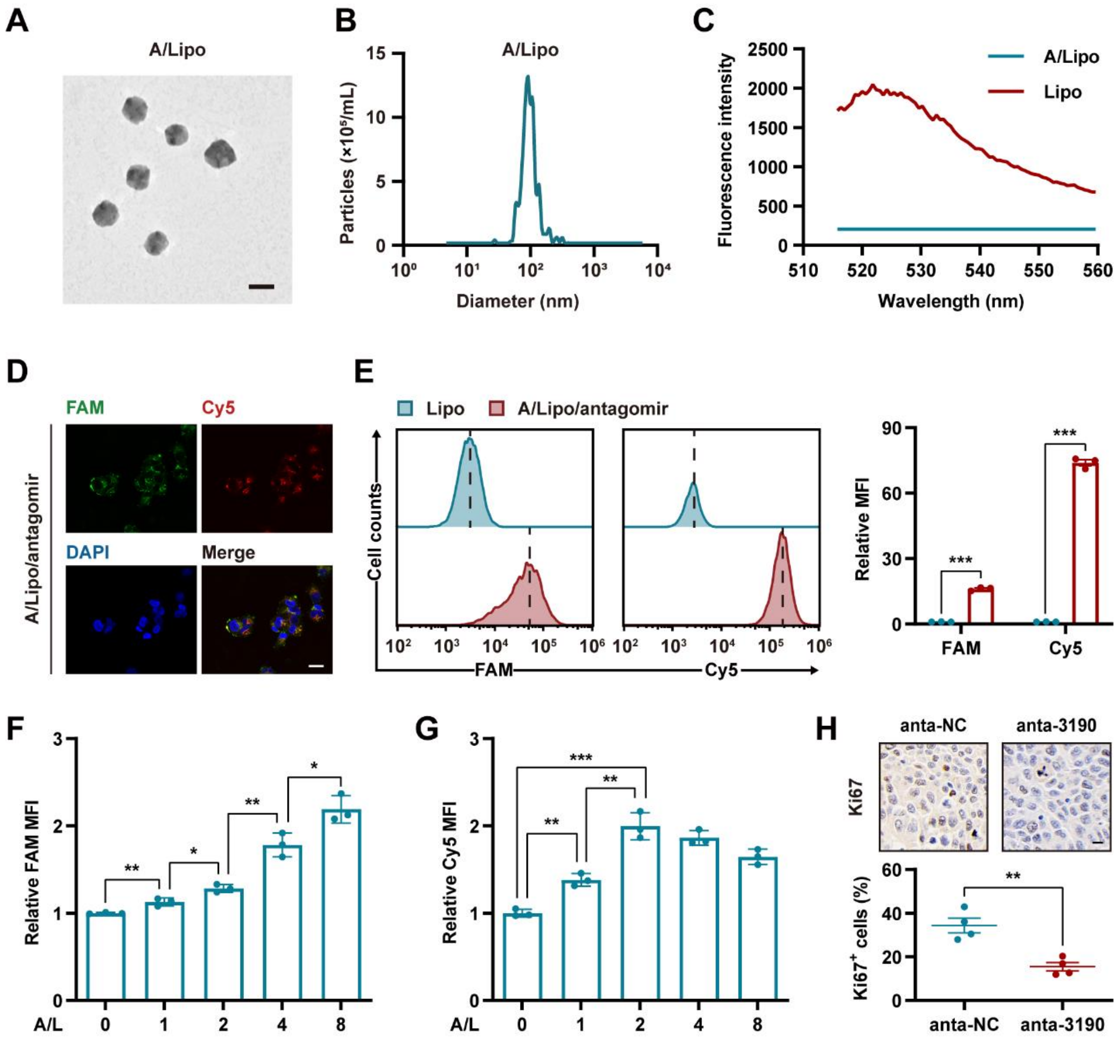

Figure S11. Characterization of aptamer/liposome complex and optimization of aptamer/liposome/antagomir system. (A) Representative TME image of aptamer/liposome (A/Lipo) complex. Scale bar, 100 nm. (B) Quantification of the size and amount of A/Lipo complex by NTA. (C) Fluorescence intensity and range of liposome attached with (A/Lipo) or without (Lipo) FAM-labeled aptamer. (D and E) Confocal images (D) and flow cytometry analysis (n=3) (E) of fluorescent signaling intensity of LM3 cells treated with FAM-aptamer/liposome/Cy5-antagomir-3190 for 24 h. Blue, DAPI; green, FAM; red, Cy5. Scale bar, 20 µm in (D). Data are shown as fold change compared to their negative control cells treated with liposome alone in (E). (F and G) Flow cytometry analysis of FAM (F) and Cy5 (G) intensity in LM3 cells treated

with FAM-aptamer/liposome/Cy5-antagomir-3190 with different ratios of aptamer with liposome for 24h (n=3). (H) Representative images and quantification of Ki67 IHC staining in HCC tumor sections in Figure 7E (n=4). Scale bar, 10 μm. Data are shown as mean ± SEM. **P* < 0.05, ***P* < 0.01, ****P* < 0.001, Student's *t* test. A/L, aptamer (pmol): liposome (μg).

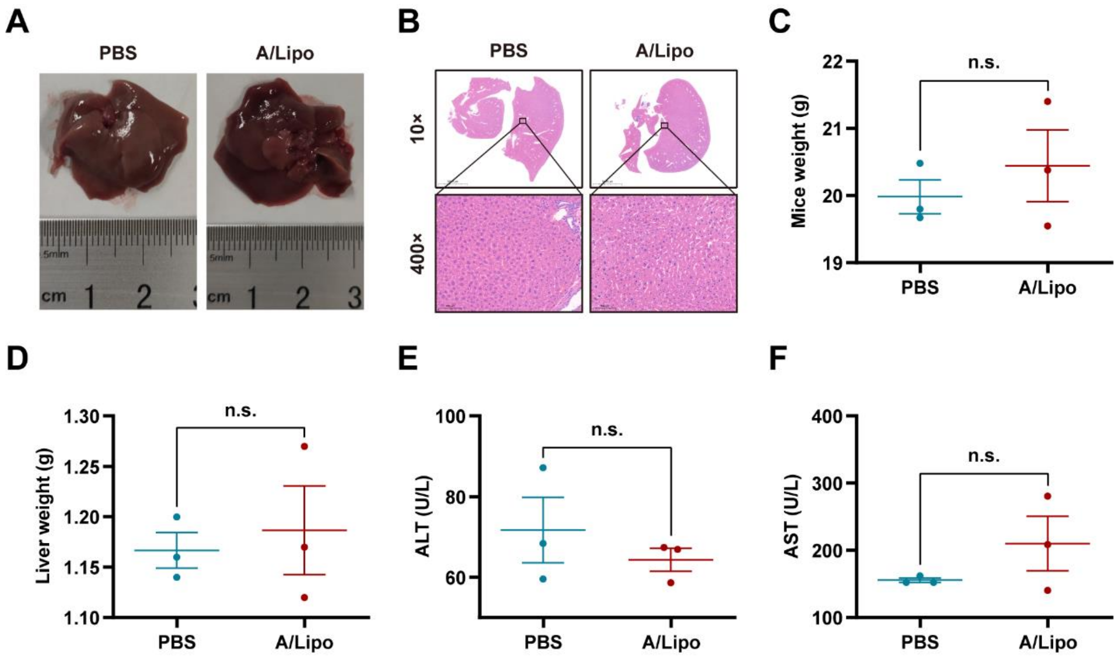

Figure S12. Evaluation of liver toxicity using A/Lipo system in vivo. Healthy mice were treated with A/Lipo or PBS with the same amount and interval as in Figure 7E (n=3). (A) Representative macroscopic images of excised liver tissues. (B) Representative images of H&E staining of liver. (C and D) Quantification of mice (C) and liver (D) weight. (E and F) Quantification of ALT (E) and AST (F) level in mice plasma. Data are shown as mean ± SEM, Student's *t* test. ALT, alanine aminotransferase; AST, aspartate aminotransferase.

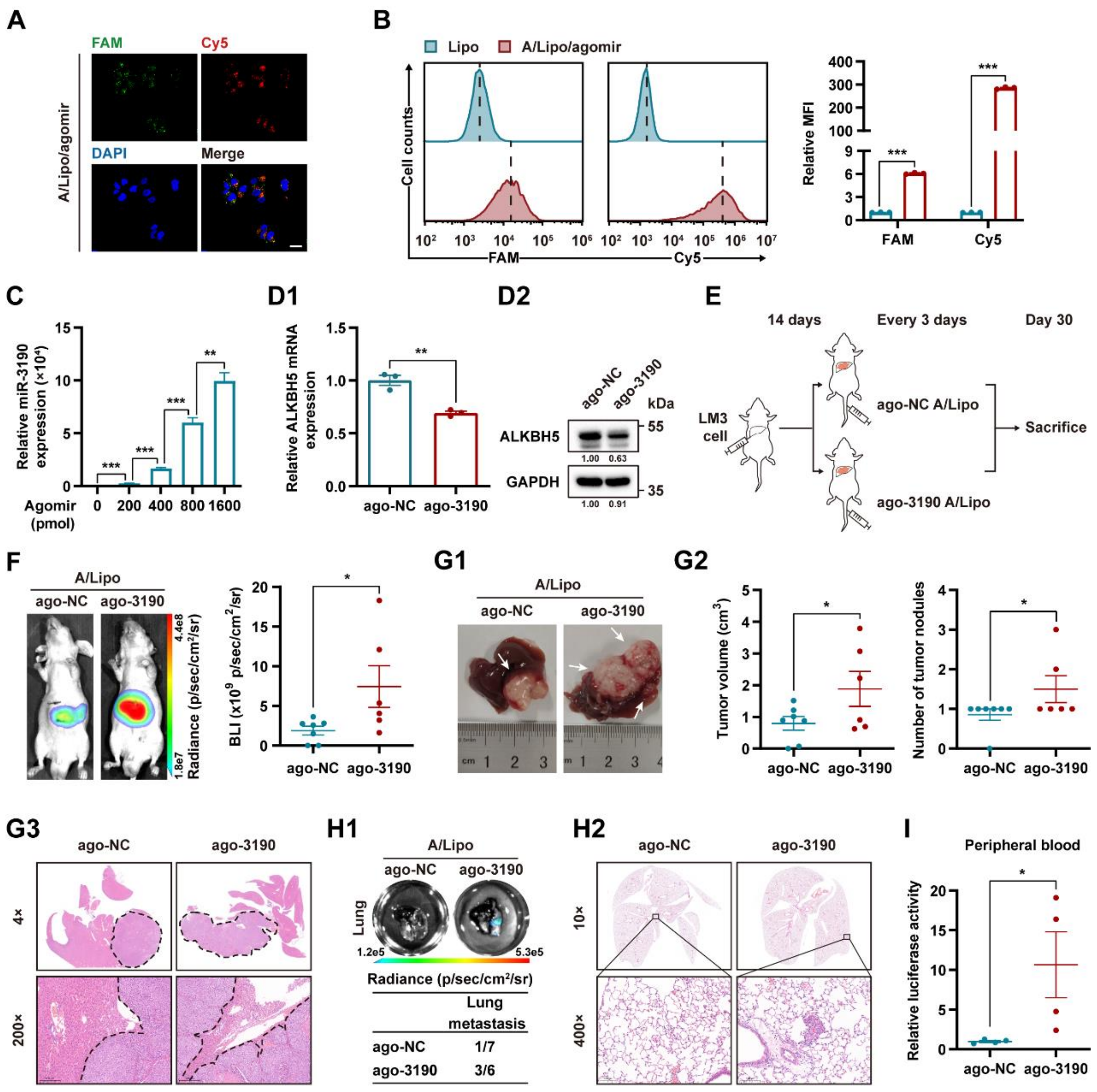

Figure S13. A/Lipo/agomir-3190 promotes metastasis of orthotopic liver tumor. (A and B) Confocal images (A) and flow cytometry analysis (n=3) (B) of fluorescent signaling intensity of LM3 cells treated with FAM-aptamer/liposome/Cy5-agomir-3190 complex. Blue, DAPI; green, FAM; red, Cy5. Scale bar, 20 μm in (A) Data are shown as fold change compared to their negative control cells treated with liposome alone in (B). (C) qRT-PCR analysis of miR-3190 levels in LM3 cells transfected with A/Lipo (A/L, 2 pmol: 1 μg) loaded with the indicated amount of agomir of miR-3190 (ago-3190) for 24h. (D) qRT-PCR (D1) and western blot (D2) analyses of *ALKBH5* expression in LM3 cells after treated with agomir (1600 pmol) or negative control (ago-

NC) encapsulated in A/Lipo for 48 h. GAPDH as loading control in (D2). (E-I) Mice were inoculated with LM3 cells for 14 days, A/Lipo loaded with ago-3190 (n=6) or ago-NC (n=7) were injected via tail vein every three days. (E) Schematic of orthotopic HCC xenograft model treated with A/Lipo/agomir complex. (F) Representative images and quantification of BLI intensity in mice at end point in each group. (G) Postmortem examination of orthotopic liver tumor. (G1) Representative macroscopic images of liver tumor. White arrows, orthotopic tumors. (G2) Quantification of tumor volume and nodules. (G3) Representative H&E staining of liver tumor. (H) Lung metastases evaluation. (H1) Representative ex vivo BLI (top) and quantification (bottom) of lung metastatic incidence. (H2) Representative images of H&E staining of lung metastatic foci. (I) Relative luciferase activities in peripheral blood are shown as fold change relative to the control group (n=4). Data are shown as fold change relative to HCC cells transfected with ago-NC in (C and D1). Data are shown as mean ± SEM. *$P < 0.05$, **$P < 0.01$, ***$P < 0.001$, Student's *t* test.

Supplementary Tables

Supplementary Table 1. Clinicopathologic characteristics of patients with hepatocellular carcinoma.

Clinicopathological variables	Number (n=110)	Percentage
**Gender**		
Male	90	81.82%
Female	20	18.18%
**Age**		
≤50	54	49.09%
>50	56	50.91%
**AFP (ug/L)**		
≤20	29	26.36%
>20	81	73.64%
**ALT (U/L)**		
≤41	81	73.64%
>41	29	26.36%
**AST (U/L)**		
≤40	77	70.00%
>40	33	30.00%
**GGT (U/L)**		
≤71	65	59.09%
>71	45	40.91%
**ALP (U/L)**		
≤130	94	85.45%
>130	16	14.55%
**HBV**		
Negative	0	0.00%
Positive	110	100.00%
**HCV**		
Negative	110	100.00%
Positive	0	0.00%
**Cirrhosis**		
No	30	27.27%
Yes	80	72.73%
**Ascites**		
No	94	85.45%
Yes	16	14.55%
**Tumor size (cm)**		
≤5	44	40.00%
>5	66	60.00%
**Tumor number**		
Single	85	77.27%

Multiple	25	22.73%
**Tumor capsule**		
No	50	45.45%
Yes	60	54.55%
**Macrovascular invasion**		
No	96	87.27%
Yes	14	12.73%
**Microvascular invasion**		
No	98	89.09%
Yes	12	10.91%
**Satellite nodules**		
No	100	90.91%
Yes	10	9.09%
**Extrahepatic metastasis**		
No	109	99.09%
Yes	1	0.91%
**Child-Pugh**		
A	106	96.37%
B	4	3.63%
**BCLC stage**		
0+A	71	64.55%
B+C	39	35.45%
**TNM stage**		
I	69	62.73%
II+III+VI	41	37.27%

Supplementary Table 2. List of upregulated miRNAs in BM4 cells compared to LM3 cells.

Probe name	Systematic name	Log_2 FC
A_25_P00016475	hsa-miR-4793-3p	6.330806
A_25_P00018713	hsa-miR-6832-5p	5.8186045
A_25_P00018089	hsa-miR-3912-5p	5.6530447
A_25_P00019073	hsa-miR-6757-3p	5.4976487
A_25_P00017910	hsa-miR-98-3p	5.4836483
A_25_P00018620	hsa-miR-6795-3p	5.359647
A_25_P00018622	hsa-miR-6880-3p	5.338094
A_25_P00017710	hsa-miR-3190-5p	5.329949
A_25_P00012130	hsa-miR-223-3p	5.316339
A_25_P00016617	hsa-miR-4440	5.241208
A_25_P00016683	hsa-miR-4707-5p	4.874387
A_25_P00015836	hsa-miR-4323	4.642298
A_25_P00017633	hsa-miR-5571-5p	4.162868
A_25_P00016367	hsa-miR-4701-3p	4.157846
A_25_P00017552	hsa-miR-5010-3p	4.0970044
A_25_P00018378	hsa-miR-6798-3p	4.0713844
A_25_P00015865	hsa-miR-4312	4.0108614
A_25_P00018585	hsa-miR-6756-3p	3.9977827
A_25_P00016414	hsa-miR-4754	3.9763532
A_25_P00018578	hsa-miR-6732-3p	3.931588
A_25_P00016519	hsa-miR-4731-3p	3.9218833
A_25_P00018768	hsa-miR-6779-5p	3.8613832
A_25_P00018924	hsa-miR-6793-5p	3.7165718
A_25_P00017560	hsa-miR-5684	3.6787367
A_25_P00014894	hsa-miR-551b-3p	3.2135265
A_25_P00012708	hsa-miR-92b-3p	3.1984105
A_25_P00012798	hsa-miR-548am-5p	3.187068
A_25_P00015068	hsa-miR-1227-3p	3.1792526
A_25_P00014678	hsa-miR-629-3p	3.158045
A_25_P00015463	hsa-miR-2116-3p	2.9691725
A_25_P00010874	hsa-miR-602	2.816343
A_25_P00012765	hsa-miR-550a-5p	2.5404112
A_25_P00018157	hsa-miR-6858-3p	2.5323336
A_25_P00018360	hsa-miR-1908-3p	2.5222101
A_25_P00016636	hsa-miR-1273f	1.9380343
A_25_P00015769	hsa-miR-4310	1.8701982
A_25_P00018592	hsa-miR-6765-3p	1.8174437
A_25_P00017909	hsa-miR-6507-3p	1.562686
A_25_P00018582	hsa-miR-6752-3p	1.4744577

A_25_P00018597	hsa-miR-6785-3p	1.4290612
A_25_P00019149	hsa-miR-7111-3p	1.3739908
A_25_P00018860	hsa-miR-7974	1.3631393
A_25_P00016295	hsa-miR-371b-5p	1.3135195
A_25_P00018609	hsa-miR-6861-3p	1.1532861
A_25_P00013651	hsa-miR-33b-3p	1.1477333
A_25_P00018540	hsa-miR-6751-3p	1.123775
A_25_P00013061	hsa-miR-933	1.106064
A_25_P00017179	hsa-miR-4652-3p	1.1010895
A_25_P00018599	hsa-miR-6760-3p	1.0707693
A_25_P00013271	hsa-miR-16-2-3p	1.02316
A_25_P00017529	hsa-miR-1273g-3p	1.0016232

Supplementary Table 3. Correlation between relative miR-3190 expression and clinicopathologic characteristics in HCC patients (n= 110).

Clinicopathological variables	Relative miR-3190 expression		P value
	Low 60	High 50	
**Gender**			
Male	50	40	0.652
Female	10	10	
**Age**			
≤50	30	24	0.835
>50	30	26	
**AFP (ug/L)**			
≤20	16	13	0.937
>20	44	37	
**ALT (U/L)**			
≤41	46	35	0.429
>41	14	15	
**AST (U/L)**			
≤40	46	31	0.095
>40	14	19	
**GGT (U/L)**			
≤71	37	28	0.547
>71	23	22	
**ALP (U/L)**			
≤130	52	42	0.693
>130	8	8	
**HBV**			
Negative	0	0	-
Positive	60	50	
**HCV**			
Negative	60	50	-
Positive	0	0	
**Cirrhosis**			
No	20	10	0.118
Yes	40	40	
**Ascites**			
No	51	43	0.882
Yes	9	7	
**Tumor size (cm)**			
≤5	28	16	0.118
>5	32	34	
**Tumor number**			
Single	50	35	0.097
Multiple	10	15	
**Tumor capsule**			
No	27	23	0.916
Yes	33	27	

**Macrovascular invasion**			
No	52	44	0.835
Yes	8	6	
**Microvascular invasion**			
No	57	41	**0.029**
Yes	3	9	
**Satellite nodules (correction)**			
No	59	41	**0.008**
Yes	1	9	
**Extrahepatic metastasis (fisher)**			
No	60	49	0.455
Yes	0	1	
**Child-Pugh (correction)**			
A	58	48	1.000
B	2	2	
**BCLC stage**			
0+A	43	28	0.087
B+C	17	22	
**TNM stage**			
Ⅰ	42	27	0.084
Ⅱ+Ⅲ+Ⅵ	18	23	

Correction, chi-square with Yates' correction; fisher, fisher's exact test.

Supplementary Table 4. Five potential binding sites of miR-3190 within the 3' untranslated region (3'UTR) of ALKBH5.

Gene name	Oligo sequence
hsa-miR-3190-5p	3'-ACCCCUGCAUCGACCGGUCU-5'
ALKBH5 3' UTR-WT1	5'-GAAGAAUAGAAUUGGCCAGG-3'
ALKBH5 3' UTR-MT1	5'-GAAGAAUAGAAUACCGGUCG-3'
ALKBH5 3' UTR-WT2	5'-CUGAUGCUGGAGUGGCCAGU-3'
ALKBH5 3' UTR-MT2	5'-CUGAUGCUGGAGACCGGUCU-3'
ALKBH5 3' UTR-WT3	5'-UCCCUUCUCCACUGGCCAGC-3'
ALKBH5 3' UTR-MT3	5'-UCCCUUCUCCACACCGGUCC-3'
ALKBH5 3' UTR-WT4	5'-GCAUGCAUCCAAGGCCAGAG-3'
ALKBH5 3' UTR-MT4	5'-GCAUGCAUCCAACCGGTCTG-3'
ALKBH5 3' UTR-WT5	5'-GCCCCUCUCAGGGGCCAGAA-3'
ALKBH5 3' UTR-MT5	5'-GCCCCUCUCAGGCCGGTCTA-3'

Abbreviations: WT, wild-type; MT, mutant-type.

Supplementary Table 5. List of different expressed and EMT-related genes in LM3 cells with *ALKBH5* knockdown.

Gene ID	Log_2 FC	*P* value
SLC9A4	4.343519811	5.41E-12
FRMD4A	4.030793699	3.40E-11
RP11-734K23.9	4.026932519	2.04E-25
LSAMP	3.970562615	1.98E-08
RP11-473O4.5	3.884844223	8.33E-10
LINC00886	3.84316788	3.80E-19
ASB4	3.795241634	1.20E-22
LINC00342	3.791552095	1.36E-35
LA16c-390E6.4	3.636500896	1.42E-08
HMCN1	3.636215556	1.63E-21
ZDHHC1	3.577889524	7.58E-08
GOLGA2P11	3.527927616	5.78E-12
AC159540.1	3.499065004	1.07E-30
AC108463.2	3.492849175	3.46E-09
LINC01355	3.485764166	4.68E-09
AC026348.1	3.421529054	1.99E-29
SOST	3.387401487	8.58E-09
ADGRL3	3.380263221	4.79E-15
CSRNP3	3.380135444	1.88E-08
RP11-21L23.4	3.340942407	7.91E-09
DNAH6	3.337480181	2.89E-08
SYTL5	3.3069181	2.26E-28
HSD17B3	3.269840819	1.48E-12
CACNB2	3.204263417	3.20E-08
RP11-705O24.1	3.189296432	6.49E-18
GOLGA8O	3.17845544	2.77E-07
AC139100.4	3.156655518	8.63E-08
RP1-151F17.2	3.136575352	6.63E-07
MYH3	3.125398016	2.77E-21
CFAP44	3.121864101	3.22E-30
RP11-797H7.1	3.119856653	5.48E-10
CFAP44-AS1	3.112169978	5.14E-10
RP11-499O7.7	3.102436099	1.49E-08
RP11-1012E15.2	3.100531709	3.03E-08
RP11-299H22.3	3.081939496	2.06E-18
IGFL4	3.075257985	1.14E-26
HELLPAR	3.067764302	7.09E-13

AGGF1P2	3.063948526	1.10E-08
DLGAP1	2.987433832	5.25E-07
CTD-2318O12.1	2.935573033	6.09E-08
CYP1B1-AS1	2.921758087	1.71E-15
RP11-284F21.9	2.910018344	9.99E-20
RP11-499P20.2	2.904406307	1.79E-11
PSMD6-AS2	2.891370536	3.75E-09
KCNQ5	2.860060788	9.99E-11
NME9	2.850768606	1.27E-06
SH3GL1P1	2.848805877	3.54E-07
RP11-280G9.1	2.846266253	1.69E-19
GSDMB	2.825168836	3.73E-18
RP5-984P4.6	2.804416203	6.71E-14
CTC-459F4.1	2.803473976	1.92E-09
TNS4	2.797727767	1.73E-19
RP11-295P9.3	2.769128681	1.66E-14
CMYA5	2.75320267	4.26E-08
PFN1P6	2.726519072	1.21E-07
RP11-159D12.2	2.720884825	4.19E-12
GOLGA8A	2.707008416	3.82E-33
GOLGA8B	2.681256507	1.70E-29
FAM184A	2.681148631	2.90E-12
SLC8A1	2.679911535	4.10E-06
SPINK5	2.67595759	3.24E-11
LINC00641	2.660957211	5.56E-07
MSR1	2.660884877	4.04E-11
RP11-392E22.10	2.647408895	6.39E-09
AGGF1P1	2.642567546	2.47E-06
RP11-426C22.6	2.641810287	8.65E-10
NAIP	2.639708926	1.36E-07
CH17-264L24.1	2.638261229	6.98E-07
LINC01021	2.627418741	2.74E-06
RP11-381E24.1	2.623304773	4.66E-06
TCTE3	2.611550617	7.40E-07
GABRE	2.610361895	5.82E-23
CCKAR	2.608487964	5.38E-22
AHSA2	2.608031743	2.49E-32
ADCY10P1	2.606261841	1.48E-06
RP11-384F7.2	2.593344122	4.25E-07
SOS1	2.590487475	6.55E-37
CTD-3065B20.2	2.585114671	1.00E-07
DYNC2H1	2.576521383	4.32E-23
AC093375.1	2.566083663	3.64E-09
FBXO43	2.553196891	1.38E-15

FBXL2	2.546850496	7.25E-11
TLX1	2.536432853	6.11E-07
CCDC191	2.530204994	3.87E-19
RP1-140K8.5	2.527845467	6.12E-33
RP11-328C8.4	2.527044865	1.99E-06
PFN1P2	2.524003403	2.14E-06
SDCBP2-AS1	2.520213579	3.01E-11
SLC25A27	2.508696449	1.06E-22
PWAR6	2.499685802	1.42E-06
HERC2P9	2.493145104	1.56E-24
CCNB3	2.478388476	1.03E-09
GABPB1-AS1	2.474791254	2.00E-26
RP11-1023L17.1	2.466717873	7.25E-10
CATSPER2	2.458372811	3.41E-11
SEMA3A	2.458225566	1.84E-31
RP11-392E22.11	2.456208089	9.86E-14
SLC13A4	2.441547557	4.20E-08
MSH5	2.432476871	2.24E-18
PER3	2.42957124	2.18E-18
LL22NC03-2H8.5	2.415568658	3.03E-13
CTB-13F3.1	2.410787789	1.58E-08
RP13-143G15.4	2.407385658	5.26E-08
RP11-114M5.1	2.398474873	2.71E-06
ZSCAN31	2.395288487	2.53E-25
BCO2	2.393174929	2.19E-07
HERC2P2	2.377071214	3.37E-27
PLCB4	2.377051015	1.82E-24
ITGA9-AS1	2.375840421	7.15E-12
AC073130.3	2.375548828	1.37E-07
RP5-1074L1.4	2.371378104	2.32E-06
CNGA1	2.365190447	3.99E-06
XXbac-BPGBPG55C20.2	2.364687479	5.01E-06
AC006042.7	2.363609635	2.99E-07
KB-1572G7.3	2.352327552	1.92E-06
PCF11	2.344662898	9.50E-30
SH3YL1	2.341543833	2.37E-07
MSH5-SAPCD1	2.340149703	3.30E-19
MRC2	2.337892423	1.30E-12
FNBP1P1	2.337580839	2.68E-09
GLDC	2.310768002	1.68E-07
ZNF804A	2.298143734	1.48E-07
SAPCD1	2.297436709	1.34E-07
HMGB2P1	2.296301527	1.72E-12
AP000648.5	2.293654611	1.17E-12

METAP1D	2.293546654	2.93E-13
RP11-894P9.1	2.290952617	1.81E-09
EPB41L4A	2.287171737	4.68E-07
SLC16A2	2.286864879	6.85E-07
RAD51B	2.282612289	8.44E-08
RP11-488L18.4	2.281079272	3.69E-20
CYP1A1	2.279569356	6.14E-25
KIAA1024	2.278327086	1.49E-06
LTB4R	2.266518885	3.20E-10
RP11-932O9.7	2.26561841	4.61E-06
RP11-419C5.2	2.252065772	4.44E-10
GOLGA8R	2.244987505	1.52E-06
RP11-412H8.2	2.237540949	4.03E-08
RP11-458D21.1	2.2355876	2.48E-06
SNX18P3	2.23159468	1.62E-20
ZSCAN30	2.22714491	1.87E-21
RALGPS1	2.216178385	2.37E-17
RP3-465N24.5	2.215662067	2.97E-06
YJEFN3	2.213105691	2.43E-15
CYP1B1	2.212065215	1.60E-23
IQCH	2.20881626	1.59E-07
TTC3-AS1	2.207217184	5.32E-14
RP11-707P17.2	2.203807815	2.34E-05
RP11-506K6.4	2.200845588	2.49E-06
CH507-42P11.6	2.200540738	2.80E-08
VCAN	2.198306208	4.54E-39
RNU4-25P	2.195168422	6.63E-06
RP11-228B15.4	2.18568896	2.01E-06
SLC9A2	2.185396875	2.12E-32
DICER1-AS1	2.185170092	1.68E-07
HERC2P3	2.177501829	1.71E-18
RP11-392E22.9	2.174759045	4.58E-19
PDE2A	2.163591199	6.01E-07
LRAT	2.159151203	2.66E-08
LL0XNC01-7P3.1	2.152816014	8.09E-06
LINC00894	2.149333518	1.57E-09
RP3-368A4.6	2.142708469	2.60E-09
SLC4A8	2.136191305	6.55E-09
AVPR1A	2.131648158	2.70E-06
TNFRSF25	2.119086775	3.82E-05
TNFRSF19	2.119023015	1.10E-12
PRUNE2	2.116069226	6.99E-06
LMLN	2.114853945	8.00E-19
FANCD2OS	2.114269931	1.53E-07

RBP5	2.112689186	1.99E-08
TCF7L1	2.110863849	1.60E-07
NKTR	2.109827537	5.46E-34
ATP6AP1L	2.10852246	4.22E-07
NPIPB15	2.107745331	8.63E-08
AC074286.1	2.102744536	9.23E-07
FAM95C	2.096632937	2.29E-20
TP73-AS1	2.093317826	5.45E-08
PLA2G2A	2.093153838	7.29E-06
RP1-27K12.2	2.087792639	5.82E-27
GSAP	2.087557144	1.06E-14
KIF14	2.086270622	1.78E-29
RP11-495P10.1	2.082990635	1.55E-08
AC083843.1	2.08101725	1.43E-09
PDE7B	2.075142105	1.93E-11
ELFN2	2.073852254	1.11E-08
F7	2.072858589	6.97E-09
RP11-803D5.1	2.063407852	8.32E-08
CICP14	2.063105523	1.96E-07
BTAF1	2.061212005	8.35E-31
ARMCX4	2.057028282	4.57E-16
GLCCI1	2.056604855	1.70E-25
HOXA4	2.049693555	1.69E-07
ULK2	2.041160377	7.66E-10
FER1L4	2.03628334	6.52E-20
CPT1B	2.03569213	4.86E-10
BRCA1	2.029596229	8.18E-32
GPR135	2.026607022	7.40E-09
ANKRD36	2.025241371	1.90E-23
PCDH7	2.024533635	2.95E-18
RNF139-AS1	2.023914295	2.81E-06
PRICKLE1	2.020274955	3.51E-08
HNRNPA1P16	2.016517158	7.18E-16
NEAT1_2	2.015395233	1.80E-11
RP11-392E22.5	2.01462967	2.28E-06
RP5-965G21.4	2.011071741	5.08E-06
AC074141.1	2.010607536	2.35E-05
SLC7A11-AS1	2.000134696	3.75E-24
GPR137C	1.998929354	1.05E-05
AC005154.6	1.998435207	1.36E-16
ZC3H6	1.99830662	8.19E-16
OGT	1.998260223	4.18E-36
RP11-218E20.5	1.997229362	3.87E-11
CRISPLD2	1.99291191	2.15E-11

MAP7D2	1.988057346	6.76E-15
AP000347.4	1.980301406	4.63E-06
PVRIG	1.980016425	1.19E-21
SPACA6	1.976294435	7.42E-10
RP11-186F10.2	1.968091456	2.91E-05
ZNF761	1.964796308	2.62E-07
HIST1H4H	1.959151997	1.42E-09
LINC01572	1.958884787	1.09E-05
MURC	1.954528812	1.10E-06
RP11-55J15.2	1.953764911	3.86E-06
KIF4B	1.953089423	2.37E-08
GP1BA	1.950109384	4.40E-08
DZIP3	1.942988046	3.01E-20
GCKR	1.940969272	2.99E-08
TTC3	1.939524692	1.90E-32
PABPC1L	1.928776388	2.64E-26
VPS13A	1.927416162	2.41E-29
CCDC14	1.923632366	2.74E-27
POLQ	1.923321043	3.92E-26
XRCC2	1.91219623	7.19E-23
GNAZ	1.909979125	1.32E-09
APOB	1.908486186	8.89E-16
RP11-448A19.1	1.907514168	6.02E-09
ZNF497	1.902787554	7.39E-06
PDXDC2P	1.901687446	1.02E-16
RFX3	1.898988007	1.06E-06
RP11-504P24.3	1.89268179	2.78E-06
ABC12-49244600F4.4	1.890031092	1.24E-04
RP11-242D8.1	1.888587748	4.19E-18
MUSK	1.885919093	2.13E-06
SLC1A2	1.885755203	1.12E-04
ANKRD1	1.881604581	4.54E-07
RP11-196G18.22	1.880925986	6.96E-23
MTCO1P53	1.877716597	3.34E-06
SCARA5	1.877361945	1.73E-05
LINC01239	1.87674435	3.64E-07
RP11-342K6.1	1.876333632	1.77E-05
ABCA4	1.876152952	6.79E-32
CACNG8	1.872082708	4.86E-05
PRSS35	1.868392773	1.17E-09
ZDHHC11B	1.86250825	3.11E-14
LINC00106	1.861440965	8.07E-05
VCAN-AS1	1.860339396	2.14E-10
FAM184B	1.858577682	4.03E-05

MUC3A	1.854084623	5.34E-06
AC002480.5	1.852756661	8.70E-13
MALAT1	1.850621262	5.90E-04
PWAR5	1.84986863	7.46E-09
RP4-751H13.7	1.849244655	1.02E-06
ZNF655	1.847506818	4.13E-25
BHMT	1.846334766	5.34E-05
ITGA9	1.845068955	6.48E-05
CTD-2525I3.5	1.84476834	6.37E-05
RP11-575L7.4	1.844166154	7.17E-05
SYTL2	1.840233386	3.68E-31
RP11-196G18.24	1.839062787	1.70E-09
RNF32	1.837352838	7.43E-08
KCNQ1OT1	1.836086543	9.38E-23
LGI4	1.83594161	8.19E-11
RP3-508I15.21	1.834409435	3.14E-05
LHFP	1.834403328	2.26E-18
RP11-284F21.10	1.825075747	1.32E-27
MYO1D	1.824950258	3.42E-06
TBC1D3	1.824482835	1.37E-07
RP11-707P17.1	1.824288712	1.03E-04
RP11-335G20.7	1.823525198	2.97E-05
GATA6-AS1	1.820890994	3.37E-05
SGMS1-AS1	1.818328024	4.53E-05
AP000769.1	1.816795246	2.12E-15
EIF2S2P2	1.816459886	1.33E-05
AL354822.1	1.816347628	3.21E-04
FANCI	1.815630958	3.36E-30
RP11-315O6.1	1.810841378	2.65E-06
LA16c-349E10.1	1.810449061	2.99E-05
CASC2	1.810314249	1.85E-06
MIR4453	1.809882624	6.16E-07
CDK18	1.809307011	4.01E-06
EHF	1.807407171	2.01E-24
MIR600HG	1.803747823	4.31E-10
WDR27	1.80203884	9.61E-18
RP4-613B23.1	1.800074724	2.96E-12
RP11-703M24.5	1.799997184	1.43E-07
EML1	1.799738007	9.07E-09
AC010883.5	1.79810519	5.33E-06
CTD-2653D5.1	1.79790116	9.48E-07
CCDC146	1.797844218	2.29E-05
ADGRG2	1.797321578	1.68E-05
RP11-152F13.8	1.796885656	1.25E-10

ZC3H12B	1.793645754	3.30E-08
GOLGA2P5	1.793505831	4.26E-20
RP11-2B6.2	1.790401909	4.00E-05
RP11-927P21.5	1.78860378	4.55E-10
KNTC1	1.788171182	2.54E-28
RP5-1087E8.3	1.78728941	5.66E-15
ZNF577	1.784984264	5.59E-06
RP11-257O5.2	1.784479824	5.32E-06
SLC45A1	1.783478341	3.23E-05
INTS6	1.782816464	3.36E-21
PLCE1	1.779614187	8.46E-12
RP11-1376P16.2	1.779004935	1.06E-06
XXbac-BPG283O16.9	1.777355386	1.26E-07
LGR5	1.775897216	9.57E-13
CCDC190	1.772168802	1.66E-06
RPL38P4	1.772161669	5.22E-07
RP1-283K11.2	1.76886527	2.04E-05
RP11-443P15.2	1.766753312	1.71E-23
KATNAL2	1.763511768	2.01E-10
RP11-197K6.1	1.760882287	1.10E-06
DNAH10OS	1.76046381	2.06E-04
ACCS	1.759517898	2.69E-11
IFT80	1.759036096	2.05E-19
TENM1	1.758152441	1.80E-12
LONRF2	1.757669202	2.77E-26
RP11-265D17.2	1.752802317	2.76E-04
IGLON5	1.745797993	7.69E-10
MRVI1	1.743925608	5.63E-10
IGSF1	1.743871987	9.90E-15
NEAT1_3	1.743393984	2.10E-20
RGPD1	1.742851638	8.76E-05
DNAH5	1.742299756	1.33E-10
TMCC1-AS1	1.741395143	3.51E-11
MIRLET7DHG	1.738086592	6.27E-05
CXCR6	1.737948878	1.97E-04
BANK1	1.732902826	3.08E-12
CCDC150	1.732757931	5.98E-12
SMAD9	1.729855352	4.41E-06
RHPN1	1.727723911	1.29E-18
LINC00470	1.725815701	4.91E-05
WASH5P	1.725626713	1.55E-13
RP3-368A4.5	1.719828667	1.16E-07
OFD1	1.718336775	1.82E-19
RP11-261C10.5	1.717558719	4.04E-11

GPR15	1.714389617	1.65E-19
RP11-517B11.7	1.712736929	7.71E-06
STAB2	1.712199154	7.02E-05
ZNF37BP	1.712076257	3.31E-18
ZGRF1	1.71081521	3.68E-14
LUCAT1	1.710728259	4.95E-20
CACNA2D1	1.709713237	2.21E-04
ZNF532	1.708420793	3.60E-28
CEP192	1.706561308	1.06E-25
LINC00648	1.705947348	1.43E-04
TRIM66	1.704781653	3.83E-11
AF230666.2	1.704276892	6.05E-20
CCDC168	1.70258252	1.41E-04
NEIL1	1.701747892	7.53E-08
ALX3	1.700835295	4.83E-05
CARF	1.698389799	6.34E-05
FAM118A	1.697121832	3.17E-24
CCDC18-AS1	1.696801915	6.29E-08
JAZF1	1.692690445	4.01E-10
RP11-334C17.5	1.692498847	1.20E-11
AP000695.6	1.692284221	7.63E-07
SP2-AS1	1.691857184	1.64E-05
RP1-170O19.22	1.68941644	8.00E-06
CD86	1.688670258	3.55E-04
RP11-296E7.1	1.688133123	2.32E-18
LINC01515	1.686066031	3.70E-05
LRRC37A17P	1.685905141	4.71E-10
CAPRIN2	1.684436244	6.34E-25
GREB1L	1.683530294	1.05E-19
C1RL-AS1	1.682113007	1.07E-15
RP11-485O10.3	1.68183647	1.86E-06
TSSK4	1.681274117	3.09E-04
ADAMTS6	1.680455498	4.21E-05
RP11-1035H13.2	1.679170147	3.41E-12
MYO5C	1.679151788	1.57E-26
USP32P3	1.678889038	5.44E-06
GOLGA6L4	1.677619876	1.73E-10
PLEKHH2	1.677612421	6.79E-25
AGTPBP1	1.674041121	1.60E-21
CCDC171	1.672738611	1.80E-05
RP4-694B14.8	1.669866214	2.27E-04
MAPT-AS1	1.668643066	6.99E-05
AK7	1.666088069	2.32E-10
GRIN2D	1.662911874	3.22E-05

ZNF75D	1.662886146	3.91E-18
SCAPER	1.662249133	1.60E-19
CEP126	1.662028491	3.69E-20
RP11-410L14.1	1.661009416	1.55E-04
CCDC57	1.661006464	5.35E-26
INTS2	1.659967217	1.38E-19
MAGEC1	1.657595836	1.19E-10
CPHL1P	1.652845802	4.84E-04
STAG3	1.652149804	5.19E-27
EML6	1.651859189	3.43E-10
PDE11A	1.651704404	1.58E-06
MASP1	1.650247757	4.85E-05
ZNF546	1.6496765	1.18E-06
FANCD2	1.643283033	5.06E-24
CCDC187	1.640689101	2.99E-04
NPHP1	1.640170313	3.20E-11
RYR2	1.638958161	9.12E-14
RP11-159N11.4	1.638851995	5.42E-04
SYNE2	1.637138089	5.15E-29
COL5A2	1.6356129	8.26E-24
CCDC180	1.63426539	1.81E-14
PRRX2	1.634175663	5.51E-08
LHX4	1.633957222	2.38E-05
CTD-2366F13.1	1.631922753	6.33E-04
RAB11FIP1P1	1.63000105	3.27E-05
ZBTB38	1.629510821	3.50E-28
ADD3	1.629455612	6.90E-30
AC025918.2	1.62748524	6.98E-07
C22orf46	1.627097091	1.10E-24
UPB1	1.626884925	1.84E-07
TEKT2	1.625820481	2.04E-05
PAQR8	1.624907817	2.88E-17
KRT83	1.622499415	2.20E-05
ATM	1.621045273	4.85E-27
RP3-467L1.4	1.618967745	9.58E-05
AC009501.4	1.617579585	1.19E-22
SRRM2-AS1	1.616580492	2.33E-05
USP9Y	1.614198429	1.59E-16
RP11-471M2.3	1.613536802	2.57E-04
RP11-379F4.4	1.608641862	3.78E-07
PLCXD2	1.608469591	8.44E-18
TRDMT1	1.608362903	6.86E-07
RP11-444D3.1	1.60787436	4.14E-05
KCNQ3	1.607820948	3.75E-19

AC005592.2	1.604876243	9.95E-05
PHKA2-AS1	1.60331598	1.08E-06
ADGRA3	1.603274923	6.60E-25
CYP3A5	1.596805728	9.93E-23
RP11-213H15.4	1.595486529	1.79E-10
EFEMP1	1.595372733	2.16E-04
GOLGA6L5P	1.594204032	2.27E-07
CCNL1	1.593885944	1.09E-24
RP11-379K17.12	1.590723138	1.29E-05
USP40	1.590020486	9.55E-18
SLC2A3P1	1.588830028	1.54E-16
GARNL3	1.588228933	3.42E-09
ADAMTS13	1.587436874	3.71E-06
COLCA1	1.586762599	4.92E-24
ASPM	1.586571363	1.27E-15
CNTRL	1.586276119	1.70E-24
RP13-270P17.2	1.586075712	9.40E-05
TAF1L	1.584987871	2.30E-05
ATAD5	1.584521111	1.50E-17
AP1G2	1.581756513	3.17E-19
TBC1D3E	1.579247845	9.28E-05
L3MBTL1	1.578658537	5.80E-05
RP11-1113L8.1	1.578125035	1.30E-05
TMEM154	1.575610612	5.07E-15
SULF2	1.575064075	1.82E-19
NUDT7	1.573931812	1.61E-04
CTA-204B4.2	1.572524403	5.02E-07
GNA14	1.571606267	3.02E-04
RP11-273B20.1	1.571606267	3.02E-04
AMY2B	1.571282123	1.33E-05
CFAP69	1.569645074	5.79E-05
AK9	1.569385941	9.36E-08
DENND4A	1.566981587	2.77E-14
CDC42BPA	1.566804374	1.85E-30
PTPRM	1.566550161	2.42E-28
RP11-575L7.2	1.565370367	3.71E-04
AGAP6	1.564663844	5.48E-14
AGAP11	1.564015829	1.02E-04
SPDYE2B	1.560964089	4.33E-07
RP11-681B3.4	1.560189733	4.21E-05
DMXL2	1.560164	1.53E-23
EPM2AIP1	1.560118813	3.28E-15
RP11-21L23.2	1.557397552	4.63E-05
RP11-261C10.4	1.556930211	1.38E-07

RP11-227H4.5	1.556826456	1.93E-05
MYLK	1.556810383	4.18E-21
RP11-175P13.3	1.556434798	3.53E-07
PAXBP1	1.55637838	6.98E-27
PNISR	1.556377091	2.11E-29
LAMA3	1.55625525	4.80E-32
CCDC33	1.552880796	1.59E-14
RP11-804M7.1	1.552003527	2.46E-04
ADGRG6	1.551480501	2.18E-18
RP5-855D21.3	1.550993531	2.76E-04
RP11-493P1.2	1.550937255	2.67E-04
CTD-2368P22.1	1.549740508	3.69E-06
LL0XNC01-237H1.2	1.548580274	8.79E-08
AP000347.2	1.547952189	4.19E-13
RP11-88I18.2	1.547926725	3.60E-04
MICAL2	1.546652047	8.77E-26
PRDM10	1.546318856	9.88E-20
ZNF767P	1.544606423	4.27E-08
RP5-1112D6.8	1.543497876	1.50E-04
RP11-332H14.2	1.542172574	4.69E-08
SPDYE5	1.541118077	4.36E-06
GDPD4	1.540162102	4.47E-07
PHKA2	1.538658833	3.37E-19
C5	1.538383742	3.31E-16
ZNF860	1.537633004	2.17E-08
AC078883.4	1.536290815	3.11E-16
TTC12	1.532927594	9.03E-18
RP5-1120P11.1	1.532831474	7.28E-05
SMCHD1	1.531907444	2.09E-27
PDE9A	1.529743942	6.55E-13
RP11-587P21.2	1.529388692	1.13E-07
RP4-773N10.4	1.528277112	4.32E-07
CENPF	1.526627476	1.19E-32
APLF	1.526439121	5.00E-09
SPAG4	1.525077863	4.80E-10
AC062029.1	1.524352813	7.19E-05
USP6	1.524170702	6.42E-04
USP34	1.523870816	2.25E-28
LRRC37A4P	1.520228688	9.73E-20
GOLGA6L3	1.520193123	8.15E-05
TMEM175	1.520081168	1.81E-08
POLA1	1.51977382	9.34E-23
GLDN	1.517120679	7.85E-16
PSD3	1.515010281	1.78E-29

KAT2B	1.513470617	2.15E-17
OFCC1	1.512430951	4.78E-04
RP11-464F9.1	1.511256128	5.21E-09
RP11-66N24.3	1.510895399	2.05E-14
CBSL	1.509982346	5.34E-19
REV3L	1.509199578	4.12E-17
AGO4	1.506884956	9.72E-16
RP11-37B2.1	1.506630161	2.23E-10
RP11-63M22.2	1.506297432	1.34E-12
DNHD1	1.506110055	1.58E-18
KIAA1107	1.50373144	7.12E-06
RP11-46C24.7	1.503317673	1.10E-03
RP11-6O2.4	1.503291161	1.47E-04
CTC-471F3.5	1.502927376	2.13E-08
RP11-403P17.6	1.502007715	6.75E-05
CROCCP3	1.501637437	4.50E-08
ASGR1	1.500548283	4.28E-04
WDR90	1.499352058	8.75E-12
MYO9A	1.498427004	6.54E-21
AC002480.3	1.497263009	1.57E-11
CEP170	1.496702483	2.48E-28
ABCA5	1.496635291	5.72E-10
USP49	1.495849053	2.64E-09
EFCAB13	1.49515015	1.01E-05
CBS	1.494462293	7.85E-19
DNAH12	1.493277498	4.54E-05
ST18	1.492356036	6.74E-09
VSX1	1.491754809	2.70E-04
MSANTD2	1.490906686	2.87E-16
RP11-18H7.1	1.489535546	5.49E-04
ZMYND8	1.48947836	5.00E-29
RALGAPA1P1	1.488152773	1.20E-04
TSC22D1-AS1	1.487547159	3.28E-05
SAMD5	1.48566766	6.48E-22
ZNF234	1.485361425	1.86E-15
TMEM67	1.485193353	1.48E-10
ALDH1L2	1.484212247	1.89E-14
RAD51-AS1	1.483548402	3.39E-07
CEP135	1.482887601	6.16E-13
RP11-874J12.4	1.481681026	4.95E-04
CDKL1	1.480999465	3.85E-06
TBC1D3C	1.480803154	7.68E-05
CDH1	1.480767093	1.66E-14
ARHGEF9	1.479483962	4.22E-24

MALL	1.479388575	3.58E-27
NABP1	1.479337663	8.49E-22
ZNF367	1.477173891	6.71E-24
TPR	1.477113041	5.78E-32
JAML	1.473334152	7.56E-05
BRCA2	1.472657322	1.57E-11
GS1-259H13.13	1.47157461	8.07E-10
MCM8	1.471223667	1.07E-25
ALMS1	1.470619986	3.04E-22
SH3RF2	1.468773932	4.68E-18
SLF2	1.466548755	9.71E-20
CEP170P1	1.465793937	1.41E-17
AC078883.3	1.46520544	2.71E-23
MEST	1.464901383	1.46E-11
ZNF862	1.464885416	1.17E-10
KIF27	1.462792255	9.70E-12
NTSR1	1.462475796	1.04E-03
LINC00654	1.461733251	2.98E-13
RGN	1.460658632	2.88E-13
CAST	1.460509785	4.94E-33
PPFIA4	1.460184232	1.60E-08
DPY19L3	1.459115384	1.36E-16
BMS1P1	1.456397579	8.32E-08
ADD3-AS1	1.456322106	9.15E-13
FAM179A	1.455502564	4.75E-04
ANKRD36C	1.45529012	1.65E-20
GNB3	1.45500109	9.49E-08
PCYOX1L	1.454813712	5.20E-10
BRIP1	1.454374427	6.69E-18
LINC00704	1.454241367	1.67E-04
GPR161	1.452999638	6.61E-14
RP11-415J8.3	1.452001954	3.13E-04
MAML2	1.451945609	9.83E-17
ERCC6L2	1.451555989	5.32E-15
ATP8A1	1.449566568	4.08E-06
UACA	1.446799112	2.60E-27
TSSK3	1.445532915	4.74E-04
GGACT	1.445438895	6.53E-07
PKD1L2	1.443189643	2.20E-12
CMTM1	1.442476687	8.35E-05
RP11-231C14.7	1.442155971	3.32E-03
TRAF3IP3	1.441738659	8.37E-04
CSGALNACT1	1.441577043	2.65E-08
TRIM52	1.440478715	3.32E-15

NEAT1	1.439228649	3.15E-27
RP11-140H17.1	1.436322227	1.76E-07
AC125232.1	1.43412463	3.67E-06
SOX5	1.433154678	4.41E-09
ATR	1.433147397	3.80E-23
ANGPTL2	1.433125409	2.17E-26
INTS6-AS1	1.433048939	1.48E-04
RP11-324I22.4	1.429859117	4.85E-04
RP11-218M22.1	1.425939961	7.05E-12
ZKSCAN8	1.42520734	4.70E-17
ARFGEF1	1.421630451	1.86E-27
ANLN	1.420833652	1.91E-30
KDELC2	1.420826224	4.58E-21
EHBP1	1.418775364	1.33E-29
MIR34A	1.418703029	2.65E-10
WHAMMP2	1.416936422	1.02E-04
UBE2Q2P1	1.416313997	1.82E-09
BTBD8	1.416003912	1.94E-06
RP11-54D18.4	1.415703447	6.06E-04
CEP295	1.415423575	2.81E-23
RTEL1	1.415171648	3.33E-19
ARHGAP11B	1.414461732	2.25E-18
PTBP2	1.414245011	2.37E-08
SBF2	1.413186227	4.65E-22
TCEA3	1.412688266	4.37E-14
CFAP126	1.412619689	1.09E-06
CCDC183-AS1	1.410116703	1.36E-12
RP4-800G7.2	1.408610212	3.88E-05
ADORA2A-AS1	1.408386134	6.22E-06
KIF15	1.407488011	3.47E-25
WDR35	1.406998894	1.26E-11
RP11-757O6.6	1.406405939	1.35E-03
RTEL1-TNFRSF6B	1.405737249	4.78E-19
RP11-87H9.4	1.404967073	7.78E-04
AC005336.4	1.402957827	4.04E-11
KIAA0232	1.401468006	1.42E-26
ARHGAP21	1.401098436	1.29E-29
L3MBTL4	1.401064412	5.13E-08
POLN	1.400733129	2.99E-05
PYROXD2	1.400186165	5.15E-07
CTD-2233K9.1	1.400043202	9.93E-04
NEK1	1.399224974	4.12E-17
BOD1L1	1.396414951	1.41E-28
GPLD1	1.394709521	3.40E-06

CCDC162P	1.394177477	1.52E-06
DEPDC1	1.39300856	2.14E-15
SLCO2B1	1.39297362	2.99E-09
RFX6	1.392177212	7.18E-05
TNIK	1.392070329	1.29E-28
ANKRD36B	1.390699754	6.83E-15
CHD9	1.390258714	3.73E-23
E2F8	1.39022225	4.27E-11
TTLL1	1.389557192	2.34E-06
RP11-89K21.1	1.38775096	1.01E-04
SLC7A2	1.387267676	1.42E-17
LANCL1	1.386965899	6.45E-12
CTC-506B8.1	1.385505434	2.03E-23
ITGA6	1.384947409	9.76E-30
GUSBP11	1.384483402	1.65E-13
PHKB	1.38291296	1.13E-23
BIVM	1.381609043	1.69E-07
AKAP6	1.381517602	1.24E-05
C5orf42	1.381162979	8.83E-13
RP11-2E11.6	1.3797505	6.49E-07
ZMYM4-AS1	1.378857359	6.53E-05
THSD4	1.377414906	1.23E-14
DNAJC13	1.377389018	1.55E-26
ENC1	1.377378333	5.06E-24
ZNF248	1.37669241	3.98E-11
RHOH	1.375751011	2.26E-04
SYNM	1.375654748	3.25E-05
IGFBP3	1.374474038	4.15E-12
ATP5G1P4	1.372610076	8.83E-04
LRRC46	1.372147039	9.33E-04
IL18R1	1.371656393	1.06E-06
BMS1P4	1.371271529	7.48E-05
RP11-567M16.6	1.370875892	1.17E-04
RP11-54O7.1	1.370371376	2.21E-03
RGL3	1.369255146	2.94E-04
AKAP12	1.368400793	4.74E-31
RP11-463J10.3	1.368217506	1.13E-03
SRBD1	1.368159917	1.35E-15
RP1-102E24.6	1.367423635	1.92E-04
CCNL2	1.366653417	5.41E-23
RP11-258C19.7	1.366531587	1.78E-04
RP11-219A15.1	1.366305445	2.21E-03
MAP1B	1.366046002	2.52E-04
NIPAL2	1.365971102	9.57E-12

RP11-855A2.2	1.365687945	7.48E-06
RP11-658F2.8	1.365548516	7.27E-04
COL6A3	1.365039025	1.90E-22
RP11-101E7.2	1.364449495	1.39E-03
RP11-677M14.7	1.364194231	6.49E-04
DLGAP1-AS2	1.363908222	7.17E-09
AGAP5	1.363622558	2.98E-06
RP11-488L18.8	1.363619555	4.08E-04
FRMD3	1.363602509	1.54E-24
HDAC6	1.36253633	4.04E-23
TTC3P1	1.359694928	3.51E-08
TBC1D3H	1.35957089	2.81E-05
MYBL1	1.359510737	2.08E-18
NEK11	1.359095568	1.67E-07
CEP128	1.357821714	9.66E-15
EDRF1-AS1	1.357434859	1.26E-05
FRG1EP	1.357072808	1.60E-04
CCDC40	1.356158593	1.26E-19
CHAD	1.355773903	1.08E-05
HEATR5B	1.355410113	1.18E-17
ARID4A	1.355192101	3.76E-18
COL4A5	1.351973691	5.16E-15
CHRM3	1.351641136	2.74E-05
MTCL1	1.350159688	2.37E-28
C1QTNF6	1.349342364	1.63E-07
CYP4F12	1.34861872	1.86E-11
CTD-2270P14.1	1.348334324	1.23E-12
HAAO	1.348084181	3.80E-04
RIBC1	1.346872169	3.75E-09
ZNF519	1.345798974	9.93E-11
KB-1572G7.2	1.345152198	4.24E-10
RP11-359B12.2	1.345003462	1.76E-05
SNX13	1.343520819	1.41E-19
UTRN	1.343467683	9.35E-27
ZNF789	1.342900943	5.00E-14
ITGB4	1.341697911	1.78E-24
FHAD1	1.340973517	5.50E-13
FAM229A	1.339872631	1.07E-04
PROC	1.339771756	8.29E-04
TXLNGY	1.339751478	2.52E-17
PRC1-AS1	1.338488732	9.57E-20
CTC-471J1.11	1.337940802	1.73E-03
C21orf58	1.33723666	1.54E-10
DLG1-AS1	1.336443937	1.77E-04

SPIDR	1.336170353	5.44E-21
FANK1	1.336109206	1.23E-04
GLI2	1.335252904	1.76E-12
CCDC88A	1.334784503	1.65E-22
PCAT7	1.333755774	2.11E-04
KLF12	1.331538261	8.84E-07
MORN1	1.330247585	2.38E-11
FAM208A	1.33003734	7.60E-26
AC002116.7	1.329414001	7.10E-05
KIAA1468	1.328863421	8.73E-17
PEX1	1.328538038	2.25E-18
CDC45	1.328285287	1.29E-24
ZNF507	1.328112749	3.90E-23
RP1-127H14.3	1.327287382	9.84E-04
AGAP10P	1.326808168	3.04E-04
MYSM1	1.326289329	1.19E-13
RP11-354M1.2	1.325751984	1.06E-04
ASMTL-AS1	1.324562632	1.57E-08
DNAH10	1.323305442	1.43E-03
GOLGA6L9	1.323295003	3.12E-10
KIF23	1.322581208	1.84E-25
USP46-AS1	1.322415091	3.62E-05
CLOCK	1.322278829	2.34E-20
SLC4A4	1.322052563	5.63E-14
AC008277.1	1.321803202	1.44E-04
CHN2	1.320077459	1.33E-21
RP11-540B6.6	1.318795485	4.69E-08
EEA1	1.318337735	9.38E-23
AGAP4	1.318335199	1.67E-06
PDK1	1.318022831	2.97E-12
PLA2G6	1.317545344	8.03E-07
C5orf63	1.314489929	1.42E-03
CENPE	1.314471767	4.15E-24
TUG1_3	1.31414273	8.80E-12
LYVE1	1.314135822	1.84E-03
ZNF449	1.313485785	8.21E-13
COPZ2	1.31222079	3.08E-06
LMO7	1.309886801	1.33E-26
CAMSAP2	1.30986772	7.02E-26
KCNT2	1.309769919	1.23E-04
RP11-996F15.2	1.309582169	3.39E-08
FMO5	1.307502188	7.33E-26
IQGAP3	1.306080457	3.35E-27
SLC7A11	1.304537323	1.51E-27

KIAA1109	1.304059344	4.32E-21
RP11-420O16.2	1.303277753	1.49E-03
MAPK10	1.303104521	1.50E-06
AC007906.1	1.302719326	1.71E-07
RP11-98J23.2	1.302261473	1.59E-03
SBF2-AS1	1.302011553	6.03E-11
ZNF436-AS1	1.301823584	1.61E-04
RPL32P3	1.30150029	1.29E-12
TBC1D3L	1.301018776	6.24E-07
AFG3L1P	1.3007779	8.20E-15
SPDYE2	1.300474739	7.62E-04
YTHDC2	1.299618678	4.14E-17
IBTK	1.299497688	4.00E-22
PRC1	1.299329034	1.54E-29
AC007773.2	1.299306304	3.70E-04
GATS	1.298909611	2.06E-22
ARHGEF28	1.297132198	4.86E-24
GALNT18	1.296508541	2.86E-09
BUB1B	1.29630459	7.39E-27
CTD-3060P21.1	1.295209618	8.40E-06
LENG8-AS1	1.295153499	3.13E-07
RP4-784A16.2	1.29475548	2.44E-08
RGPD6	1.294200639	1.64E-11
RP11-57H14.4	1.293866441	1.15E-03
KIRREL3	1.2934125	2.06E-03
LRP8	1.292851574	1.45E-27
FOS	1.292439377	5.89E-16
RP5-966M1.6	1.291430733	4.11E-04
INVS	1.29124486	4.59E-17
CFAP97	1.291116219	9.10E-22
PAN2	1.29066547	6.10E-17
HSFX2	1.29022759	2.55E-03
IFT172	1.28942658	8.12E-17
AXL	1.289044011	3.52E-20
RP3-467K16.2	1.288892853	2.86E-04
WDR19	1.288626455	9.45E-17
DDX60	1.287444848	6.21E-10
RP11-379H18.1	1.287256445	1.29E-07
ZNF621	1.287076442	1.70E-16
AC092718.1	1.286821394	2.09E-03
ZBED8	1.286287402	4.80E-04
VPS13C	1.285841002	1.08E-19
RP11-23J9.4	1.285103467	6.51E-12
FERP1	1.285037309	4.75E-06

SRP14-AS1	1.284828155	2.22E-03
RHOT1P1	1.284189705	1.16E-03
PLAG1	1.284105597	4.14E-11
IFT81	1.282567069	1.49E-15
CLMP	1.281878446	4.97E-16
PKD2	1.281484151	1.72E-17
SPDYE6	1.280983269	6.42E-04
HIBADH	1.280756937	5.88E-23
ACKR4	1.280325734	4.68E-04
CHN1	1.280242023	1.11E-11
RP5-1061H20.4	1.278107412	7.10E-06
RBBP6	1.277575606	9.80E-24
TAF1	1.276523601	1.01E-25
FLT3LG	1.276073554	1.10E-03
GAREM2	1.275972255	5.02E-06
MR1	1.275247357	4.53E-05
ZNF382	1.274111158	1.77E-03
ARMC3	1.272245173	2.41E-03
WBSCR27	1.272178308	2.06E-03
RUNX2	1.272015192	3.89E-06
MXRA8	1.27178863	4.93E-06
TAB3	1.271340897	2.61E-13
KIF11	1.270740021	2.21E-24
CFAP70	1.269850271	1.80E-03
MDN1	1.269484591	1.13E-26
EMILIN3	1.268513912	5.18E-05
ABCC2	1.268311742	5.41E-30
FAM86B1	1.268150064	3.59E-06
ZBTB37	1.267303313	4.34E-09
CREBZF	1.266506899	2.79E-25
CEP350	1.264584073	6.16E-23
RP3-323A16.1	1.263018719	1.01E-03
CRIPAK	1.263011792	3.63E-03
CDK3	1.262834817	4.67E-09
BCAN	1.262299801	1.81E-16
TRAF1	1.261232109	5.78E-07
RP11-430B1.2	1.259883201	2.75E-04
PPP1R3C	1.259664726	4.18E-07
TMEM161B-AS1	1.259200562	6.01E-07
PTGIS	1.256000114	1.01E-05
RAB11FIP2	1.254544241	8.85E-13
RP11-546D6.3	1.253996429	2.11E-03
C5orf56	1.252951132	2.41E-03
CEP112	1.252535452	7.21E-13

GPRASP1	1.251457936	6.98E-08
PRELID3A	1.25129531	8.32E-09
VWA8	1.250301361	4.49E-14
MEF2C	1.250147991	4.79E-09
SOCS4	1.25000293	2.02E-16
AGAP9	1.249851819	5.02E-06
RP11-773H22.4	1.248741225	2.09E-03
PDE8A	1.248323269	1.86E-17
RP11-285F7.2	1.248251419	2.87E-03
ZFC3H1	1.24612601	2.79E-22
LINC00624	1.244107664	1.88E-04
NBPF8	1.242840809	1.20E-10
KIAA0586	1.242537059	4.94E-14
RP11-284F21.7	1.242128652	2.63E-10
HIVEP3	1.242040845	5.79E-07
KIF5B	1.2417822	4.13E-27
UBR1	1.241729137	3.04E-15
LINC01484	1.241661136	1.06E-09
CDON	1.240831745	3.00E-10
THNSL1	1.240431118	2.93E-11
LRRC37A	1.240349476	5.82E-10
PDPK2P	1.240191753	6.89E-08
SLC24A1	1.239106047	4.25E-09
VPS13B	1.237488095	4.91E-19
APH1B	1.237375849	1.18E-04
RP11-385F5.4	1.237018434	1.96E-03
CLIP4	1.236759971	2.77E-21
STRN3	1.235937979	5.10E-18
MTMR11	1.235620811	6.72E-17
EIF2S2P4	1.235236623	1.54E-13
NAPB	1.235187001	5.70E-15
AC069277.2	1.234030569	1.33E-03
BDP1	1.233628669	4.51E-24
RP11-111M22.2	1.233492122	4.14E-05
TCEANC2	1.233408027	1.44E-13
CTC-281F24.5	1.232932555	1.59E-04
PGAM1P7	1.232233949	2.16E-04
PKP4	1.232050269	2.49E-25
RP11-49K24.8	1.232042028	2.69E-04
MSRB3	1.23150823	7.16E-12
TOPBP1	1.230990001	1.34E-24
TMOD2	1.229330649	3.91E-06
AC099850.1	1.228699943	4.96E-09
RP11-849H4.2	1.228635178	3.66E-05

MBNL1-AS1	1.22668633	6.14E-07
ALG1L13P	1.22658522	1.66E-04
ZNF397	1.226356381	8.14E-19
TBC1D3I	1.226322801	4.01E-05
KIAA0907	1.225698513	6.13E-21
RP11-347C12.11	1.224953141	2.96E-03
LINC00893	1.224799852	1.74E-05
IGFL1P1	1.224772168	7.10E-07
RP11-727A23.5	1.223058602	4.05E-10
RP11-477I4.4	1.22123191	1.31E-03
ARL17B	1.220382808	3.83E-08
RP11-680F8.1	1.219842401	4.00E-11
AC005042.4	1.21972207	8.78E-11
ZNF514	1.219491668	1.25E-06
FAM227A	1.218599922	2.08E-04
IFT122	1.218448365	7.81E-20
SUPT16HP1	1.218020996	4.84E-16
ZBED6	1.218017342	9.71E-15
ENTPD4	1.217545265	4.61E-16
IL17RB	1.217005482	6.80E-11
ZNF678	1.216995707	1.21E-04
PAXBP1-AS1	1.216981686	4.02E-03
AGAP12P	1.216434839	2.73E-03
CEP152	1.215953354	7.51E-20
FZD4	1.215005278	3.06E-16
PDGFB	1.214789467	2.68E-07
ARHGEF6	1.21472799	1.44E-12
CES3	1.21466336	3.53E-07
LINC00483	1.213672818	1.09E-06
SETD5	1.213192327	1.71E-24
AKAP11	1.213018577	4.60E-17
TOP2A	1.211725074	9.29E-28
ADAL	1.211483798	2.08E-06
HNRNPH1P1	1.211194978	6.74E-03
RHOBTB1	1.208617583	1.92E-17
AC007566.10	1.208152515	1.53E-03
PRDM1	1.207602411	9.63E-10
AL133493.2	1.207242812	1.06E-03
AKAP7	1.206720406	4.30E-09
FGGY	1.206140728	1.44E-06
AC012358.8	1.204770125	2.55E-06
DHTKD1	1.204567415	1.58E-15
PKP2	1.204465451	3.26E-21
AC073333.8	1.204375712	9.73E-07

SLTM	1.204345345	9.18E-28
BNIP3P4	1.204266866	2.25E-03
PCLO	1.203734026	1.04E-03
DNM1	1.20330395	6.13E-23
MMP25-AS1	1.203014014	5.25E-04
DPY19L2P2	1.202720237	3.73E-10
SLFN13	1.202691888	4.91E-21
HOXC6	1.20252063	5.17E-06
PYGL	1.202436893	1.17E-18
OR2A20P	1.202365016	9.90E-04
IKZF2	1.202209802	9.45E-04
RP1-261G23.7	1.200439253	1.39E-18
CEP95	1.199938141	1.49E-18
CLK1	1.199378741	5.18E-21
RP11-336K24.12	1.199260629	4.62E-03
ARHGAP11A	1.199185469	1.46E-26
CD8A	1.198975406	1.92E-03
WDFY3-AS1	1.198108513	4.68E-03
COX10-AS1	1.197484691	1.02E-09
MST1	1.197330125	8.66E-05
NES	1.197086592	2.43E-27
TAZ	1.197019355	4.22E-14
KIAA1257	1.196699137	4.17E-04
ZSCAN5A	1.196646619	8.48E-06
CMB9-55A18.1	1.196528028	1.32E-23
HOXC8	1.195939191	7.07E-05
MTX3	1.195487662	3.22E-10
FAM72B	1.195211412	2.23E-12
RP11-723O4.6	1.191920659	4.97E-04
ZNF407	1.191759712	1.73E-13
SFXN2	1.190764285	2.71E-05
NEMP2	1.188903262	5.02E-11
PHF20L1	1.188581927	3.63E-25
POU2AF1	1.187991943	8.60E-11
SHPRH	1.187803014	1.12E-13
LINC00115	1.187710771	6.31E-04
CHD1L	1.187126412	1.20E-27
AC137932.5	1.186986847	3.95E-03
PVT1	1.186668139	8.54E-19
CACNA1D	1.186063699	1.77E-04
CCAR1	1.185890397	1.44E-26
SLC15A1	1.183971759	3.79E-03
DDHD2	1.183923172	8.77E-18
FAM135A	1.183878437	5.42E-13

LOXL4	1.183609589	1.79E-03
URI1	1.182890934	7.75E-25
PITPNM3	1.182590796	1.24E-04
RP11-74E22.5	1.181346227	6.00E-04
RP11-406H23.2	1.181275461	1.19E-04
LRBA	1.18082999	3.06E-21
NCAPD3	1.180002056	2.04E-27
RP11-144G6.12	1.179734111	1.28E-03
DST	1.17963765	1.56E-27
EIF2S2P3	1.179536781	1.25E-04
NSD1	1.178279014	1.57E-21
ZNF236	1.177971203	5.22E-19
CA11	1.177835986	2.74E-11
RP1-122P22.2	1.177678581	3.04E-05
CTC-428H11.2	1.176859897	1.62E-04
EIF4G3	1.176810702	1.43E-24
MTCO3P9	1.176616886	1.34E-03
AC093818.1	1.17579071	8.82E-10
PPP1R12B	1.175513265	2.47E-16
GCNT4	1.175162437	2.03E-04
RP1-102E24.8	1.174069976	5.74E-03
KIF4A	1.173715085	6.55E-27
FBF1	1.173445122	1.24E-17
RP11-45M22.2	1.173344999	3.82E-06
RP11-337C18.10	1.171921342	1.60E-12
RP11-178H8.7	1.170861533	3.56E-09
AC005363.9	1.170123229	2.86E-03
ROBO1	1.169722948	3.98E-28
LINC01123	1.169621963	2.98E-11
RPGR	1.16955992	1.21E-11
TEAD1	1.168559256	2.94E-20
FAM81A	1.168510669	1.13E-14
FAM86B3P	1.168125639	1.23E-05
C18orf54	1.167807842	1.15E-07
ARL4C	1.16768109	2.30E-20
LL22NC03-N14H11.1	1.166529042	5.35E-04
FRG1JP	1.165369478	9.87E-04
SMG1	1.165290398	5.08E-25
ZC3H11A	1.16513908	3.70E-25
DDX3X	1.163705234	9.71E-29
MMP25	1.162930174	1.93E-03
AP4E1	1.162337354	3.66E-14
RGL4	1.161335347	2.13E-06
ANKRD24	1.161048124	4.60E-10

MTCO1P2	1.160961535	8.08E-06
MAP4K5	1.159514165	1.55E-15
HCG27	1.159059133	4.60E-03
MBTD1	1.158806007	5.76E-13
THOC2	1.158392508	6.36E-27
RP11-221J22.2	1.158088637	3.54E-03
AL162759.1	1.157791891	1.60E-04
LDLR	1.157336361	2.64E-25
GGT7	1.156983582	9.42E-08
CCDC183	1.156589985	5.76E-15
RGPD5	1.15633116	2.80E-14
FAT4	1.155965875	4.87E-03
AC006978.6	1.155957317	4.93E-05
FBXO9	1.15574947	3.03E-16
ALS2CL	1.155402777	8.06E-11
SMC4	1.155034918	1.36E-25
ADAT1	1.154739733	7.24E-14
USP24	1.15464019	2.96E-23
RP11-98G7.1	1.154631863	3.88E-04
GEN1	1.154493847	1.12E-05
OPA1	1.154221757	4.67E-23
MLXIPL	1.15390693	1.96E-08
USP37	1.153900681	6.80E-16
RGPD4	1.153894134	5.30E-06
MEI4	1.153864743	2.27E-03
SOS2	1.152848718	1.30E-10
AC079466.1	1.152688267	6.85E-27
MLK7-AS1	1.150144789	4.89E-07
ACADSB	1.149427895	1.70E-11
KIF24	1.149131278	4.77E-16
PDE5A	1.147181982	2.68E-03
LINC00467	1.14665954	1.83E-09
IL31RA	1.146371033	5.29E-04
CCDC136	1.146287269	2.25E-13
BAZ2B	1.145282494	2.74E-17
PLCB1	1.144312191	3.40E-23
CLCN6	1.143841983	2.32E-11
AC007191.4	1.143787338	6.57E-07
RP11-631N16.2	1.143614185	3.75E-04
RP11-326K13.4	1.143541153	5.78E-04
TGFB2-AS1	1.143530117	1.81E-16
EVI5L	1.143319926	4.97E-18
ZC3H11B	1.14222662	5.39E-09
MYOM1	1.141815529	1.60E-05

RP11-421L21.3	1.141747933	5.02E-04
AC007193.6	1.140508626	2.06E-05
ITPR2	1.140125188	3.99E-07
NRG1	1.139948927	1.71E-16
SULT1C2	1.138875856	1.03E-24
DLG1	1.138817778	4.66E-23
EZH2	1.138487571	5.43E-21
RP11-44F14.7	1.138041766	5.96E-08
SLC38A11	1.137934871	8.15E-05
CHD5	1.137613307	6.01E-05
NRIP2	1.137011656	2.06E-03
LARS	1.135884575	2.40E-23
BIRC6	1.135760935	2.90E-22
ORC2	1.135377724	1.99E-16
ZBED3-AS1	1.135164718	1.65E-03
ACSS3	1.135021693	2.61E-16
RP11-126O1.5	1.134787742	4.57E-04
NAA16	1.134167591	1.63E-09
ARHGAP12	1.133940849	5.73E-21
NBPF9	1.13361868	3.13E-19
SGSM2	1.133327567	1.96E-19
PECAM1	1.133216058	2.07E-11
RP11-96H19.1	1.132434256	5.07E-04
KLF9	1.131946178	1.63E-09
FBXO36	1.131654231	3.18E-05
NEU4	1.131378235	2.16E-04
SH3BGR	1.130880087	2.50E-07
HDHD2	1.129777011	4.37E-14
ROCK2	1.129397604	5.52E-19
DMXL1	1.128624549	2.21E-09
SREK1	1.128508064	6.51E-24
AHI1	1.128104306	6.67E-14
RP11-337C18.8	1.12574478	1.01E-07
RP11-700F16.3	1.124711366	9.36E-08
ZFP62	1.124677424	7.66E-13
MIA-RAB4B	1.124571807	5.83E-06
KDM6A	1.12455061	6.37E-17
SPATA25	1.123913818	6.33E-03
RP3-430N8.10	1.123770176	6.64E-04
ADAM12	1.123549269	4.20E-10
GTF2IRD2	1.123099413	8.49E-04
ST3GAL4-AS1	1.122608481	5.31E-06
FAT1	1.121712358	1.84E-27
NEB	1.121331006	3.25E-04

VSIG10L	1.121241504	1.43E-16
CLK4	1.120570551	2.43E-12
FLVCR1-AS1	1.119637733	3.88E-06
PTAR1	1.11952784	5.62E-18
OPA1-AS1	1.119242421	1.35E-07
LACE1	1.119114719	1.28E-03
PROCA1	1.118489211	7.49E-06
PCM1	1.118007685	6.28E-24
TUG1	1.116770255	1.70E-19
SNED1	1.116746259	1.81E-06
S100PBP	1.116488239	1.93E-14
IQCD	1.114708321	1.55E-10
RP11-61J19.5	1.11373725	3.39E-03
RASA1	1.113270105	3.10E-13
CUTC	1.113143657	3.67E-18
RFC1	1.113109626	3.31E-21
RP11-373L24.1	1.112387477	1.21E-04
CKMT2-AS1	1.11220962	1.12E-08
LINC00261	1.11219888	5.90E-11
ALG1L6P	1.111208024	1.04E-05
RP11-81A1.6	1.111056138	4.91E-03
ACKR3	1.110542614	1.19E-06
AP000357.4	1.110532007	3.35E-03
MALT1	1.109483425	1.64E-16
ARAP1-AS2	1.109448452	6.12E-04
SAMD15	1.109256329	2.61E-07
DDIT4	1.106554364	4.16E-25
RBL2	1.106504147	8.18E-17
SPDYE1	1.105529497	5.76E-03
IMPACT	1.105365882	6.81E-16
IARS	1.105194913	5.53E-26
GPC6	1.104606929	1.49E-09
CEP290	1.104128053	2.43E-16
NEAT1_1	1.103926886	4.46E-06
ROR1	1.103693329	9.03E-17
NAB1	1.10369029	1.16E-19
EYA4	1.103086343	6.08E-14
TAF2	1.10239456	1.52E-19
DDX60L	1.102055061	1.20E-12
PSIP1	1.10166695	2.02E-19
ACOT11	1.100914697	2.90E-05
CTD-2616J11.16	1.100219351	3.45E-06
AC090094.1	1.099995994	1.28E-28
BCHE	1.099855881	4.40E-03

GUSBP2	1.099199097	4.96E-03
RP11-449J21.3	1.099143698	2.47E-05
DOCK4	1.098980517	2.17E-19
HP1BP3	1.097781132	1.89E-21
DICER1	1.09752536	1.35E-19
CTD-2292P10.4	1.097508513	4.02E-03
PGBD4	1.09655171	1.12E-04
AC240274.1	1.096395342	2.61E-16
CCDC186	1.096242796	4.83E-17
SPEF2	1.096178796	5.77E-04
NNT-AS1	1.095717422	5.98E-09
ZNF638	1.095556243	7.89E-24
GHRLOS	1.095073209	8.44E-03
CTD-2651B20.3	1.094090624	7.04E-07
LTBP1	1.093417435	4.33E-21
ZNF846	1.093158854	1.62E-03
RP11-723O4.2	1.092927257	1.04E-03
RGPD2	1.092803554	6.48E-11
SNX25P1	1.091844799	3.97E-03
LMBRD2	1.091816064	4.67E-11
DOCK11P1	1.091791337	3.51E-03
ARHGAP22	1.091703478	4.21E-04
EGFR	1.090110204	1.09E-22
NRXN2	1.089975902	8.89E-12
ZBTB26	1.089742622	8.39E-07
ARID5B	1.088788948	4.75E-20
TMEM17	1.088567474	4.10E-03
RP11-582E3.4	1.08780164	1.99E-03
VEGFA	1.087272406	7.86E-24
SIRT5	1.08721758	5.46E-09
RP11-278C7.5	1.08706662	3.27E-06
HDAC9	1.086956518	8.50E-19
CTD-3199J23.6	1.086490915	1.06E-03
RAD50	1.086248859	5.73E-20
DET1	1.086233069	1.18E-06
CDK5RAP2	1.083794684	1.53E-25
AF127936.7	1.083615909	8.06E-04
AC013461.1	1.083106283	3.38E-19
CLDN2	1.082483066	7.68E-11
PCNX2	1.08098876	9.01E-11
TBC1D3F	1.080926705	4.14E-03
C8orf44	1.080906999	3.92E-04
PLXNB3	1.080366332	2.57E-11
SLC28A2	1.080046818	2.96E-20

STK36	1.080037265	9.73E-07
PGAP1	1.078712715	9.75E-06
MTRF1	1.077756819	1.63E-05
LINC01560	1.077306627	3.30E-03
GOLGA6L10	1.077170979	1.22E-06
SPATA6	1.076533134	9.01E-05
RP11-582E3.6	1.07636524	1.08E-03
REEP2	1.076081781	2.37E-03
CCDC68	1.075829002	9.62E-05
PCOLCE2	1.07569491	2.42E-12
SLC5A3	1.075558204	2.43E-21
ASPH	1.07554374	1.97E-28
GAS8	1.075455547	4.39E-20
COPG2	1.075337293	1.89E-11
MAN2C1	1.07440998	2.92E-13
DDX17	1.072713778	2.07E-24
CHD1	1.072481717	2.76E-21
VPS26B	1.072325018	6.10E-23
AC002066.1	1.071170813	1.35E-08
RC3H2	1.070440126	4.81E-20
RP11-403F21.4	1.070364966	1.77E-04
RTTN	1.070258098	1.45E-08
AC004951.6	1.068991422	1.11E-03
ARMC2	1.068715318	1.01E-03
ANKRD29	1.068619964	9.40E-15
ANKRD6	1.068032088	5.11E-13
STAG3L5P	1.067792718	1.24E-14
EME1	1.067585561	2.16E-14
LTBP2	1.067536868	1.85E-03
ABCC6	1.067134534	2.86E-05
POLI	1.066662102	6.42E-07
LINC00601	1.06615554	2.23E-03
RCCD1	1.065974573	4.32E-16
BTBD19	1.065917825	8.04E-04
DOCK5	1.065584296	1.17E-19
ZNF252P	1.065292481	5.86E-17
ATP6V1E2	1.065179742	1.89E-07
UPF2	1.064930197	1.91E-19
SLC25A36	1.063952947	6.88E-09
MDM1	1.063817083	3.33E-14
RP11-568K15.1	1.063207685	3.00E-07
IQCG	1.06302046	1.84E-10
KRBA2	1.062365831	1.91E-04
TRIP11	1.062238191	4.51E-18

CPEB3	1.061284141	6.52E-03
HLTF-AS1	1.060963202	5.19E-03
SYDE2	1.060313497	8.60E-03
TPM3P9	1.060153875	2.25E-08
AC006369.2	1.0600383	1.50E-03
ZFP91-CNTF	1.059888793	1.25E-24
MORC4	1.059356613	2.87E-25
ATG16L2	1.059277282	1.16E-10
MB21D2	1.058938608	7.75E-06
SUGCT	1.058563393	4.90E-04
SLFN11	1.057079128	1.80E-04
VPS54	1.056660117	2.47E-14
F8	1.056467778	7.09E-05
ZNF780B	1.056462889	6.83E-09
DPYSL4	1.056319438	1.79E-03
LINC00969	1.056304658	2.77E-18
GCC2	1.056204167	2.50E-19
DDX12P	1.056003084	1.24E-12
AC008982.2	1.055787997	6.11E-03
PPP1R9A	1.055761089	6.82E-17
GMDS-AS1	1.055074744	9.19E-03
PRKG2	1.054792287	3.13E-03
RP11-299G20.5	1.054647884	4.10E-03
CCDC148	1.054596398	3.91E-03
MAP3K7	1.054454099	1.25E-15
HIPK2	1.054123778	3.80E-23
RAI14	1.053880156	1.55E-24
LYRM7	1.053511084	1.77E-08
ANTXR2	1.051840784	2.49E-21
RP1-152L7.5	1.051604998	1.90E-11
TMPRSS9	1.051132713	4.41E-03
RP11-561O4.1	1.050998753	4.97E-03
MAPT	1.050844479	1.31E-14
LINC01446	1.050810253	4.78E-05
TSPYL4	1.050711545	2.72E-09
SERTAD2	1.050580006	1.05E-19
JMJD1C	1.05049015	5.52E-19
IGF2BP3	1.050081126	1.83E-19
BUB1	1.04968339	2.14E-21
RP13-131K19.2	1.049232492	1.43E-05
CENPI	1.04911928	3.71E-09
RASSF6	1.048406241	1.22E-08
PCSK9	1.048257789	1.45E-20
CH17-232I21.1	1.048010842	3.42E-04

LINC01004	1.047337082	7.87E-03
KANSL1L	1.047169355	9.11E-06
PREPL	1.047152213	4.43E-16
ANKRD20A5P	1.046958965	2.32E-06
RP11-296I10.3	1.046740564	5.22E-05
LUC7L3	1.046549682	7.42E-26
RP11-442H21.2	1.045838768	3.49E-22
ZNF469	1.045780141	8.22E-17
MTCO1P40	1.045717204	1.05E-15
RP11-342M1.3	1.045504522	1.18E-03
VIM-AS1	1.045219495	1.25E-03
ATP5S	1.043054368	1.07E-07
CTD-3088G3.8	1.042483494	1.65E-03
SNORA40	1.042012602	1.44E-03
SGK494	1.041712857	2.64E-07
CRMP1	1.0416094	1.14E-07
SUPT16H	1.041111686	1.21E-25
RP11-884K10.7	1.040804179	1.54E-03
RP11-388P9.2	1.040388314	1.17E-06
NEURL1B	1.040162028	8.33E-08
ZNF37A	1.039542644	6.07E-13
RP5-1185I7.1	1.038680487	6.17E-06
EBLN3	1.038667079	1.96E-17
ATAD2B	1.038310898	2.09E-06
ZFX	1.03828742	1.55E-11
SENP6	1.037435467	6.18E-19
IPO9	1.037061882	2.51E-22
NBPF11	1.037032357	8.74E-14
WHRN	1.036887851	1.47E-08
ATRX	1.036829146	3.16E-23
TCTN2	1.036002907	3.97E-08
MGA	1.036000697	3.39E-19
GK	1.035968154	9.59E-06
C16orf86	1.035625054	2.77E-03
ZNF107	1.035236148	2.92E-06
MBTPS2	1.035152209	4.80E-11
DBT	1.034957667	1.33E-09
PHIP	1.034935908	7.46E-20
CEBPG	1.034704381	1.85E-21
TMLHE	1.034676454	8.81E-10
TNRC6C-AS1	1.034521871	8.44E-03
DOCK11	1.034360308	4.28E-19
BRE	1.034129887	9.80E-10
RP11-432B6.3	1.033970482	1.69E-10

RP13-638C3.4	1.033931041	3.29E-10
TRAPPC11	1.033713583	3.23E-14
TRAPPC9	1.032422184	3.17E-14
MED12	1.032311667	7.46E-21
ABCC4	1.032198168	2.06E-12
KIAA0753	1.031857962	5.05E-13
STYXL1	1.030796067	3.29E-15
RAPGEF2	1.03040131	6.24E-19
LOX	1.03038554	4.12E-06
RP11-618N24.1	1.029903956	1.02E-02
MNS1	1.029805022	1.84E-06
LRRC37A2	1.029745311	3.45E-04
GABPB2	1.029174506	5.64E-17
RGPD3	1.028340195	1.33E-05
OTUD3	1.027688802	1.03E-11
FAM78A	1.027579809	3.72E-03
YY2	1.027175258	1.07E-03
KRT86	1.026827955	8.50E-03
PIKFYVE	1.025597685	4.19E-14
ZNF404	1.025548345	4.14E-03
SLC26A9	1.025404072	4.86E-06
RP11-299G20.2	1.025313162	1.09E-04
TCF19	1.025299552	7.66E-18
STX2	1.024945296	1.94E-16
MSH3	1.024564228	5.07E-12
FAM86C2P	1.024416852	8.08E-07
RP11-192H23.4	1.023998625	1.49E-18
AC006460.2	1.023453722	2.71E-11
STARD4-AS1	1.023353022	8.72E-05
CYB5R2	1.023259133	1.14E-03
BPTF	1.022780867	3.57E-22
RP13-104F24.2	1.022628183	1.60E-14
RP11-856M7.2	1.022593495	2.48E-04
TLK2	1.022475189	9.49E-20
ARHGAP19	1.022408306	6.04E-10
RP11-288H12.3	1.022399252	7.38E-03
RP11-849F2.5	1.022249791	4.69E-03
CHDH	1.022175597	1.06E-13
ZNF605	1.022054091	5.02E-19
AL135791.1	1.021216449	8.99E-04
RP11-307N16.6	1.020747374	5.90E-13
CH507-338C24.1	1.020481858	3.13E-10
NBEAL2	1.019602624	5.46E-16
OBFC1	1.019391867	4.40E-18

RP11-516C1.1	1.01887175	5.12E-13
WAC-AS1	1.018526411	1.75E-12
DMGDH	1.018451138	1.46E-04
YOD1	1.01834385	2.29E-14
KIF18B	1.018133912	1.17E-17
INPP4B	1.018035066	7.85E-03
DLG2	1.01775493	1.58E-02
HLCS	1.017740393	4.17E-15
RP11-103J8.1	1.017634229	1.96E-07
RP11-727A23.10	1.01702843	7.59E-03
NINL	1.016894831	5.05E-20
AASS	1.016498789	4.39E-05
ZNF292	1.015744429	1.04E-15
ARL13B	1.015430531	1.52E-16
RP11-158L12.4	1.015372276	1.05E-03
CCHCR1	1.015007873	3.18E-15
INTU	1.01384546	5.03E-06
GATM	1.013793265	3.22E-12
CYP4V2	1.013771004	1.32E-06
B3GALNT2	1.013581011	4.55E-12
FAM167A	1.013067979	9.68E-03
SYTL3	1.012111349	9.21E-04
SPAG5	1.012041072	2.74E-24
ARHGAP18	1.011244375	3.77E-19
RP11-774D14.1	1.010961485	1.84E-03
ERFE	1.010489608	4.31E-04
RP13-631K18.2	1.009745736	4.46E-03
ALOX12P2	1.009494802	6.35E-06
RARRES1	1.009073948	9.17E-10
DCDC1	1.008223345	3.04E-03
CHEK2	1.008041748	4.79E-10
F10	1.007314536	3.06E-03
NR3C1	1.007296532	4.59E-14
CH17-431G21.1	1.007228226	1.10E-06
ABCD3	1.007223766	1.01E-18
WDR11	1.007046246	1.89E-13
AC018892.9	1.007033657	4.74E-03
INO80	1.006931362	6.10E-19
TRIT1	1.005917442	6.71E-12
TUBGCP5	1.005441531	1.29E-10
PTGR2	1.004539171	1.16E-10
PIEZO2	1.004510848	2.45E-20
PTPDC1	1.004446503	4.48E-11
ZMYM4	1.004320417	2.92E-17

FAM117B	1.003500206	1.27E-06
IL1RL2	1.003476695	2.25E-05
RP4-639F20.1	1.003238494	6.46E-03
LRRC27	1.00292503	6.35E-07
TTC6	1.002703625	4.26E-03
SEMA3E	1.002646232	1.52E-03
ASAP1	1.002264182	1.40E-23
MAP3K5	1.001790901	2.02E-12
TRAK2	1.001782398	9.20E-14
ALG10B	1.001436331	5.67E-06
MORN3	1.001307448	3.62E-03
AHCTF1	1.00110674	2.62E-22
KATNBL1	-1.001368559	4.92E-08
AC090498.1	-1.001769044	1.27E-24
MXD4	-1.00285321	2.15E-19
GTF2B	-1.003195765	2.59E-16
HAPLN3	-1.00322653	1.95E-04
FUS	-1.003441303	9.11E-25
KTI12	-1.003978963	4.82E-13
PRKAB1	-1.004405188	4.18E-17
TRIM31	-1.00511346	1.17E-23
BOK	-1.005588968	6.35E-19
C16orf91	-1.00651923	1.46E-13
EGR2	-1.006886777	1.16E-03
IL7	-1.007049136	9.05E-09
RP13-279N23.2	-1.007318205	7.66E-19
SNX11	-1.007422822	3.25E-10
FGL1	-1.007627977	2.68E-16
FOSB	-1.008142209	2.97E-03
POMP	-1.00846741	1.73E-17
COMMD5	-1.009719266	7.64E-20
KYNU	-1.009822093	7.69E-21
GAPDHP75	-1.010427146	9.31E-03
HSPE1-MOB4	-1.010946011	1.08E-24
U2AF1L5	-1.010985128	1.18E-19
RP11-665C16.9	-1.011281794	1.23E-03
SP110	-1.011342639	9.41E-13
UBALD1	-1.011423986	6.69E-15
C3orf38	-1.012219958	2.40E-13
RBM43	-1.012222286	4.85E-05
ZNF823	-1.012375924	1.53E-07
HSD17B11	-1.013931045	1.20E-17
STARD10	-1.014429918	6.38E-19
CD2BP2	-1.015824507	3.06E-23

MRPL36	-1.016498008	2.01E-18
RP11-122G18.7	-1.016954027	5.11E-03
RPS13P2	-1.017003446	2.21E-04
CTD-2302E22.2	-1.018059677	4.56E-06
PRKCD	-1.018588274	3.86E-21
RP11-464D20.2	-1.018915695	2.52E-03
TXN	-1.019761402	3.31E-25
RNASEK	-1.02029662	1.00E-18
UBE2L6	-1.02064085	1.27E-13
RP11-296A18.5	-1.022094155	8.58E-06
RNF31	-1.023361386	2.73E-17
CCNJL	-1.023461615	1.85E-13
FA2H	-1.024025666	5.43E-06
EVA1B	-1.024375284	5.41E-13
RP11-169K16.8	-1.024467324	3.66E-04
NFYB	-1.024488277	1.96E-20
SDCBPP2	-1.024496147	5.02E-03
ANXA2R	-1.025083173	1.75E-03
ATP6V1D	-1.025483105	2.15E-18
SLC26A1	-1.025565893	6.24E-07
USMG5	-1.025759006	1.19E-15
BCL3	-1.026448086	1.38E-18
HSPE1	-1.026968714	5.83E-25
RP11-12M9.3	-1.02756443	1.28E-17
RP13-514E23.2	-1.028940018	1.76E-03
RRAGC	-1.029416013	1.44E-18
TMSB4XP2	-1.029685116	3.16E-03
NDUFAF4	-1.031774708	3.55E-12
RHOF	-1.033479799	1.66E-18
FAS	-1.033897855	1.51E-03
BCORL1	-1.034213736	7.24E-20
PAOX	-1.034315998	1.32E-04
PLIN3	-1.034714652	8.27E-24
RBM14-RBM4	-1.034740279	2.93E-23
AC011530.4	-1.035162931	4.47E-12
RP11-732A19.9	-1.035918627	2.36E-10
CTC-518P12.6	-1.036259056	2.01E-14
FOXD2	-1.037265687	1.66E-05
KRT8P10	-1.037683537	1.59E-10
HSPE1P9	-1.037865772	1.21E-04
AC005102.1	-1.037919665	2.70E-03
RP11-1035H13.3	-1.038037505	1.85E-26
PELI3	-1.03865558	8.17E-18
RP11-156E6.1	-1.038818693	1.29E-03

DMWD	-1.040668851	4.52E-17
RTN2	-1.041604151	8.80E-08
PNP	-1.042257378	2.34E-24
AC009950.1	-1.04249157	5.47E-13
MAST3	-1.04295668	2.73E-13
ZNF319	-1.043108415	7.04E-09
CTD-2012K14.2	-1.043547029	9.04E-10
MEGF6	-1.043605428	6.71E-03
KRT8P50	-1.043656391	2.56E-08
HSPA2	-1.044358932	3.28E-09
TEAD4	-1.04448978	1.32E-13
PPP1R3D	-1.044573189	1.42E-03
MSX1	-1.045319712	2.55E-13
RPS12	-1.046420058	1.48E-26
TPP1	-1.048843269	1.20E-18
TBC1D10A	-1.048957127	4.13E-13
LAMB3	-1.049555805	1.01E-20
ZNF213	-1.04996836	3.53E-09
NFKB2	-1.051021399	2.62E-21
SYPL2	-1.051022259	1.39E-03
OVGP1	-1.051721633	1.92E-05
CACFD1	-1.051832949	3.01E-14
PLIN4	-1.05219147	1.57E-13
JHDM1D-AS1	-1.052262961	1.82E-03
CLDN7	-1.052271935	6.55E-06
RPS15AP38	-1.052608451	7.17E-03
ZNF547	-1.053251743	2.14E-04
SH3BGRL2	-1.055309696	8.92E-17
RP3-340B19.2	-1.055676549	1.20E-06
NPM1P33	-1.055951538	4.14E-03
TSSC4	-1.055998101	1.06E-17
RAB3IL1	-1.056684029	1.60E-19
RHBDD3	-1.057182801	2.27E-11
ISG15	-1.059114288	2.58E-19
MAFG-AS1	-1.059269201	3.50E-10
S100A11	-1.060006	2.02E-27
RPS27P15	-1.060257505	2.74E-05
RP11-864N7.2	-1.060481641	1.42E-11
MTRNR2L8	-1.060493956	1.06E-20
MGRN1	-1.061036427	2.04E-20
RP4-800G7.1	-1.061748618	1.76E-03
VPS37B	-1.06239631	5.21E-22
HIST1H2BJ	-1.062489813	1.72E-03
RPS9	-1.062866144	2.75E-26

AC027682.1	-1.063956544	6.88E-22
FAM65A	-1.064286001	4.07E-22
C8orf4	-1.066403933	5.82E-20
PRR5	-1.066427126	1.39E-07
TMEM177	-1.067532659	2.69E-09
IRF5	-1.06757923	3.64E-07
GPRIN3	-1.067795408	1.73E-07
ZNFX1-AS1_2	-1.067860665	5.86E-18
TSPAN31	-1.068224795	4.35E-25
RHEBL1	-1.06845397	6.31E-10
FZD1	-1.068530053	4.51E-10
RARA	-1.068782638	4.70E-18
RCAN1	-1.070168562	1.72E-12
TUFMP1	-1.070465731	2.85E-03
MPV17L2	-1.070486626	3.47E-11
NAV1	-1.070918598	8.03E-17
RP11-20I23.3	-1.071077859	5.01E-18
MT-TF	-1.071789999	5.08E-05
CTD-2215E18.1	-1.071990381	3.61E-04
ACVR1B	-1.07238074	3.50E-18
KCTD9P2	-1.07244887	4.08E-03
SPATA1	-1.072690508	1.92E-04
TM4SF20	-1.073109514	1.69E-03
TTLL12	-1.073214539	6.48E-24
RPS29	-1.075625544	4.29E-22
OSTC	-1.075673176	4.77E-20
C1orf226	-1.075751298	1.11E-14
TMEM99	-1.076132762	1.00E-03
HDX	-1.076659413	4.36E-08
U2AF1	-1.078904915	6.74E-25
TINAGL1	-1.079024836	9.86E-19
C19orf24	-1.080075809	4.27E-16
RPS15AP24	-1.080166451	1.49E-03
SYNPO	-1.080368953	5.80E-13
NCDN	-1.081821498	8.77E-17
ZNF771	-1.083819445	3.89E-12
ZBTB9	-1.084189722	7.27E-12
HPD	-1.084342466	1.11E-06
NOP10	-1.08489692	9.97E-22
RELB	-1.085359916	1.39E-14
ZSWIM4	-1.085915478	3.10E-11
ZNF622	-1.087917125	9.48E-24
NATD1	-1.089315485	3.70E-14
RPUSD1	-1.089665577	1.01E-17

ZNF408	-1.089785018	1.94E-13
AGAP1-IT1	-1.089858546	1.99E-03
SERPINI1	-1.089970397	6.43E-04
TMEM191C	-1.090186629	4.22E-04
DAPK3	-1.090617716	3.24E-22
COMMD6	-1.090687946	2.87E-10
RP11-132A1.4	-1.091238473	1.07E-19
EFNA3	-1.091360259	1.62E-11
SLC10A3	-1.091679207	1.10E-17
CISH	-1.09358585	1.50E-05
INTS5	-1.094865406	7.49E-14
KLHL25	-1.097214535	1.73E-10
GPX1P1	-1.098365734	2.26E-17
ATP6V0E1	-1.098827769	4.53E-24
GPX1	-1.100119199	2.78E-23
SLC22A18AS	-1.100840369	3.71E-11
LRFN1	-1.103048112	6.78E-04
NAT6	-1.103408684	4.34E-11
CCDC69	-1.103488163	1.29E-09
RASSF5	-1.104063877	2.02E-06
AC007182.6	-1.105368427	6.15E-04
CTB-52I2.7	-1.105555395	2.95E-04
C15orf62	-1.108206999	1.91E-04
PRAP1	-1.108449464	7.99E-07
RND3	-1.108450537	5.57E-23
PRKCDBP	-1.108546966	2.89E-19
AMER1	-1.109928656	4.96E-16
CCDC134	-1.110341015	8.46E-10
RP11-565P22.6	-1.111588698	3.83E-08
FOSL1	-1.111797045	1.00E-22
GAS2	-1.112310889	4.78E-06
CTB-33G10.1	-1.112696823	1.20E-10
TRIM68	-1.116158246	7.07E-17
CTSA	-1.116887518	1.96E-26
ANPEP	-1.119689755	8.75E-13
RSPH9	-1.119804172	8.81E-09
AC010761.10	-1.120759139	1.41E-05
TMEM120B	-1.120773949	5.59E-22
RP11-132N15.2	-1.121092636	2.96E-03
ACTRT3	-1.121336404	9.78E-04
SNPH	-1.123548445	2.50E-11
RBM14	-1.12385865	9.72E-21
PSMB9	-1.124632783	2.17E-08
CD63	-1.12463846	5.68E-28

RP11-388M20.6	-1.124702918	2.80E-17
TEX30	-1.124778353	5.06E-09
HIST1H2BD	-1.125435894	1.96E-12
TNFRSF1B	-1.125566672	4.21E-13
RIMBP3B	-1.125721583	4.27E-05
RNASEL	-1.127363863	1.67E-10
SERPINB8	-1.129072621	5.46E-08
RP11-247A12.2	-1.129899246	7.74E-16
CTD-2267D19.1	-1.130665941	3.62E-11
POLR2A	-1.130829804	5.30E-26
RP11-479G22.8	-1.131662696	1.31E-03
RP11-40C6.2	-1.134199902	2.03E-15
RP11-1E1.2	-1.134806017	2.15E-06
BICDL1	-1.136085711	1.49E-22
RP11-552F3.9	-1.1369305	7.94E-23
KCNF1	-1.137324059	3.49E-09
CTC-250I14.6	-1.137513236	2.62E-22
RPS15A	-1.138573918	1.74E-28
RP11-183G22.1	-1.138850751	6.89E-03
AC007228.9	-1.139175524	2.73E-04
SAMD10	-1.139723536	7.13E-08
RP11-214O1.2	-1.140892222	3.72E-03
TLCD1	-1.141456085	9.60E-13
TRIM6	-1.141824697	1.06E-11
CTD-2073O6.1	-1.14350205	4.91E-04
ATP6V0C	-1.143593565	5.76E-24
TRAFD1	-1.143838686	8.22E-23
RP11-540D14.8	-1.143927985	5.05E-10
DUOX2	-1.144230883	5.27E-04
LASP1	-1.144401742	8.73E-29
C14orf169	-1.145189929	1.69E-12
FAM214B	-1.145846916	2.54E-12
SECTM1	-1.146108689	1.01E-04
DBIL5P	-1.14666973	3.01E-03
C12orf49	-1.147572384	2.86E-23
PROSER2	-1.147978641	1.05E-13
RIPK2	-1.148199186	9.29E-22
RP11-20I23.1	-1.148357789	1.47E-23
MGAT1	-1.148615838	9.85E-22
RP11-203M5.8	-1.148729595	2.80E-22
IER5L	-1.149048581	8.13E-23
ZNF503	-1.149318952	1.10E-14
PIP4K2C	-1.150197477	1.19E-18
SMIM22	-1.150271051	1.01E-03

KCNK5	-1.150307864	5.38E-16
ZNFX1-AS1_3	-1.151336654	4.79E-21
COMMD9	-1.151437183	4.65E-14
BOK-AS1	-1.151633353	1.14E-08
GDPD1	-1.154024074	5.89E-04
RPS9P1	-1.154575388	2.14E-03
SRRM3	-1.155515922	7.25E-09
SPP1	-1.155872073	2.53E-28
AC016999.2	-1.156537057	2.47E-06
RP11-96D1.7	-1.156830464	6.41E-05
COPS9	-1.156967379	5.57E-13
TIGAR	-1.156999305	2.21E-16
TMEM102	-1.157172374	5.67E-10
TBCAP3	-1.157373061	1.71E-03
CARD16	-1.158350164	1.99E-06
C1orf122	-1.158509496	1.02E-18
MTRNR2L12	-1.161978124	3.75E-24
GJA3	-1.162788885	2.60E-04
TSPAN1	-1.163566092	6.85E-04
RP11-688G15.3	-1.163630934	1.45E-07
M6PR	-1.163913955	1.50E-26
TRIM47	-1.1654985	1.91E-25
ABHD13	-1.166016905	1.17E-10
PHLDA2	-1.166889984	2.72E-23
RP4-536B24.2	-1.167581402	2.02E-03
TMSB4XP4	-1.169938057	8.10E-06
RP11-903H12.3	-1.170173845	2.35E-15
SFN	-1.170487973	7.39E-27
RP11-154J22.1	-1.170825695	2.62E-06
SPRYD3	-1.17165281	2.90E-24
ABRACL	-1.171669703	1.42E-20
GPR137B	-1.174657414	3.51E-07
GAPDHP64	-1.175682617	2.02E-04
RP11-463O12.3	-1.177433465	2.35E-10
EEF1E1	-1.177465326	4.79E-13
C11orf31	-1.178005442	2.31E-22
DPH2	-1.178268003	1.28E-18
RP11-449L13.2	-1.178435212	9.47E-05
UBTD1	-1.17874524	1.12E-19
SLC35C1	-1.179396145	4.20E-19
RP11-761N21.2	-1.179597249	7.75E-10
HPS6	-1.182729423	1.04E-18
BCL9	-1.183226405	6.66E-21
TCEAL9	-1.184266424	1.17E-27

RP11-384O8.1	-1.184294191	2.21E-04
PGM5	-1.184835916	3.61E-15
RP11-307L3.2	-1.18661456	2.60E-03
TMEM265	-1.187024804	2.46E-05
NPM1P21	-1.187057891	8.64E-05
C12orf29	-1.189302652	2.07E-15
ADPRHL2	-1.189776297	1.36E-18
RPP25	-1.189949727	6.43E-24
RP11-146F11.1	-1.191404649	1.18E-04
MTX1P1	-1.191965005	2.31E-21
RP11-1094M14.1	-1.193591169	1.82E-04
CD3EAP	-1.195404648	3.56E-25
PML	-1.19565998	1.60E-20
BAK1	-1.19592688	8.39E-20
NOS1AP	-1.196639995	6.13E-15
IFITM2	-1.198056489	2.90E-08
SDC4	-1.199382225	1.10E-21
SLC9A5	-1.199421566	1.02E-04
RIN1	-1.201775781	4.46E-17
IFI6	-1.201786096	2.01E-08
MSRB1	-1.202660595	8.47E-22
GNG11	-1.203396922	5.28E-15
RP11-156K13.2	-1.204460154	2.63E-12
RASSF7	-1.204543851	1.08E-22
PFKFB4	-1.205201773	7.63E-17
MOB2	-1.20623588	4.06E-20
OGFR	-1.206710295	6.90E-28
HTR1D	-1.208567727	7.15E-04
KB-1639H6.2	-1.209619415	1.14E-05
EGFLAM	-1.212150854	8.10E-05
PIGR	-1.212314199	5.57E-05
SPINK1	-1.214409115	1.92E-20
SGPP2	-1.218995725	3.90E-11
GOLGA6L7P	-1.219537979	2.10E-03
UBFD1	-1.219717559	1.03E-21
VWA5B2	-1.224241789	4.25E-04
RP3-337O18.9	-1.224298312	8.03E-14
MT-TV	-1.225525203	2.51E-09
SRPRB	-1.227487726	1.44E-24
RP11-867G23.12	-1.228124059	5.52E-05
IL1B	-1.228453808	4.80E-07
ACSS1	-1.229679239	2.16E-17
APOL2	-1.229999905	1.62E-26
QRICH2	-1.230274735	2.18E-04

IFITM1	-1.23210123	9.67E-05
GOLGA6L2	-1.232656648	1.13E-08
UPK2	-1.233070475	9.02E-05
PLTP	-1.233531356	3.90E-17
RP11-304M2.5	-1.238249824	1.16E-09
AC068580.6	-1.238858921	4.72E-22
MDK	-1.241169374	2.44E-30
SNORD3A	-1.242434518	2.25E-03
GOLGA6L1	-1.244122592	4.21E-06
UBD	-1.246180973	3.33E-15
PRM2	-1.248743923	1.15E-04
RPL7P26	-1.248773476	3.43E-04
RP11-832N8.1	-1.249158638	3.09E-05
USP2	-1.251572372	2.51E-05
CHPF2	-1.252227463	3.73E-20
MAP1S	-1.253999638	1.32E-19
RNF144B	-1.254853979	4.84E-04
TMEM159	-1.256824278	1.48E-17
CBX6	-1.258700006	2.39E-24
RPL34P31	-1.26058155	1.03E-03
RP11-127I20.5	-1.260934024	5.27E-04
B3GNT8	-1.261503879	1.65E-03
VPS18	-1.262646546	3.37E-24
RP11-1348G14.1	-1.26485684	9.97E-06
PIP5KL1	-1.265914767	1.17E-03
GPR160	-1.266251357	1.48E-07
DHRS9	-1.266986418	6.21E-14
DUSP23	-1.267239781	8.71E-05
RBPMS2	-1.267466202	1.37E-10
DAZAP2P1	-1.267501681	1.32E-04
GPR180	-1.267584746	2.43E-07
BET1L	-1.268096405	1.29E-23
TMC8	-1.271204309	1.29E-04
CCRL2	-1.271785174	1.64E-07
PRICKLE3	-1.27307483	3.17E-23
RGCC	-1.273576476	1.17E-04
SNORD23	-1.274357213	5.52E-04
RPS26P31	-1.274760045	8.44E-05
CPTP	-1.275093431	2.72E-14
AC002075.4	-1.275684354	7.21E-05
BCYRN1	-1.276780244	4.43E-04
CTC-338M12.6	-1.27765493	6.55E-04
PHLDB3	-1.27863546	2.14E-24
RP11-247A12.8	-1.279391623	4.97E-06

MTERF1	-1.279660289	2.16E-13
TPRN	-1.281475757	4.82E-21
RPL34P18	-1.281909148	8.84E-07
FAM102A	-1.284256377	5.50E-30
RP11-1100L3.7	-1.288147081	1.31E-24
IL3RA	-1.290242135	8.26E-04
BACE2	-1.291493991	6.31E-17
CTSD	-1.291572574	1.01E-30
CDC42EP2	-1.291644602	7.61E-16
AC010980.2	-1.291964548	1.43E-08
KLF2	-1.29208716	1.63E-07
C9orf16	-1.292303119	6.12E-20
MFSD5	-1.292795779	6.91E-19
PRKCZ	-1.294443568	3.11E-04
AC012501.2	-1.295434793	1.76E-03
RP1-71H24.1	-1.298437094	4.94E-22
RP11-235E17.2	-1.298519557	9.83E-21
PKIB	-1.298941702	1.19E-19
ATP6V0B	-1.300821074	3.35E-22
ATG101	-1.302163365	1.50E-28
RGS1	-1.303259381	1.38E-04
ORM1	-1.303924704	1.51E-03
RP11-621K7.1	-1.304520219	3.36E-04
PLA1A	-1.305420268	2.94E-07
GLIS2	-1.311721502	9.44E-19
AMBP	-1.313543285	9.56E-08
OAS1	-1.313815975	1.46E-28
DBI	-1.31417843	2.87E-30
RP11-295K3.1	-1.319616462	2.90E-30
IRF1	-1.322459865	1.08E-21
RP1-56K13.3	-1.325613888	3.42E-17
RTP4	-1.326666308	7.96E-04
FAM155B	-1.327069545	1.78E-20
HIST1H2BC	-1.327942165	7.09E-05
METRNL	-1.332085867	7.01E-23
TNK1	-1.333141813	1.32E-18
ADGRF4	-1.334221388	1.49E-04
CLDN15	-1.334489126	1.46E-08
XKR8	-1.335472023	1.03E-10
ZIC2	-1.343610387	2.85E-05
SLC35E4	-1.343881984	6.22E-15
ANG	-1.344211974	2.44E-19
VPS37D	-1.347668879	1.66E-12
FAM89B	-1.351448269	6.24E-16

MAPK8IP2	-1.35217662	2.62E-13
IL18	-1.352644375	7.42E-13
SEC61G	-1.353459346	2.91E-26
SGK1	-1.357350713	1.76E-08
CX3CL1	-1.357827354	3.08E-05
MT-RNR2	-1.35969377	1.30E-33
GLUL	-1.360813522	1.97E-28
GRB7	-1.362822363	1.85E-09
TFF1	-1.363816223	5.46E-07
SLC6A12	-1.363820755	9.66E-04
AKR1B1P1	-1.36812728	1.48E-08
IRF7	-1.368835496	4.61E-14
APOE	-1.369204883	1.65E-10
AFP	-1.369574243	5.09E-05
NUDT18	-1.370873815	7.27E-14
EDA	-1.373008403	1.75E-15
H19_3	-1.37314225	5.37E-08
GABARAPL1	-1.374247508	1.06E-25
SH2B2	-1.378780356	1.48E-17
NOTCH1	-1.379263955	7.78E-20
WSB2	-1.380658447	8.71E-24
C1orf115	-1.380764306	2.05E-23
SPANXC	-1.38167174	1.02E-11
VDR	-1.382837863	1.17E-16
SP140	-1.383343584	8.31E-08
LINC01207	-1.38476772	7.33E-09
RPSAP14	-1.385278235	5.89E-04
CTD-3149D2.4	-1.388403827	2.25E-09
IL1RN	-1.389179451	1.10E-04
CFB	-1.389373838	3.57E-10
RP11-616M22.7	-1.390006272	5.35E-23
NRARP	-1.39004439	9.76E-08
ZYX	-1.391306859	1.12E-23
DUSP8	-1.393928697	3.58E-06
DACT3	-1.397569577	4.74E-05
HIST1H1C	-1.39770184	5.42E-20
MGST3	-1.398566042	6.89E-22
SHC2	-1.398938331	4.52E-04
MIEF2	-1.399219092	8.17E-16
CTD-2012K14.4	-1.400050471	3.24E-16
PIM2	-1.400719565	2.92E-16
ABHD4	-1.402671046	1.36E-26
FJX1	-1.402773167	9.65E-05
GJB3	-1.402823449	6.66E-06

HSPA6	-1.406354473	4.56E-04
RAMP1	-1.407692329	1.34E-22
PLK3	-1.4082085	4.09E-14
DEFB1	-1.408591599	6.22E-12
KLHDC8B	-1.410173646	3.30E-15
ABHD5	-1.410665563	3.90E-16
DDX58	-1.412971559	1.63E-12
RP11-295D4.1	-1.41989089	1.45E-10
C3orf14	-1.423900414	6.73E-05
RIMBP3C	-1.424527235	2.80E-04
SLC27A2	-1.425998797	1.57E-05
SPANXA2	-1.427849094	7.73E-21
PKMP4	-1.431139071	1.90E-05
MED21	-1.432423468	1.29E-21
SEC11C	-1.434931002	7.65E-24
KRT8P36	-1.438681552	2.67E-10
RHOB	-1.4426093	2.56E-15
TRAPPC2B	-1.442797361	3.62E-08
SPANXB1	-1.44284245	6.53E-17
CTD-2540B15.7	-1.442922244	4.33E-26
OR2I1P	-1.443082397	1.01E-04
XXyac-YM21GA2.4	-1.443437648	3.05E-05
LY96	-1.44427754	1.86E-07
TMCC2	-1.446172255	3.77E-06
GABARAPL3	-1.44626386	6.08E-04
FAM124A	-1.446418871	5.73E-04
ZNF385A	-1.446446803	5.33E-09
SCO2	-1.446465003	3.49E-16
MAP3K14	-1.447619252	6.16E-22
CSTA	-1.453782299	3.59E-07
RP11-483E23.2	-1.455506575	4.53E-04
ZNF296	-1.45633939	1.43E-09
AKR1B1	-1.458385512	1.32E-33
SPDEF	-1.45898199	6.62E-08
MOB3A	-1.459680347	1.04E-15
PIK3IP1	-1.463504814	5.07E-09
LRG1	-1.465241115	8.22E-06
EPHA1	-1.465344839	5.45E-11
AC069368.3	-1.465620823	6.07E-16
RPL37	-1.470751038	2.93E-32
PGF	-1.480949073	1.14E-12
RP11-407P2.1	-1.482078241	1.07E-04
EFNB1	-1.483253498	1.07E-29
MROH6	-1.486928994	1.24E-08

SPANXD	-1.488841826	2.18E-12
GOLGA6L22	-1.501248343	2.39E-05
CTD-2540B15.11	-1.502439629	1.10E-21
CEBPA	-1.503305653	2.83E-30
RP11-187E13.1	-1.504871107	1.24E-05
MMP7	-1.505516727	1.54E-30
SSUH2	-1.507752259	6.94E-08
OSTCP2	-1.508222317	2.14E-06
ADPRM	-1.511900092	9.45E-11
A2M	-1.515189236	1.37E-12
RAB11FIP4	-1.519085236	4.66E-21
KBTBD8	-1.521061231	5.67E-06
TGIF2-C20orf24	-1.523404082	1.96E-30
RP11-1275H24.3	-1.524048195	2.38E-05
NFKBIE	-1.524872067	9.26E-19
SPANXA1	-1.527700134	2.04E-20
HDHD3	-1.537309548	2.11E-22
CTD-2342J14.6	-1.539710282	5.06E-04
CTA-384D8.36	-1.541971417	4.45E-16
FHL3	-1.544471734	5.83E-08
SERTAD1	-1.544704942	2.77E-20
BHLHA15	-1.545270152	9.19E-05
C20orf24	-1.548739703	3.81E-31
RP11-445F12.1	-1.554669618	6.17E-08
FGA	-1.556905006	2.59E-17
RP11-290C10.1	-1.557374474	1.06E-05
RHOG	-1.562099884	2.30E-19
RP11-83J16.1	-1.563792295	1.56E-04
FOXC1	-1.56515543	7.48E-19
CTD-2270P14.5	-1.56711534	4.52E-04
GPNMB	-1.571784724	8.95E-20
KB-1125A3.12	-1.579946551	3.96E-04
CD14	-1.580865578	1.72E-05
ETV2	-1.586877842	6.92E-05
ZNF563	-1.589045551	9.14E-06
SYT13	-1.589412139	7.48E-17
AKR1B1P7	-1.589747488	1.74E-10
VNN1	-1.590926641	1.07E-26
RPS2P5	-1.591931307	8.59E-23
CDH6	-1.593293062	2.97E-25
CDKN1C	-1.593897736	3.25E-07
AC005786.7	-1.596241434	7.19E-11
TRIM62	-1.597679299	8.07E-17
SPATA2L	-1.597846845	7.17E-17

LHX1	-1.599734509	1.28E-05
ATP6V0D1	-1.599842377	5.45E-27
HID1	-1.607785656	5.58E-09
FLYWCH2	-1.62117726	1.76E-06
CRYAB	-1.624258034	5.63E-10
SHPK	-1.627707557	1.54E-24
PLAUR	-1.628736615	7.24E-21
TOR4A	-1.631816685	4.11E-20
PLEKHO2	-1.63304448	1.97E-22
PCDHB11	-1.633580763	2.38E-04
HIST1H2BK	-1.636295729	7.11E-17
ICOSLG	-1.640939721	5.50E-12
TIGD3	-1.641998588	2.02E-06
BATF2	-1.648812664	8.84E-16
RPL37P2	-1.649922385	4.39E-05
ADH1C	-1.654256696	4.10E-25
BIK	-1.655223365	2.19E-06
MFSD2A	-1.655663205	1.33E-12
SOX18	-1.657612674	1.74E-08
PTGES2-AS1	-1.657741228	1.15E-04
UFSP1	-1.662027719	1.18E-10
NR4A1	-1.665921633	4.55E-12
TRIM21	-1.668785478	2.98E-27
HIST1H4I	-1.676078649	5.52E-12
HIST1H1D	-1.680127935	1.56E-04
S1PR2	-1.683521134	3.57E-12
KCND1	-1.686695955	4.22E-05
HSPB8	-1.693585353	3.48E-26
DTX4	-1.697441859	1.76E-23
EFNA4	-1.69893058	3.41E-24
RND1	-1.6994167	2.39E-18
LA16c-60D12.2	-1.699422485	2.87E-08
SNCG	-1.707825342	5.41E-26
TJP3	-1.707839412	5.15E-17
CLEC2B	-1.713924661	1.49E-27
TP53INP2	-1.715151069	7.07E-24
HIST2H4A	-1.717314301	9.65E-21
DCN	-1.724251444	1.06E-05
RP11-277P12.6	-1.735885218	1.11E-08
BTBD10	-1.73748754	1.63E-21
PITHD1	-1.744857736	2.43E-22
RP13-977J11.2	-1.751014881	9.29E-07
RP11-203J24.8	-1.751910815	1.90E-21
WBP2	-1.755250697	7.28E-33

ACTL10	-1.757906486	4.97E-13
SMIM10L2B	-1.766495269	4.67E-05
MAMSTR	-1.768525601	8.55E-11
PTGES	-1.769889856	6.21E-18
RP11-465B22.3	-1.769983038	5.38E-10
CH507-9B2.1	-1.770606018	1.46E-07
RP4-669K10.8	-1.772006709	4.56E-05
H19_2	-1.780073117	1.62E-15
CTC-510F12.6	-1.781195428	1.38E-05
NMRAL1	-1.782362306	2.38E-13
NR1H4	-1.789221501	1.00E-13
FLG	-1.799709479	2.60E-07
MIR22HG	-1.809290315	7.34E-25
H19	-1.809536628	3.93E-32
FABP3	-1.817157607	9.66E-13
CALB2	-1.822578772	8.60E-27
RP11-152K4.2	-1.82546493	1.70E-05
FAM212A	-1.833339642	2.88E-08
GMFB	-1.836467416	1.65E-25
HSPE1P3	-1.839007078	1.59E-05
SERPINF2	-1.854999392	4.39E-05
GPR132	-1.867321144	1.12E-06
SPX	-1.87441601	1.80E-11
SLC22A20	-1.878638476	2.07E-06
SLC16A14	-1.881718414	1.65E-17
LFNG	-1.889857084	3.44E-12
WNT7B	-1.896380274	7.38E-15
FLG-AS1	-1.902095739	7.95E-07
UNC13A	-1.923844879	1.93E-06
CTD-2263F21.1	-1.925001278	5.76E-08
TBC1D2	-1.92840431	2.68E-18
H19_1	-1.947074055	1.57E-18
KCNE3	-1.952678154	7.98E-07
PRM1	-1.959186988	1.08E-09
FAM180A	-1.960271977	1.07E-05
CITED4	-1.963307236	1.14E-16
RP11-54H7.4	-1.97678716	2.27E-07
LINC01139	-1.994287539	1.90E-06
NCF2	-1.994701406	2.33E-06
HAP1	-2.000711638	2.02E-05
KLRC2	-2.001025508	2.78E-12
NEURL3	-2.003822967	7.95E-17
TMEM37	-2.006082509	3.12E-12
HES4	-2.007408639	5.03E-17

BTBD11	-2.015492592	1.78E-11
HYAL4	-2.019808418	2.67E-05
AP000580.1	-2.031174937	1.06E-08
AGT	-2.035492294	6.77E-16
SLA2	-2.042166514	6.04E-09
CCDC150P1	-2.046731311	4.92E-12
HIST1H2AC	-2.048094866	2.61E-20
MYCL	-2.050866581	7.16E-08
SLC16A11	-2.054469142	2.97E-06
ARL4D	-2.054805671	7.27E-21
RAB42	-2.058466518	2.17E-07
VASN	-2.081076272	1.64E-22
PAQR5	-2.096109335	2.47E-12
TMEM158	-2.106673816	6.10E-10
HES7	-2.118263861	1.24E-06
HES2	-2.119286078	3.14E-06
LCP1	-2.11935805	2.38E-09
THSD1	-2.126547681	1.53E-07
PDZK1IP1	-2.148567339	2.04E-06
RP11-1100L3.8	-2.154421242	4.41E-08
SLC51B	-2.16321468	1.88E-20
KRT4	-2.171668094	1.77E-05
FAM84B	-2.18526568	1.37E-09
TRIM72	-2.196741783	2.40E-20
PYCR1	-2.196836671	5.01E-08
LY9	-2.233278423	1.12E-05
RAB17	-2.243367613	1.54E-12
PDX1	-2.247414129	9.74E-07
USHBP1	-2.309733442	5.63E-13
FGG	-2.348182168	2.21E-09
C11orf86	-2.371859237	5.60E-24
RARRES2	-2.378601183	2.38E-33
AOC2	-2.39580144	3.59E-10
NOXO1	-2.406123419	9.58E-12
LCN2	-2.48257249	1.82E-31
CCDC152	-2.487418449	3.36E-18
RHOV	-2.503233516	1.11E-15
VGF	-2.553875596	5.19E-12
FGB	-2.570492543	2.89E-24
GIMAP2	-2.575794389	1.36E-07
TXNIP	-2.57684611	1.94E-14
SEPP1	-2.593006831	3.47E-22
NR4A3	-2.599098253	6.49E-07
SLPI	-2.610569014	5.97E-10

MYADML2	-2.624720195	5.14E-07
G0S2	-2.703362893	2.41E-36
RGS16	-2.770588063	1.31E-08
WFDC21P	-2.774756651	1.41E-07
BBOX1	-2.790096599	2.03E-14
IFITM10	-2.797802304	3.46E-37
SOWAHD	-2.826604746	5.46E-08
SP6	-2.842994135	6.82E-09
CD79A	-2.885874696	2.35E-19
RP11-297L17.2	-2.903478812	6.92E-10
CA14	-3.049230087	2.51E-08
ALX1	-3.051890644	2.39E-12
AOC3	-3.114978666	4.80E-08
IRF6	-3.115471329	1.39E-09
AC006538.1	-3.116795976	3.76E-13
PCDH1	-3.130089511	9.26E-13
MT1X	-3.211653836	1.16E-15
CLLU1OS	-3.238902379	8.36E-10
ALKBH5	-3.438509132	6.56E-41
SERPINB3	-3.509724218	2.81E-10
RP11-258F1.1	-3.515046887	3.51E-31
RP11-343H19.2	-3.680690316	5.30E-16
NKX3-1	-3.933523731	3.59E-10
MT2A	-4.257698887	1.24E-26
CD81	-4.717696428	2.03E-11
SNAI2[a)]	0.781082067	1.70E-03
CDH2[a)]	0.444309265	1.35E-09
ZEB1[a)]	0.903598316	2.08E-16
FN1[a)]	0.808028115	1.53E-22

[a)], EMT-related genes.

Supplementary Table 6. List of upregulated m^{6}A modification in LM3 cells with *ALKBH5* knockdown.

**Gene ID**	**Log$_2$ FC**	***P* value**
CXorf23	14.90688311	0.00E+00
MVK	14.44745149	0.00E+00
AC105760.2	14.44745149	0.00E+00
AE000661.37	14.25480641	0.00E+00
RP11-383C5.8	14.14789121	0.00E+00
GABRB3	13.76937958	0.00E+00
ADAMTS14	13.76937958	0.00E+00
FAT4	13.61737649	0.00E+00
ACVR2B	13.61737649	0.00E+00
SRGAP1	13.61737649	0.00E+00
GNAZ	13.57673451	0.00E+00
TET2	13.44745149	0.00E+00
RP11-84G21.1	13.44745149	0.00E+00
FXR1	13.44745149	0.00E+00
BRCA2	13.44745149	0.00E+00
TIGD4	13.35434208	0.00E+00
DUSP19	13.25480641	0.00E+00
WDR87	13.25480641	0.00E+00
RP1-244F24.1	13.25480641	0.00E+00
KCNC4	13.25480641	0.00E+00
ZNF862	13.25480641	0.00E+00
RFX7	13.25480641	0.00E+00
AIM1	13.14789121	0.00E+00
HOMER2	13.14789121	0.00E+00
PCSK6	13.14789121	0.00E+00
RANBP17	13.03241399	0.00E+00
RP11-293M10.6	13.03241399	0.00E+00
DZIP3	13.03241399	0.00E+00
HEMK1	13.03241399	0.00E+00
PMFBP1	13.03241399	0.00E+00
FARP1	13.03241399	0.00E+00
SEMA4F	12.90688311	0.00E+00
SH3RF2	12.90688311	0.00E+00
STX7	12.90688311	0.00E+00
COBLL1	12.90688311	0.00E+00
TMED3	12.86248899	0.00E+00
CTD-2530H12.4	12.76937958	0.00E+00
CUL2	12.76937958	0.00E+00
RP11-757F18.5	12.71048589	0.00E+00

CALU	12.61737649	0.00E+00
EXOC3L4	12.56292871	0.00E+00
PMFBP1	12.49184561	0.00E+00
LHFP	12.44745149	0.00E+00
PRRX1	12.44745149	0.00E+00
WSCD1	12.44745149	0.00E+00
SYNM	12.44745149	0.00E+00
LMLN	12.37345091	0.00E+00
RP1L1	12.35434208	0.00E+00
AKAP6	12.35434208	0.00E+00
HELLPAR	12.35434208	0.00E+00
PRDM1	12.35434208	0.00E+00
RBM44	12.25480641	0.00E+00
RGAG4	12.25480641	0.00E+00
FBXO22	12.18441708	0.00E+00
DIEXF	12.14789121	0.00E+00
PTCD3	12.14789121	0.00E+00
HOXA3	12.12552339	0.00E+00
HMCN1	12.12552339	0.00E+00
RP1L1	12.1104165	0.00E+00
HELLPAR	12.09130768	0.00E+00
HEMK1	12.09130768	0.00E+00
PPFIA4	12.03241399	0.00E+00
SLC9A4	12.03241399	0.00E+00
CFAP58	11.97101344	0.00E+00
APOB	11.94995183	0.00E+00
C10orf55	11.90688311	0.00E+00
HELLPAR	11.90688311	0.00E+00
DGUOK-AS1	11.8804109	0.00E+00
POU2AF1	11.76937958	0.00E+00
ACSS3	11.75848269	0.00E+00
RP1-179N16.6	11.72046998	0.00E+00
VCAM1	11.68845959	0.00E+00
CMYA5	11.64009657	0.00E+00
FAM50B	11.44745149	0.00E+00
FBXW10	11.44745149	0.00E+00
MSI2	11.44745149	0.00E+00
SNED1	11.44745149	0.00E+00
DNAH14	11.22505907	0.00E+00
PARVB	11.22505907	0.00E+00
SIGLEC10	11.18441708	0.00E+00
SLC8A1	11.17352019	0.00E+00
BCL2	11.17352019	0.00E+00
GNB1L	11.17352019	0.00E+00

KATNAL2	11.12552339	0.00E+00
KATNAL2	11.07548271	0.00E+00
PIEZO2	11.06358616	0.00E+00
MEF2C-AS1	11.06358616	0.00E+00
PRUNE2	11.06358616	0.00E+00
KIAA1328	11.06358616	0.00E+00
SH3TC2	10.94184995	0.00E+00
NREP	10.86248899	0.00E+00
DMXL2	10.86248899	0.00E+00
RSBN1	10.78194166	0.00E+00
RP11-47I22.4	10.78194166	0.00E+00
PLCE1	10.77407954	0.00E+00
WDR87	10.6871439	0.00E+00
DIEXF	10.60415454	0.00E+00
KDM7A	10.58975147	0.00E+00
CCDC171	10.52968046	0.00E+00
CCDC168	10.51890725	0.00E+00
RP11-134G8.7	10.49544828	0.00E+00
CYP2U1	10.44745149	0.00E+00
NELL2	10.20171708	0.00E+00
STOX2	10.20171708	0.00E+00
PPP2R2A	10.18911704	0.00E+00
PIK3R1	10.17352019	0.00E+00
LATS1	10.00433408	0.00E+00
LOX	9.986360646	0.00E+00
GOLGA7B	9.852922376	0.00E+00
SLC35A3	9.758482686	2.12E-305
RASSF6	9.71932431	6.78E-297
INTS6	9.630529955	3.56E-279
TMEM178A	9.515685751	1.47E-257
STARD6	9.111052837	0.00E+00
EFCAB6	8.89345933	0.00E+00
ITPR1	8.599454582	9.64E-136
MICALCL	8.469635996	7.89E-143
TRDN	7.982616015	2.35E-117
INHBA	7.887644867	1.98E-124
DNAH5	7.625686596	2.94E-179
CNGA1	6.916165809	1.90E-130
SPRY3	6.888197025	9.31E-143
DUOX1	6.598379197	2.48E-38
NTPCR	6.518228219	4.16E-53
MSRB2	6.50813911	2.99E-43
ARHGEF17	6.443208825	3.54E-66
FUT10	6.393496544	4.42E-89

FAM66C	6.366464002	5.23E-76
IGSF10	6.26334102	5.21E-45
AP5S1	6.245104704	2.29E-56
PITRM1-AS1	6.233992282	4.76E-57
RP1-191J18.66	6.2108736	3.54E-84
ZBTB37	6.078372138	2.77E-214
CCDC180	5.98722425	1.64E-70
HEMK1	5.915961822	5.78E-31
ZNF487	5.830067205	1.71E-69
CNTNAP2	5.730531532	9.32E-96
ZNF608	5.688392097	6.90E-91
HIST1H2BH	5.660142204	6.90E-91
TPD52L1	5.64396939	1.19E-38
SLC2A10	5.6259111	1.63E-28
CCDC146	5.606260685	2.14E-79
UBE2Q2	5.595601951	3.63E-81
TET2	5.558511814	5.11E-68
LYST	5.537886454	2.66E-23
C5AR1	5.42567695	4.09E-27
CCDC168	5.380133305	8.72E-80
ARHGEF17	5.328344868	1.50E-29
CYP27B1	5.315494032	2.55E-16
CDON	5.24243057	7.23E-13
MYOM1	5.205320067	3.27E-13
SYTL2	5.145569031	8.24E-20
GLCCI1	5.145569031	3.16E-22
ZNF214	5.145569031	5.67E-32
USP47	5.138354243	3.27E-13
FBXO22	5.130874278	6.20E-31
PPIP5K2	5.082255573	1.69E-35
ZPR1	5.050891402	4.90E-24
RBM45	5.0469225	1.69E-35
GREB1L	4.989146169	4.27E-33
PRRT2	4.982070299	3.22E-37
ZNF280D	4.952923953	1.54E-60
DNAJC18	4.949833245	2.55E-22
GABBR1	4.92317661	1.69E-35
UNC119B	4.92317661	4.90E-24
RNF216	4.882534625	1.54E-60
TTLL6	4.87792869	8.64E-20
CNGA1	4.875832663	1.87E-41
BHMT	4.874267009	1.48E-13
ADSL	4.849176028	4.71E-18
ONECUT1	4.830067205	4.63E-39

MPRIP	4.793158818	3.20E-13
RPL37	4.767898384	4.12E-41
ZBED8	4.747605045	6.69E-19
KYNU	4.730531532	6.32E-26
CACNA1D	4.730531532	5.98E-21
RP11-1072C15.6	4.716773898	2.06E-47
ZNF417	4.700784188	1.82E-10
PPM1L	4.678064112	1.13E-29
SPTBN4	4.678064112	5.66E-16
AC091729.9	4.660142204	1.71E-11
ZNF618	4.660142204	1.54E-17
EBF3	4.623616328	3.46E-12
C1orf116	4.60500065	7.23E-13
RECK	4.602634367	6.69E-19
SEMA3A	4.561296941	1.57E-35
HIST1H4H	4.56060653	5.98E-21
PLCH1	4.56060653	1.60E-12
CARD8	4.555444825	6.03E-27
IL32	4.534611322	3.50E-16
TGFA	4.50813911	8.18E-56
RP11-282K24.3	4.496066278	9.50E-08
ZNF514	4.42567695	1.57E-35
AC009133.12	4.420634372	3.94E-45
HSPA12A	4.382608228	9.36E-49
IFT80	4.375986776	4.20E-09
GNPTAB	4.363749201	9.09E-09
ZNF559-ZNF177	4.338214109	1.65E-41
LPAR2	4.338214109	3.56E-33
TET1	4.338214109	1.54E-17
ATP10D	4.338214109	7.11E-23
AP1S3	4.315494032	1.51E-29
POLD4	4.315494032	1.99E-08
TMOD2	4.301688233	3.36E-17
ANKRD31	4.289731043	3.73E-39
CACNG8	4.274852048	1.68E-18
SOX2-OT	4.266928328	3.64E-23
SLC2A12	4.245104704	7.95E-31
KIAA0319	4.245104704	8.00E-13
NAMPT	4.245104704	3.46E-21
CTD-3088G3.8	4.245104704	7.95E-31
IDS	4.245104704	7.95E-31
INPP4B	4.234207807	1.71E-11
LRCH1	4.223571543	1.63E-26

NLGN4Y	4.21286765	7.75E-25
COX20	4.212683227	1.77E-11
METTL22	4.183341698	3.62E-15
PLEKHG1	4.183341698	1.74E-28
C15orf39	4.145569031	2.09E-06
DIS3L2	4.145569031	1.68E-16
TENM1	4.145569031	1.74E-28
MAST4	4.145569031	4.20E-09
LINC00894	4.119573822	8.24E-12
SIGLEC10	4.082375204	7.77E-19
CDADC1	4.082255573	3.91E-32
MBNL1	4.075179703	3.74E-27
TRAF1	4.075179703	2.05E-07
GEN1	4.075179703	3.91E-32
KLHL3	4.075179703	1.99E-39
RP1L1	4.074718152	1.77E-12
TNPO2	4.063744808	2.10E-06
SEMA3A	4.044535907	1.68E-16
ZNF804A	4.040948599	3.91E-32
BEGAIN	4.038653827	3.71E-26
AK3	4.038653827	4.50E-06
NKX3-2	4.038653827	4.50E-06
ARNTL	4.010639451	7.83E-18
EGR3	3.993565937	8.66E-10
ZCCHC2	3.982070299	8.81E-36
FAM204A	3.951881396	3.57E-15
WEE2-AS1	3.92317661	7.69E-24
SLF1	3.92317661	3.71E-31
CIB1	3.92317661	1.99E-08
PCMTD2	3.92317661	8.19E-13
WDFY2	3.92317661	2.07E-05
CASC5	3.92317661	1.68E-16
ALG12	3.92317661	1.84E-38
TMOD2	3.92317661	7.69E-24
DNHD1	3.92317661	1.99E-08
HOXC11	3.898888309	7.69E-24
MAPKBP1	3.890755132	6.98E-21
FAM193B	3.882534625	3.57E-20
CABLES2	3.882534625	1.59E-13
OLFM2	3.87070919	8.20E-11
ZKSCAN2	3.861776065	2.07E-05
CHPF2	3.849176028	7.77E-16
MNX1	3.849176028	9.10E-09
MFSD4B	3.849176028	7.77E-16

DNAJC16	3.840714449	9.43E-08
PTCD3	3.836404179	7.45E-14
C9orf91	3.819221167	1.84E-38
NFAT5	3.797645727	4.44E-05
SNRPD1	3.797645727	1.54E-21
UPB1	3.797645727	1.54E-21
MITF	3.789425221	1.88E-26
TADA2A	3.781501501	6.98E-21
LOXL4	3.775619421	3.28E-17
SYNE1	3.775619421	1.48E-16
NUDCD2	3.773357427	1.54E-21
PIGZ	3.764478864	3.28E-21
APOB	3.759236318	3.34E-25
ACACB	3.756049047	1.67E-15
VGLL3	3.753251608	3.47E-26
TOR1AIP1	3.745220586	4.45E-07
LLPH	3.745220586	1.51E-24
ANXA2R	3.730531532	1.48E-20
TMEM27	3.730531532	1.88E-26
MBD5	3.730531532	1.48E-20
MICALCL	3.719020504	7.18E-13
PIK3CD	3.700784188	8.08E-34
CCNB3	3.692035487	7.78E-09
SMPD5	3.688711356	1.93E-18
CBR3-AS1	3.678064112	1.60E-25
C16orf82	3.678064112	8.08E-34
RTKN2	3.674867124	5.39E-11
ARFGEF3	3.667218074	9.39E-23
ITGA11	3.660142204	2.57E-15
ZFHX4	3.660142204	3.85E-30
IPMK	3.660142204	1.76E-10
N6AMT1	3.648069371	1.58E-21
NYAP1	3.601248515	2.37E-29
ARIH2OS	3.600134894	3.86E-14
FAM110B	3.567032799	9.67E-06
USB1	3.56060653	2.07E-05
MFAP3L	3.56060653	7.82E-18
ZNF774	3.553391743	1.58E-21
PARD3B	3.55315858	7.76E-22
MAP1B	3.544664986	1.06E-10
UCN	3.544118407	4.18E-10
CTD-2165H16.4	3.543949186	3.66E-09
INHBA	3.541687192	1.37E-20
STK10	3.530859187	2.05E-07

CLEC2D	3.510323021	3.98E-14
RP13-415G19.2	3.50813911	6.84E-12
ZBED8	3.50813911	4.83E-22
NAV2	3.50813911	4.23E-04
TGM1	3.50813911	1.54E-07
ELMOD2	3.498678781	7.33E-08
ZCWPW1	3.496643471	5.90E-07
FBXO24	3.467497126	1.16E-06
CMTR2	3.444008773	4.44E-05
GLDN	3.43043456	7.78E-09
CCDC127	3.429529276	1.99E-14
MTSS1L	3.42567695	1.15E-06
FOXO1	3.42567695	5.69E-16
CLEC2D	3.421982466	5.39E-11
NUP35	3.421982466	9.65E-07
EIF3M	3.408603437	4.23E-04
PRRT2	3.408603437	2.81E-19
HTT	3.407887637	2.11E-14
RP11-352D13.6	3.39328677	9.47E-05
CGNL1	3.382608228	1.15E-06
HIVEP1	3.380724695	2.21E-18
MLH3	3.377742473	4.58E-22
MED7	3.373838019	2.41E-15
MURC	3.368910906	2.34E-11
STC2	3.364870744	2.21E-18
SPINK5	3.363239453	3.50E-08
AC019181.2	3.338214109	2.10E-10
ENKUR	3.338214109	8.89E-04
ATG10	3.338214109	2.10E-10
WDR12	3.338214109	3.67E-21
CTBP1-AS2	3.338214109	5.47E-19
BTBD8	3.338214109	5.61E-12
CTSL	3.338214109	8.89E-04
CPHL1P	3.3122189	1.15E-11
SEC23IP	3.30784046	2.07E-05
ZSCAN16-AS1	3.301688233	1.19E-22
URB2	3.300146068	8.89E-04
ALDH4A1	3.297572124	1.65E-17
KCNQ1OT1	3.289051139	4.07E-17
AKAP13	3.283072555	1.32E-15
NABP1	3.281285552	1.35E-12
ZNF714	3.253325211	1.32E-15
NTSR1	3.252484235	1.76E-05
HSF2	3.245104704	2.00E-04

TMEM86B	3.241252379	6.09E-12
CDKN2A	3.238678435	2.00E-10
GNA14	3.236583719	3.67E-21
SLC19A3	3.220230036	8.89E-04
C14orf37	3.212683227	1.84E-13
TMEM199	3.200710585	2.00E-04
CFAP45	3.19065692	1.65E-17
RP11-34F20.7	3.186211015	1.54E-07
UMAD1	3.186211015	3.11E-20
ZNF470	3.182094907	2.00E-04
ZNF565	3.174715377	2.00E-10
EIF4G3	3.174715377	2.00E-04
SYNE1	3.168628047	4.23E-21
DNAJB5	3.168289107	8.31E-10
FIZ1	3.157641863	2.08E-05
MOB1B	3.145569031	1.59E-12
EFR3B	3.145569031	8.89E-04
TDRD3	3.145569031	1.59E-12
DYNC2H1	3.129627487	6.76E-09
DLG1-AS1	3.122412699	2.00E-10
TMEM106A	3.122080678	4.34E-15
ZNF554	3.119573822	4.44E-05
PNKP	3.107601181	1.31E-16
ZNF112	3.107601181	1.31E-16
PKDREJ	3.103748855	4.31E-19
EFCAB5	3.103748855	6.79E-03
HDAC5	3.100714795	2.00E-04
POU2F1	3.093101611	5.67E-13
PCDHGA5	3.093101611	3.78E-13
TECPR2	3.08370917	5.67E-13
N4BP2	3.075179703	7.30E-14
ZNF2	3.071568449	2.00E-04
MXRA8	3.067566519	4.57E-07
JAK2	3.055814378	3.50E-03
GAS6-AS1	3.054058681	1.41E-08
CACNG8	3.052459626	5.67E-13
ATP6V0A1	3.048707492	6.09E-12
NEK11	3.046033357	4.57E-07
TIGD4	3.042942649	1.25E-17
CTD-2366F13.1	3.040948599	1.31E-16
PCNX1	3.038653827	6.79E-03
TIGD6	3.038653827	1.31E-16
ZNF546	3.038653827	6.79E-03
B3GALT6	3.030785584	2.24E-12

NABP1	3.023089703	2.00E-10
PAX8-AS1	3.016286014	8.89E-04
FAM65C	3.016286014	2.27E-07
COL12A1	3.016286014	8.89E-04
DSEL	3.013779158	2.22E-19
IER2	3.010639451	8.89E-04
SYTL2	3.003029917	3.44E-09
ZNF26	2.990290805	8.33E-11
ZNF354B	2.982070299	8.33E-11
ZNF525	2.982070299	8.89E-06
KIAA1147	2.982070299	2.00E-04
SAMD12	2.982070299	3.54E-14
NEBL	2.975644029	1.37E-04
PCNX4	2.975644029	1.75E-15
ANKRD33B	2.975644029	5.27E-13
SH2D3A	2.967570729	4.23E-04
ENTPD3-AS1	2.965687196	5.67E-13
ZNF561	2.957781998	1.31E-16
IRS2	2.949999378	9.47E-05
MPRIP	2.936987476	8.89E-04
STOX2	2.935149251	2.34E-09
CCDC122	2.931444226	1.41E-08
ZNF564	2.928177291	8.89E-04
CTA-390C10.10	2.92317661	5.27E-13
NAP1L1	2.92317661	1.32E-02
AC009303.2	2.92317661	6.79E-03
NIPAL2	2.92317661	6.79E-03
RP5-1148A21.3	2.92317661	8.19E-10
OXTR	2.92317661	1.37E-04
FAM117A	2.92317661	1.32E-02
FSTL4	2.92317661	5.27E-13
BCL11B	2.92317661	5.27E-13
ZNF205	2.92317661	1.32E-02
PLEKHG3	2.92317661	4.76E-16
ST3GAL3	2.92317661	1.76E-14
TBX15	2.92317661	2.71E-04
IL31RA	2.92317661	1.32E-02
CLEC16A	2.90867704	2.55E-02
MYO10	2.902418049	3.39E-15
KCNQ5	2.901150303	1.41E-08
DNAJC21	2.900456533	1.77E-11
GTPBP4	2.89855274	3.54E-14
TSC22D2	2.895787111	1.37E-04
AKAP6	2.895787111	4.24E-04

PDE4B	2.895787111	1.37E-04
FAM218A	2.890755132	2.48E-14
SATB1	2.888411191	7.13E-14
RNF183	2.885108569	8.89E-04
OPHN1	2.882534625	1.75E-15
PLEKHH1	2.87878249	2.55E-02
P2RY6	2.87878249	6.14E-10
PTPN21	2.864995538	5.05E-08
ITPR2	2.861776065	6.14E-10
FAM110A	2.861776065	1.37E-04
ZNF528	2.861776065	1.37E-04
CARM1	2.859160117	2.24E-12
FLJ31356	2.856539416	6.56E-15
ZNF827	2.852240703	1.49E-15
KDM6A	2.840714449	1.20E-09
PLPP6	2.840714449	1.77E-11
NOTCH4	2.823640936	5.27E-13
FLJ16779	2.823640936	6.91E-05
GZF1	2.823640936	1.20E-09
YY2	2.823640936	1.76E-05
NXPH4	2.820365804	6.74E-12
CALCOCO1	2.817381946	4.59E-09
CCDC33	2.812145297	1.84E-13
ABCA1	2.812145297	9.91E-13
SPPL2A	2.81070188	6.74E-12
BCHE	2.808324269	3.50E-05
PCDHGB5	2.800319862	4.57E-07
MURC	2.797645727	2.71E-04
ICA1L	2.797645727	2.55E-02
TRIOBP	2.797645727	1.26E-06
SLC9A6	2.797645727	2.71E-04
CTD-2184D3.5	2.797645727	2.48E-14
PPM1L	2.797645727	1.32E-02
CTB-176F20.3	2.797645727	2.55E-02
ZNF136	2.794728244	1.87E-12
AKAP6	2.791024275	4.59E-09
LGR4	2.785673086	1.29E-11
MCM8	2.782998951	6.79E-03
AK5	2.775619421	4.83E-14
FAM222A-AS1	2.770325121	9.56E-08
MYLIP	2.767898384	9.04E-11
RP13-516M14.2	2.761895702	3.50E-03
FAHD2A	2.754498399	1.87E-12
PLLP	2.753251608	7.34E-06

SGSM1	2.753251608	1.26E-06
KLHL28	2.753251608	8.89E-04
SCG2	2.753251608	4.59E-09
PCNX4	2.753251608	1.38E-12
STARD9	2.738144716	3.43E-08
AGO4	2.732493048	6.98E-11
PRRT2	2.730531532	6.79E-03
NLRC5	2.730531532	5.86E-15
ABHD18	2.730531532	6.79E-03
ALPK1	2.730531532	2.55E-02
ZNF469	2.730531532	8.98E-09
GIGYF2	2.725372576	3.46E-07
AC005154.6	2.723867801	1.85E-06
RP11-1299A16.3	2.707447918	3.50E-03
NEIL1	2.700784188	9.13E-07
TCAP	2.700784188	8.89E-04
FAM46A	2.700237609	2.46E-11
STARD9	2.697616909	6.79E-03
GBP1	2.688711356	3.70E-06
ZNF430	2.678064112	4.23E-04
ZBED3-AS1	2.671637843	9.39E-13
ZMAT1	2.660142204	4.92E-02
RNASEL	2.660142204	4.92E-02
KATNAL1	2.660142204	1.93E-09
LONRF2	2.660142204	4.92E-02
CMTR2	2.660142204	1.37E-04
SEMA4F	2.660142204	6.79E-03
F8	2.660142204	4.92E-02
MMP28	2.660142204	4.92E-02
ZNF326	2.660142204	1.03E-11
ZNF382	2.660142204	4.92E-02
TET2	2.656390069	1.82E-07
ZNF529	2.655325187	1.32E-02
KB-176G8.1	2.64638457	2.01E-11
NABP1	2.629953301	2.54E-07
GPRIN3	2.627720726	6.38E-12
GRAMD1C	2.627720726	6.38E-12
CLCN4	2.627438039	3.50E-03
PPM1L	2.618322028	9.04E-11
FAT4	2.615679191	1.05E-03
UBAP1L	2.615679191	2.91E-05
F8	2.615054314	7.38E-06
IGFBP5	2.615054314	6.79E-03
FXYD3	2.601248515	8.90E-04

SOGA3	2.601248515	1.37E-04
NIM1K	2.601248515	1.37E-04
FAM222A	2.601248515	1.32E-02
GSDMB	2.601248515	6.70E-08
TCEANC2	2.601248515	6.41E-10
SERPINI1	2.598336919	1.92E-04
CEP97	2.594452833	1.25E-08
NAIF1	2.591970701	1.26E-06
TTC41P	2.589752876	6.38E-12
CTB-171A8.1	2.586141622	2.55E-02
FAHD2A	2.586141622	6.41E-10
SMOC1	2.575253306	1.25E-08
9-Mar	2.57441233	1.25E-08
NFAT5	2.572679362	1.25E-06
PCDHGB3	2.567032799	1.23E-09
BCL2L14	2.567032799	1.32E-02
ADGRL3	2.564722639	1.23E-09
DOPEY1	2.56060653	1.31E-07
SSX2IP	2.56060653	4.54E-09
RSPH4A	2.56060653	2.40E-10
CLDN4	2.56060653	2.40E-10
ZNF514	2.56060653	8.90E-04
WDR3	2.56060653	1.74E-10
CNDP2	2.555444825	3.50E-03
LRCH1	2.553227	3.43E-08
UBN1	2.54418186	3.43E-08
L3MBTL3	2.542086442	2.36E-09
ADAM32	2.53984797	2.55E-02
BNIPL	2.527247933	2.30E-11
TARSL2	2.527247933	2.54E-07
APBB1	2.519634749	2.55E-02
KLHL23	2.512168476	8.20E-08
TMEM81	2.50813911	1.32E-02
ARAF	2.50813911	2.33E-08
WDR12	2.50813911	4.92E-02
MSS51	2.50813911	4.92E-02
TROVE2	2.50813911	6.79E-03
KLHL38	2.50813911	4.92E-02
RBM20	2.50813911	4.92E-02
PDE11A	2.50813911	4.92E-02
RP11-341G23.3	2.50813911	4.92E-02
C12orf76	2.504023002	2.41E-06
ARMCX4	2.497293072	8.90E-04
MUC15	2.490509216	6.41E-10

PCDHGA5	2.485771297	7.10E-11
APBB1IP	2.479569958	1.32E-02
SLCO2B1	2.477617165	1.32E-02
UBR3	2.475717633	4.92E-02
RP11-209D14.2	2.463744991	4.92E-02
ZC3H6	2.460767196	4.37E-08
RP11-521B24.3	2.457513037	4.92E-02
MAGEE1	2.457513037	6.79E-03
HIST1H3E	2.453691326	6.79E-03
ETFBKMT	2.453691326	1.92E-04
ZBTB32	2.453691326	8.69E-09
TMX4	2.453691326	8.69E-09
ANTXR1	2.448638099	1.05E-03
DOCK4	2.445819723	4.37E-08
AMER1	2.445129313	6.79E-03
BCORL1	2.444413513	2.40E-10
MAP1A	2.437749782	8.17E-10
BTBD9	2.437749782	1.54E-07
RP11-129M16.4	2.437749782	4.54E-09
CFLAR	2.437089359	5.20E-09
TLDC2	2.434138529	2.30E-11
USP42	2.4220457	2.40E-10
TRIM13	2.415723476	2.99E-10
RP11-359B12.2	2.403169551	3.17E-08
EPCAM	2.401344791	1.01E-06
ZNF627	2.401223906	6.79E-03
FARP1	2.397107798	1.54E-07
RP11-412D9.4	2.394797637	9.51E-07
TMEM232	2.39328677	1.67E-05
CREG2	2.39328677	3.97E-03
CCDC113	2.388840182	1.94E-07
ALOX5	2.382608228	2.71E-04
PRKAG2	2.381282831	1.66E-08
MRAS	2.376349238	1.66E-08
NMNAT1	2.373547182	1.66E-08
TPK1	2.370635586	7.61E-03
RP11-412D9.4	2.36957828	4.92E-02
OAS2	2.369386277	6.00E-05
PLCE1	2.367961452	3.17E-08
ATP1A1	2.366422936	1.21E-03
SRCIN1	2.365694845	6.79E-03
PTPN21	2.362177352	3.17E-08
HFE	2.355287622	1.66E-08
ACTRT3	2.338214109	1.04E-04

PON2	2.338214109	1.04E-04
TGFB3	2.338214109	1.66E-08
HIVEP2	2.338214109	5.98E-06
DNHD1	2.338214109	5.98E-06
KIAA1211L	2.338214109	6.54E-04
ZNF292	2.338214109	9.51E-07
NUDCD3	2.338214109	1.79E-08
AC009473.1	2.338214109	6.54E-04
STC2	2.338214109	1.79E-08
RP11-77P6.2	2.338214109	6.03E-08
PLEKHG1	2.338214109	1.05E-03
INTS9	2.338214109	6.79E-03
ARHGEF9	2.333160881	3.32E-08
ALX1	2.330086228	1.32E-02
SEMA4C	2.326141277	1.54E-07
FBXO25	2.318039397	5.98E-06
PRKD2	2.315130496	2.55E-02
SNX24	2.311741897	4.92E-02
CDK18	2.310335076	1.89E-06
ALDH1L2	2.307889572	6.03E-08
NDUFS1	2.30784046	5.20E-09
NRDE2	2.303998393	5.20E-09
IPP	2.301688233	6.46E-09
FMN1	2.301688233	1.79E-08
SYT1	2.301688233	4.92E-02
PCDHGA5	2.297572124	6.29E-07
KIAA1958	2.297572124	5.40E-07
DAGLA	2.292410419	1.79E-08
ZNF580	2.290171252	8.77E-06
CTC-351M12.1	2.281368248	2.07E-08
PROSER3	2.279797423	4.92E-02
AC091729.9	2.279661926	6.46E-09
ZBTB37	2.27932042	1.16E-08
TTC25	2.279052036	1.32E-02
PHLPP1	2.277331867	1.94E-07
ZNF101	2.276813564	6.46E-09
ZNF316	2.276365453	1.94E-07
RP11-379H18.1	2.27557339	1.89E-06
CLUH	2.274900651	1.01E-06
QARS	2.269314921	5.40E-07
ZNF493	2.267755888	1.01E-06
PLEKHA5	2.262663076	1.67E-05
TMEM33	2.261592827	4.92E-02
PHLPP1	2.257666779	1.09E-05

AGPAT4	2.257294113	2.88E-07
ZNF490	2.255751949	1.07E-09
SPAG16	2.253325211	6.79E-03
NUP54	2.249804662	3.56E-04
ZDHHC3	2.247240449	2.55E-02
FARP1	2.245104704	1.79E-08
ITPR2	2.245104704	4.92E-02
FANK1	2.245104704	7.61E-03
PDZD8	2.245104704	1.32E-02
NDUFA10	2.238678435	4.92E-02
ZBTB3	2.233609066	3.57E-05
PLXNC1	2.233352564	3.57E-05
TIMM22	2.233031872	3.96E-03
SSH1	2.227790119	3.17E-05
RP11-649A18.12	2.225831556	1.97E-05
FAM162A	2.22573938	5.40E-07
FAM213A	2.224573638	6.79E-03
WDR13	2.218632493	6.29E-07
CCDC151	2.214225392	6.16E-08
NXPE3	2.212683227	1.20E-03
SLC34A3	2.212683227	6.29E-07
FAM227A	2.212683227	1.21E-05
VPS13D	2.212683227	2.55E-02
TXNDC12	2.211124193	1.97E-05
LRRC20	2.208578828	4.92E-02
KLHDC2	2.203787272	1.32E-02
CCDC157	2.203284529	1.13E-06
ZNF470	2.203284529	3.96E-03
STXBP4	2.19773279	6.46E-09
TMEM57	2.186211015	2.05E-03
RPAP2	2.186211015	2.55E-02
TMEM63C	2.186211015	2.10E-07
TRAF3IP1	2.182935883	4.92E-02
IPO9	2.168483423	3.88E-07
SPPL2B	2.168289107	6.56E-04
AC002310.7	2.162642544	6.47E-05
ZNF107	2.162642544	3.51E-06
ZNF469	2.157641863	2.04E-06
AAK1	2.153789538	1.89E-06
CSK	2.153474229	4.04E-03
ARHGAP22	2.150427076	4.92E-02
DGAT1	2.145569031	3.57E-05
RHOH	2.145569031	1.45E-02
POP4	2.145569031	4.92E-02

GHRL	2.142512472	1.14E-07
GIT1	2.140768045	6.29E-07
DNASE1	2.138682645	7.12E-07
RP11-299G20.5	2.136170333	4.92E-02
CPLX2	2.134073392	1.14E-07
ARHGEF26	2.131763231	2.10E-07
ACACA	2.126203706	1.32E-02
AC109829.1	2.123661352	3.57E-05
NIFK-AS1	2.119573822	2.55E-02
ELP2	2.118248425	3.67E-06
ZFYVE16	2.115821687	6.66E-08
ZNF469	2.115821687	6.66E-08
TACC2	2.115821687	2.10E-07
ZNF525	2.105768023	2.05E-03
C22orf39	2.103748855	3.96E-03
ARL5B	2.103748855	3.96E-03
ANKH	2.099438585	7.12E-07
OTUD6B-AS1	2.098263316	1.32E-02
TCEANC	2.093101611	3.93E-04
TMEM184A	2.076781808	1.21E-05
CLASP1	2.075179703	4.03E-03
TIAM2	2.075179703	4.92E-02
PCLO	2.075179703	4.92E-02
TATDN2	2.075179703	1.32E-02
AC062029.1	2.075179703	3.67E-06
FAM84B	2.074249832	1.45E-02
PRH1	2.070834233	4.92E-02
DUS2	2.061392751	2.14E-07
OTX1	2.060680133	4.92E-02
GSTCD	2.060680133	4.92E-02
ZNF525	2.05810619	1.17E-04
RAB11FIP5	2.057752631	4.92E-02
TTC3	2.056150475	4.07E-05
SGMS1-AS1	2.054058681	1.21E-05
EVX1	2.052459626	2.21E-03
PLA2R1	2.050891402	1.21E-05
SLC13A4	2.049184494	1.18E-05
ZNF714	2.04543236	4.92E-02
RP11-75C10.6	2.044224504	6.59E-06
RP13-467H17.1	2.038653827	1.45E-02
ZNF678	2.038653827	3.94E-04
FKBP15	2.034537718	4.07E-05
SLF1	2.027873988	1.45E-02
BCAS1	2.027873988	4.92E-02

TUBB3	2.027513269	3.88E-07
RP3-424M6.4	2.026270102	2.71E-02
ZEB1-AS1	2.022240737	1.20E-07
AC007128.1	2.016286014	4.04E-06
ACRBP	2.013251954	3.95E-04
RILPL1	2.010027023	2.71E-02
THUMPD1	2.008065507	4.92E-02
OPA3	2.00563877	4.92E-02
SPARCL1	2.001179122	4.03E-03
JPH1	2.001179122	4.03E-03
DENND2C	1.995091792	7.61E-03
DZIP1L	1.994632374	2.22E-05
FLRT1	1.993565937	4.92E-02
ADAMTS7	1.993565937	1.45E-02
DNHD1	1.990290805	2.11E-04
PITRM1-AS1	1.984690999	3.95E-04
ARID1B	1.984105608	7.61E-03
ZNF580	1.983297602	2.21E-03
WDCP	1.982070299	4.92E-02
GATA4	1.982070299	4.92E-02
SEMA3D	1.978249271	4.04E-03
ZNF446	1.978217973	6.59E-06
L3MBTL4	1.975644029	7.01E-06
AC004893.11	1.973549314	1.30E-06
USP2	1.971173401	2.71E-02
AMMECR1	1.970482324	4.92E-02
TRIM52	1.970482324	4.99E-02
AC074117.10	1.968691269	4.04E-06
KLHDC4	1.968264499	4.92E-02
ZFP69B	1.964996785	2.55E-02
GCNT4	1.962704974	4.92E-02
RP11-734K2.4	1.962704974	4.07E-05
GNG2	1.962704974	1.30E-06
EFCAB7	1.959702486	2.22E-05
SLC27A1	1.949648821	3.84E-07
SLFN5	1.947143477	1.23E-06
BAHCC1	1.94499472	4.07E-05
DTNA	1.94393517	4.07E-05
ITGB1BP2	1.932145073	2.19E-06
SLAIN2	1.926928744	4.04E-06
KMT2D	1.925195777	3.76E-05
SPATA33	1.92317661	4.92E-02
AQP6	1.92317661	4.92E-02
TIAM2	1.92317661	2.71E-02

LINC00884	1.92317661	3.78E-04
RP3-323A16.1	1.92317661	7.61E-03
RP1-228H13.5	1.92317661	1.21E-05
RP11-16N11.2	1.92317661	1.45E-02
LBHD1	1.92317661	2.17E-04
RP11-318A15.2	1.92317661	3.95E-04
NGRN	1.92317661	1.34E-03
FAM126B	1.92317661	1.26E-04
PARP9	1.913192521	4.04E-06
KIAA1024	1.912330572	2.55E-02
COL8A1	1.900808796	4.92E-02
SLC43A2	1.894760927	3.89E-06
PIK3CD	1.886469388	2.19E-06
CTC-510F12.7	1.885593427	7.31E-04
PCDHGB5	1.885208759	1.26E-04
FBP1	1.884496141	3.75E-04
DSCR3	1.884496141	2.55E-02
SP140	1.882534625	2.71E-02
CELF3	1.882534625	1.26E-04
RP11-182J1.18	1.882534625	2.55E-02
TAS2R4	1.868964342	3.63E-05
SRGAP1	1.868728826	4.92E-02
SERINC2	1.868035055	6.75E-04
UBE4A	1.858246324	7.01E-06
PATZ1	1.858008424	1.18E-04
LMCD1	1.852787282	6.89E-06
ADAP1	1.852787282	1.21E-05
AKTIP	1.849559476	3.89E-06
ZNF81	1.849176028	4.92E-02
RHOQ	1.847227756	7.30E-03
DLEU2	1.846008749	5.00E-02
HTRA1	1.840714449	3.76E-05
LINC01137	1.840714449	4.99E-02
WDR13	1.839224831	1.21E-05
ZNF519	1.834431936	6.89E-06
ZIC5	1.832978801	7.05E-03
ERLIN2	1.832173411	2.10E-05
DNAJC21	1.830067205	3.89E-06
MED26	1.830067205	3.78E-04
NFATC2IP	1.824867196	2.07E-04
PDE4C	1.823640936	4.92E-02
ZNF615	1.823640936	4.92E-02
PCDHA2	1.823640936	1.45E-02
EPHB2	1.823222824	6.26E-05

NIN	1.821062392	4.92E-02
USP30	1.821062392	4.92E-02
MXRA7	1.820606876	4.10E-03
FNDC3A	1.819221167	3.89E-06
TMPO-AS1	1.817654641	6.89E-06
ACVR1B	1.814652153	3.76E-05
KCNK6	1.812145297	4.92E-02
PIAS2	1.808324269	4.10E-03
RP3-406P24.3	1.808324269	1.20E-03
BRCA2	1.808324269	4.04E-03
NLK	1.808240843	2.10E-05
PTPRG	1.805866234	2.07E-04
RP4-605O3.4	1.804781909	6.45E-04
CSNK1A1	1.797645727	1.31E-02
MYD88	1.79399795	6.75E-04
ATP11B	1.791932076	1.18E-04
PRKD2	1.78545043	7.31E-03
CST3	1.781157605	5.00E-02
RASSF2	1.7787867	4.03E-03
SSSCA1-AS1	1.7772853	4.92E-02
SNX1	1.776805613	6.68E-05
FAT4	1.776117188	6.89E-06
ZNF529	1.774313224	1.21E-05
UBE2D4	1.771173516	4.92E-02
CNTRL	1.771173516	5.00E-02
CLIP3	1.771173516	1.18E-04
HOTAIR	1.76866666	4.92E-02
RHBDL2	1.757703611	4.92E-02
PDE11A	1.753251608	5.00E-02
RPGRIP1L	1.753251608	5.00E-02
PNPLA7	1.753251608	1.20E-03
DDN	1.753251608	3.63E-05
RP11-464F9.20	1.753251608	1.08E-04
TACC1	1.753251608	5.00E-02
ZBTB7B	1.747151409	2.07E-04
BAHD1	1.746298847	2.07E-04
ZNF772	1.745638424	3.63E-05
SRSF8	1.742604364	3.75E-04
SNHG14	1.741338287	2.07E-04
ADGRF4	1.739995511	4.92E-02
SAMD8	1.738752038	2.71E-02
ZNF417	1.733205666	1.84E-04
DSG2	1.732931624	3.63E-05
FLJ31356	1.730531532	7.05E-03

TYMSOS	1.730531532	1.45E-02
NHLRC3	1.728881794	2.13E-05
PLEKHA5	1.728160627	1.08E-04
PAN2	1.7272564	1.18E-04
DENND4A	1.723504265	4.92E-02
ATAD1	1.721379833	3.62E-04
ATXN7	1.721071202	4.92E-02
SLITRK6	1.716725732	5.00E-02
SP4	1.715214865	1.31E-02
LCLAT1	1.710265683	2.13E-05
P2RY1	1.709772971	4.92E-02
MYO1E	1.707447918	5.00E-02
CGNL1	1.704996439	1.45E-02
ZNF655	1.703409658	1.18E-04
VTA1	1.700784188	2.71E-02
TRIM56	1.693006838	5.00E-02
ZFHX4	1.691851063	2.33E-02
TNFAIP3	1.686137412	5.00E-02
POM121	1.686137412	3.68E-03
GVQW2	1.683710675	5.00E-02
RP11-573D15.8	1.683173172	4.99E-02
CPSF7	1.680609477	3.62E-04
HSD17B2	1.678978846	1.08E-04
ARID1B	1.678064112	4.92E-02
SIX4	1.677442198	7.06E-03
BTBD9	1.672704279	1.31E-02
ZNF292	1.670789448	1.88E-03
HELLPAR	1.667218074	1.10E-03
MAP2K7	1.665378852	1.02E-04
SHISA2	1.660142204	4.92E-02
MNT	1.660142204	4.08E-02
TET1	1.65252902	5.00E-02
PRUNE2	1.648554229	1.70E-04
ELFN2	1.646336404	2.71E-02
RSU1	1.64306869	2.71E-02
EMC10	1.635518417	2.07E-04
FAM104B	1.635374299	6.28E-04
TAT	1.635374299	3.68E-03
KMT5C	1.633669992	2.83E-04
MTCL1	1.630995858	5.00E-02
PDE11A	1.63039486	4.99E-02
RP5-1157M23.2	1.627720726	1.74E-03
FMN1	1.627720726	3.62E-04
NME9	1.62061384	3.68E-03

PRICKLE1	1.618322028	1.31E-02
NAP1L6	1.617073482	1.31E-02
PPARD	1.61701583	5.00E-02
CASC2	1.615679191	1.10E-03
DCLK2	1.611232603	1.21E-02
PLAGL2	1.610394377	3.14E-04
HIF1AN	1.607400742	3.14E-04
RNF219	1.60500065	5.00E-02
PPP1R3F	1.601248515	2.71E-02
ARID3A	1.601248515	6.28E-04
TNIK	1.601248515	2.83E-04
AKAP7	1.601248515	2.71E-02
PNPLA6	1.596788866	2.11E-03
ATP5E	1.589059106	1.88E-03
GFOD2	1.586354299	1.84E-04
SETD7	1.579222208	3.14E-04
GATA6	1.576521429	3.62E-04
PCDHGA10	1.573644053	1.84E-04
PUM2	1.571647318	2.83E-04
MOCS3	1.571020139	3.43E-02
ZNF16	1.562869582	1.70E-04
PROC	1.56060653	4.08E-02
MLXIP	1.553126565	1.02E-04
BTN3A2	1.550988528	4.67E-04
SCARA5	1.547477258	4.67E-04
NGRN	1.53984797	3.67E-03
FAT4	1.53984797	2.71E-02
PIANP	1.538512759	7.06E-03
SLC25A45	1.534611322	4.67E-04
CDSN	1.530859187	2.83E-04
CCDC88A	1.530859187	2.83E-04
CCDC170	1.530859187	1.31E-02
RAP2A	1.528185052	5.00E-02
ATHL1	1.524080654	2.83E-04
AGBL3	1.519211268	7.68E-04
MYCBP2	1.518786354	1.70E-04
MYCBP	1.518123199	3.14E-04
BCL11B	1.517537808	4.08E-02
9-Sep	1.515197599	3.14E-04
CNTD2	1.513852761	2.71E-02
GDPD5	1.50925965	5.31E-04
RP11-467D6.1	1.50813911	5.00E-02
MAST4	1.505814057	8.95E-04
SLC25A25	1.498757874	3.14E-04

RAPGEF4	1.498085446	1.50E-03
ADCK2	1.495755386	2.84E-03
RP11-326C3.11	1.493032218	2.71E-02
SSR1	1.490217202	2.83E-04
ZNF550	1.489280083	4.67E-04
MEP1A	1.486396174	6.36E-03
MTHFR	1.484292368	2.33E-02
RBM33	1.483658665	3.68E-03
ATXN7L3	1.483330817	1.74E-03
GPR180	1.480658374	5.00E-02
EGFLAM	1.479569958	2.85E-03
KIAA1328	1.479226418	3.68E-03
TUBGCP6	1.473670893	5.31E-04
TMEM8A	1.470799748	7.68E-04
JAML	1.469458642	4.08E-02
TRIM39	1.467079693	3.19E-03
B4GALT5	1.459953052	2.83E-04
SAMD8	1.459060289	2.83E-04
SH3GLB1	1.453691326	7.68E-04
NAIF1	1.449051458	7.68E-04
EML1	1.44324958	7.68E-04
RP11-573D15.8	1.437749782	2.33E-02
MFHAS1	1.437728713	1.26E-03
MFSD6	1.432190257	7.68E-04
DNAJC10	1.431323513	5.00E-02
NACC2	1.427988474	4.67E-04
ARHGAP25	1.425523824	3.43E-02
STRBP	1.42168397	4.67E-04
NUDCD3	1.419827874	4.67E-04
PRX	1.41221469	8.95E-04
STARD9	1.408603437	5.00E-02
TFEB	1.40380245	8.95E-04
LRRC8D	1.400949864	4.67E-04
ALMS1	1.399540071	4.67E-04
PRPF38B	1.398863054	2.85E-03
TRIM71	1.39328677	1.09E-02
ATM	1.387079724	4.65E-03
TMEM120B	1.374739985	5.00E-02
ZNF235	1.374739985	5.00E-02
ZCCHC3	1.373873241	1.26E-03
WDR77	1.370653762	1.50E-03
NLGN2	1.36172623	7.54E-03
SAMD11	1.360436566	2.04E-03
CARF	1.356136017	7.68E-04

PDE12	1.355514103	1.09E-02
SUFU	1.345241376	2.48E-03
ATP9B	1.344877839	1.26E-03
TRIM39	1.341407165	7.54E-03
NOL12	1.338214109	2.96E-02
TNIK	1.338214109	1.26E-03
DMPK	1.338214109	4.08E-03
TSPAN4	1.332077583	2.05E-02
DEPDC1	1.323567333	2.04E-03
RAD52	1.315494032	7.54E-03
RPTN	1.301688233	4.08E-02
LIMD1	1.296393933	3.30E-03
ONECUT1	1.288173426	3.43E-02
MED26	1.285746689	1.21E-02
CCDC183	1.281048731	7.54E-03
ANKRD16	1.271099913	4.73E-02
KCNQ1OT1	1.267824781	4.08E-02
IPPK	1.266922815	4.08E-03
ADGRA2	1.265999041	2.04E-03
SNRNP35	1.26373444	3.30E-03
CCDC183	1.263667155	1.03E-02
SIPA1L2	1.259906581	7.54E-03
MYH7B	1.257666779	1.21E-02
NLGN2	1.257537463	5.28E-03
MAFG	1.243085537	3.30E-03
GIGYF1	1.2361422	5.28E-03
RP4-591C20.9	1.229204501	7.54E-03
ZNF205	1.228558396	7.54E-03
THRA	1.202729753	1.21E-02
ZNF324B	1.200305845	5.28E-03
EYA4	1.186211015	4.73E-02
C19orf44	1.181813835	3.30E-03
SPECC1	1.179060847	8.37E-03
POC1A	1.168289107	1.21E-02
ABCC5	1.1568447	5.28E-03
SLC25A44	1.154502156	8.37E-03
DIRAS1	1.151631763	8.37E-03
BNIP1	1.147549395	2.39E-02
TAPBP	1.123167704	5.28E-03
CFAP45	1.115821687	8.37E-03
IL27RA	1.115275109	8.37E-03
CADPS2	1.067207578	3.78E-02
PXN-AS1	1.064698867	8.37E-03
FAM118A	1.060174773	1.31E-02

CATSPER2	1.05169135	4.71E-02
RGN	1.016286014	3.83E-02
MUSK	1.016286014	3.83E-02
UBE2R2	1.001443473	3.78E-02

Supplementary Table 7. Antibodies used in this study.

Antigens	Lot, manufacturers	Application
GFP	sc-9996, santa cruz	1:1000 for WB
Calnexin	A4846, abclonal	1:1000 for WB
Alix	sc-53540, santa cruz	1:250 for WB
CD9	sc-13118, santa cruz	1:250 for WB
CD63	sc-5275, santa cruz	1:250 for WB
TSG101	sc-7964, santa cruz	1:250 for WB
AFP	ab169552, abcam	1:100 for IF
RAB27A	17817-1-AP, proteintech	1:1000 for WB
FN1	610077, BD	1:1000 for WB
E-Cadherin	610182, BD	1:1000 for WB
E-Cadherin	20874-1-AP, proteintech	1:1000 for WB
N-Cadherin	610921, BD	1:1000 for WB, 1:100 for IHC
ZEB1	21544-1-AP, proteintech	1:1000 for WB
GAPDH	60004-1-Ig, proteintech	1:50000 for WB
Ki67	#9027, cell signaling technology	1:400 for IHC
ALKBH5	16837-1-AP, proteintech	1:2000 for WB, 1:100 for RIP
ALKBH5	ab195377, abcam	1:1000 for IHC
DEPDC1	ab197246, abcam	1:1000 for WB, 1:200 for IHC
NTSR1	ab183088, abcam	1:1000 for WB, 1:50 for IHC
ITGB4	21738-1-AP, proteintech	1:1000 for WB
ITGA6	#3750, cell signaling technology	1:1000 for WB
EGFR	#4267, cell signaling technology	1:1000 for WB, 1:50 for IHC
m^6A	68055-1-Ig, proteintech	1:100 for meRIP, 1:1000 for m^6A dot blot
Normal Rabbit IgG	#2729, cell signaling technology	1:100 for RIP, meRIP
Alexa Fluor 488-conjugated anti-rabbit IgG	A-21206, Thermo Fisher	1:5000 for IF

Supplementary Table 8. Gene specific primers used for qRT-PCR and sequences of miRNA mimics, inhibitors, siRNA and shRNA.

Gene name		Oligo sequence
miR-223-3p		GCGTGTCAGTTTGTCAAATACCCCA
miR-6795-3p		ACCCCTCGTTTCTTCCCCC
miR-3912-5p		GCGCCGATGTCCATATTATGGGTTAGT
miR-6832-5p		GCGTCAGTAGAGAGGAAAAGTTAGGG
miR-98-3p		CGCGCGCGCGCTATACAACTTACTACT
miR-6880-3p		GATAGCGATGCCGCCTTCTCTCCTC
miR-4440		CATGTCGTGGGGCTTGCTGGCTT
miR-4793-3p		AGTCTGCACTGTGAGTTGGCTGGCT
miR-6757-3p		TGCGCAACACTGGCCTTGCTATCC
miR-3190-5p		TCTGGCCAGCTACGTCCCCA
U6		CTCGCTTCGGCAGCACA
pri-miR-3190	Forward:	GAATTAATACGACTCACTATAGGGAGAATA-GATAGTGGGCTGGGGTCACCTGTCTGGCC
	Reverse:	CCGGGGTCACCTCTCTGGCCGTCT
GAPDH	Forward:	GGAGCGAGATCCCTCCAAAAT
	Reverse:	GGCTGTTGTCATACTTCTCATGG
RAB27A	Forward:	GCTTTGGGAGACTCTGGTGTA
	Reverse:	TCAATGCCCACTGTTGTGATAAA
ALKBH5	Forward:	ATCCTCAGGAAGACAAGATTAG
	Reverse:	TTCTCTTCCTTGTCCATCTC
ABI2	Forward:	TGGCCGATTACTGCGAGAAC
	Reverse:	GGGTGGTGTAGGCTTTGGTT
ZNF70	Forward:	GAGGGGAATTTCAGTTTGTGCT
	Reverse:	TTCTGGAGGAAGGTTTGTCCG
DCAF7	Forward:	AAGCATTGATACGACATGCACC
	Reverse:	CCAGACACGAGATTCACTCGC
PTPRB	Forward:	ACAACACCACATACGGATGTAAC
	Reverse:	CCTAGCAGGAGGTAAAGGATCT
TMEM127	Forward:	CTGGTTGCACATCCACGGA
	Reverse:	CTGGGGATTCATGCAGAAATCT
DCTN5	Forward:	CAGTCGCCAGTCAGTGTTGT
	Reverse:	CGTCCAACTCTTACATTTGCCAG
LYRM2	Forward:	AGCAGTTCGTAAGAAGGCAAC
	Reverse:	GCCCAATCTTTCAGGTATTTGCG
NT5DC3	Forward:	AAGTCATTGAAATGTACGAGGGG
	Reverse:	TCATCGTGTTTCCATGAGAGC
ARHGAP29	Forward:	GACTTTCATCGAAAACTTCCACG
	Reverse:	AATTTGCGAAACTTGTGTGTGAG

LAMC1	Forward:<br>Reverse:	ACTGCCACTGACATCAGAGTA<br>GCTTGCGTGTCCATTACATTTAC
MED21	Forward:<br>Reverse:	TGGTGCTTACGGGTTCTCTT<br>AGCGCTGGTGAAGGAATACT
RGMA	Forward:<br>Reverse:	TGGTGCTTACGGGTTCTCTT<br>ACGGCTGTCTCGTATGGGA
SLC48A1	Forward:<br>Reverse:	CTGGAGTCTTCATGGCTGGA<br>TTGCCACTGTCTACCCTGAG
SLC25A34	Forward:<br>Reverse:	ACTTAGGCCTCAGACAGCAG<br>TCAAGCGCATAGGTCTAGGG
TNPO2	Forward:<br>Reverse:	TTTCGTCCTGACCAGACTCAA<br>TGTGCCTTCACGTTGTTCTTG
KLF2	Forward:<br>Reverse:	CTACACCAAGAGTTCGCATCTG<br>CCGTGTGCTTTCGGTAGTG
NSD1	Forward:<br>Reverse:	TCCTGAGTCAGAACATGACCTG<br>CGAGATTTAGCGCAAGGCTTTT
PRC1	Forward:<br>Reverse:	ACACTCTGTGCAGCGAGTTAC<br>TTCGCATCAATTCCACTTGGG
TOP2A	Forward:<br>Reverse:	ACCATTGCAGCCTGTAAATGA<br>GGGCGGAGCAAAATATGTTCC
ZMYND8	Forward:<br>Reverse:	CGAGACCCAGAGTAAAGCCAT<br>GATGTATTCCGCATAGTCAGGG
FAT1	Forward:<br>Reverse:	CATCCTGTCAAGATGGGTGTTT<br>TCCGAGAATGTACTCTTCAGCTT
MRC2	Forward:<br>Reverse:	TGACCGGGAAGCACTGAATC<br>GTGCCAGGCTTGGATATGTTG
NR4A3	Forward:<br>Reverse:	GGTCGTCTGCCTTCCAAAC<br>GCTCGGACAAGGGCATTCA
LOXL4	Forward:<br>Reverse:	CTGGGCACCACTAAGCTCC<br>CTCCTGGATAGCAAAGTTGTCAT
SIRT5	Forward:<br>Reverse:	GCCATAGCCGAGTGTGAGAC<br>CAACTCCACAAGAGGTACATCG
VEGFA	Forward:<br>Reverse:	AGGGCAGAATCATCACGAAGT<br>AGGGTCTCGATTGGATGGCA
VCAN	Forward:<br>Reverse:	GCAAGTGATGCGGGTCTTTAC<br>TTGCCGCCCTGTAGTGAAAC
SULF2	Forward:<br>Reverse:	CACTGGCAAGTACGTCCACAA<br>CTATTGAGGTACACGGCAAAGG
SMC4	Forward:<br>Reverse:	CGCCTCCAGCAATGACCAAT<br>CCCCAGCATAGGATTTGAAGTT
PDK1	Forward:<br>Reverse:	GGATTGCCCATATCACGTCTTT<br>TCCCGTAACCCTCTAGGGAATA
NR4A1	Forward:<br>Reverse:	GGCTCGGGGATACTGGATACA<br>CTGGCATGAAGCGTTGTCC
MYBL1	Forward:<br>Reverse:	AGGCAAGCAGTGTAGAGAAAGA<br>CGATTTCCCAACCGCTTATGT
KIF23	Forward:<br>Reverse:	TGTGGCTAATCCCTTGGTCAA<br>AGAACCAGTCATTGTGTGAGTTT
KDM6A	Forward:<br>Reverse:	TACAGGCTCAGTTGTGTAACCT<br>CTGCGGGAATTGGTAGGCTC

FANCI	Forward:	GACGAGCTATTGGATGTTGTCA
	Reverse:	TTCACGAGTTCTCTGCCTAGT
EZH2	Forward:	GGACCACAGTGTTACCAGCAT
	Reverse:	GTGGGGTCTTTATCCGCTCAG
DCN	Forward:	ATGAAGGCCACTATCATCCTCC
	Reverse:	GTCGCGGTCATCAGGAACTT
CDKN1C	Forward:	ACCGTTCATGTAGCAGCAAC
	Reverse:	AGCTTTACACCTTGGGACCA
CENPF	Forward:	CTCTCCCGTCAACAGCGTTC
	Reverse:	GTTGTGCATATTCTTGGCTTGC
BUB1	Forward:	AGCCCAGACAGTAACAGACTC
	Reverse:	GTTGGCAACCTTATGTGTTTCAC
BUB1B	Forward:	GCACCGACAATTCCAAGCTC
	Reverse:	TGTGCTTCGTTGTGGTACAGA
ANLN	Forward:	ATGTCTTCGTGGCCGATTTGA
	Reverse:	CTCTGACAGTGAGTTTCCTGTTT
ASPM	Forward:	CAGACACCCGATGCCATTTG
	Reverse:	TAACCACCAAGTGAAGCCCTG
DDX17	Forward:	CAGGTGGCCGATGACTATGG
	Reverse:	TCGAATCTGGGGACCTTTAGG
KIF11	Forward:	TGTTTGATGATCCCCGTAACAAG
	Reverse:	CTGAGTGGGAACGACTAGAGT
SNAI2	Forward:	CGAACTGGACACACATACAGTG
	Reverse:	CTGAGGATCTCTGGTTGTGGT
RND3	Forward:	TTACACGGCCAGTTTTGAAATCG
	Reverse:	GGGCGGACATTGTCATAGTAAG
CX3CL1	Forward:	CGCGCAATCATCTTGGAGAC
	Reverse:	CATCGCGTCCTTGACCCAT
CD81	Forward:	TTCCACGAGACGCTTGACTG
	Reverse:	CCCGAGGGACACAAATTGTTC
FN1	Forward:	AGGAAGCCGAGGTTTTAACTG
	Reverse:	AGGACGCTCATAAGTGTCACC
ITGB4	Forward:	CTCCACCGAGTCAGCCTTC
	Reverse:	CGGGTAGTCCTGTGTCCTGTA
CDH2	Forward:	TGCGGTACAGTGTAACTGGG
	Reverse:	GAAACCGGGCTATCTGCTCG
ITGA6	Forward:	CAGTGGAGCCGTGGTTTTG
	Reverse:	CCACCGCCACATCATAGCC
EGFR	Forward:	TTGCCGCAAAGTGTGTAACG
	Reverse:	GTCACCCCTAAATGCCACCG
PLCE1	Forward:	GGGTGACATGGCTGATCCTC
	Reverse:	GACAGCGTTGTAGTTGCCCA
ARHGEF9	Forward:	AGTATCCCTTACAGTTGGCTGA
	Reverse:	CGCTTGCGTTCGTTGATCTG
CARF	Forward:	GCACAAATGATGATCGTTGCC
	Reverse:	ACATCCCGAGGACTATTCACAT
SEMA3A	Forward:	CTATCTTCCGAACTCTTGGGCA
	Reverse:	CTTTGGATCATTGAGCCACCT
TRIM52	Forward:	ATGGCTGGTTATGCCACTACT
	Reverse:	CTCGTCCTCCTTACTCCACAG

MUSK	Forward:	CTGGTTGCCTTCAGCGGAA
	Reverse:	CTGCACACATGAAAGTAGCCA
SLC8A1	Forward:	TCATAGCTGATCGGTTCATGTCC
	Reverse:	CAGTTGTCTTGGTGGTCTCTC
DEPDC1	Forward:	ATGCGTATGATTTCCCGAATGAG
	Reverse:	CACAGCATAACACACATCGAGAA
NTSR1	Forward:	AGCAGTGGACTCCGTTCCT
	Reverse:	GTTGGCAGAGACGAGGTTGT
DEPDC1 m^6A peak	Forward:	TGCTTTGGACAGACATGAACCA
	Reverse:	ACCAGTAGGCATAATCAGAACCT
NTSR1 m^6A peak	Forward:	GAACGAGGACCTGGACTCAGA
	Reverse:	AGGCTCAGAAATTATGGCCCAA
ALKBH5 siRNA		GCTGCAAGTTCCAGTTCAA
ALKBH5 shRNA	Forward:	CCGGAAGCTGCAAGTTCCAGTTCAACTCG-AGTTGAACTGGAACTTGCAGCTTTTTTTG
	Reverse:	AATTCAAAAAAAGCTGCAAGTTCCAGTTC-AACTCGAGTTGAACTGGAACTTGCAGCTT
miR-3190 mimic	Forward:	UCUGGCCAGCUACGUCCCCA
	Reverse:	UGGGGACGUAGCUGGCCAGA
miR-3190 inhibitor		UGGGGACGUAGCUGGCCAGA
mimic NC	Forward:	UUUGUACUACACAAAAGUACUG
	Reverse:	CAGUACUUUUGUGUAGUACAAA
inhibitor NC		CAGUACUUUUGUGUAGUACAAA
miR-3190 agomir	Forward:	U*C*UGGCCAGCUACGUC*C*C*C*A
	Reverse:	U*G*GGGACGUAGCUGGC*C*A*G*A
miR-3190 antagomir		U*G*GGGACGUAGCUGGC*C*A*G*A
agomir NC	Forward:	U*U*UGUACUACACAAAAGU*A*C*U*G
	Reverse:	C*A*GUACUUUUGUGUAGUA*C*A*A*A
antagomir NC		C*A*GUACUUUUGUGUAGUA*C*A*A*A

*, Phosphorothioate linkages
